# Self-reported perspective in rare genetic diseases: a systematic review of patient-reported outcome measures classified using the international classification of functioning, disability and health framework

**DOI:** 10.1186/s41687-026-01155-5

**Published:** 2026-07-25

**Authors:** Rachele Simeon, Marina Usai, Alice Tramonti, Renata Canova, Daniela Frigerio, Anna Berardi, Elisa Pelosin, Carola Cosentino

**Affiliations:** 1 Department of Neuroscience, Rehabilitation, Ophthalmology, Genetics and Maternal Child Health (DINOGMI), University of Genoa, Genoa, Italy; 2 Physical Medicine and Rehabilitation Unit, Istituto Giannina Gaslini, Genoa, Italy; 3 Private Practice, Colzate, Italy; 4 Department of Human Neuroscience, Sapienza University of Rome, Rome, Italy; 5 AOM - IRCCS Ospedale Policlinico San Martino, Genoa, Italy

**Keywords:** Genetic rare disease, Patient-reported outcome, PROM, Measurement properties, International classification of functioning, Health-related quality of life

## Abstract

**Background:**

Rare and ultra-rare genetic diseases (GDs) involve complex, multidimensional burdens not fully captured by clinical endpoints, highlighting uncertainties in the availability, scope, and quality of Health-Related Quality-of-life patient-reported outcome measures (HRQoL-PROMs).

**Objectives:**

To identify PROMs developed or validated to assess HRQoL in rare and ultra-rare GDs, map their content using the International Classification of Functioning, Disability and Health (ICF), and evaluate their measurement properties according to COSMIN methodology.

**Methods:**

Original studies reporting PROM development or measurement properties in rare or ultra-rare GDs were included. PubMed, Embase, PsycINFO, Web of Science, registries, outcome-measure repositories, reference lists, and citation tracking were searched from inception to March 26, 2026, without language restrictions. Study selection, data extraction, and risk-of-bias (RoB) assessment were performed independently. Methodological quality was evaluated using the COnsensus-based Standards for the selection of health Measurement Instruments (COSMIN) RoB checklist. Measurement properties were rated as sufficient, insufficient, indeterminate, or inconsistent, and certainty of evidence was assessed using a modified GRADE approach. PROM content was mapped to ICF components and synthesized narratively; no meta-analysis was conducted due to heterogeneity.

**Results:**

Fifty-nine studies were included, covering 45 PROMs across 29 rare or ultra-rare GDs. Instruments were predominantly disease-specific, although generic and adapted measures were also identified. PROMs mainly addressed body functions and activities/participation, while environmental factors, social participation, stigma, and access-to-care domains were consistently underrepresented. Internal consistency and construct validity were most frequently assessed, whereas responsiveness, measurement error, and cross-cultural validity were rarely evaluated. Only three PROMs (HAE-QoL, NF1-AdQoL, EPP-QoL) were classified as COSMIN category A and recommended; most were category B, and five were category C.

**Discussion:**

The evidence is limited and methodologically weak, and many QoL-PROMs fail to capture key multidimensional aspects of QoL; more rigorous, patient-centered, disease-specific development and validation are needed.

**Plain language summary:**

This review examined questionnaires used to assess health related quality of life in people with rare and ultra-rare genetic diseases. Although 45 PROMs were identified, only three had enough evidence to be recommended. Many questionnaires focused mainly on symptoms and physical functioning, while important aspects such as social participation, stigma, care access, and environmental support were often missing. Future PROMs should be developed with strong patient involvement and validated across age groups, languages, and disease contexts.

**Supplementary Information:**

The online version contains supplementary material available at 10.1186/s41687-026-01155-5.

## Introduction

Rare diseases encompass 6,000 to 10,000 distinct conditions that collectively affect an estimated 300 million individuals worldwide [1–3]. In the European context, a rare disease is defined as a condition affecting fewer than 5 in 10,000 individuals in the population, whereas an ultra-rare disease is typically defined as affecting fewer than 1 in 50,000 individuals, according to Decision No 1295/1999/EC of the European Parliament and of the Council [4]. The majority, about 80%, are of genetic origin and frequently manifest in childhood, often with severe and life-limiting consequences [2, 5]. The U.S. Food and Drug Administration’s Patient-Focused Drug Development (PFDD) Guidance [6] underscores the importance of incorporating the patient perspective into medical product development and regulatory decision-making. In this context, Health-Related Quality of Life (HRQoL) represents a critical multidimensional construct, capturing the broad and cumulative impact of Rare and Ultra Rare Genetic Diseases (GDs) on physical functioning, emotional well-being, social participation, and daily life, which are often not fully reflected by clinical or biomarker-based endpoints alone. Accordingly, the use of validated HRQoL-focused PROMs as endpoints in clinical trials and as supporting evidence in regulatory and health policy evaluations provides a robust and standardized approach to capturing patients lived experiences and the real-world burden of rare and ultra-rare GDs.

However, the implementation of PROMs in rare and ultra-rare GDs is hindered by three major challenges. The first relates to the limited knowledge of the availability of instruments, namely, identifying which PROMs exist for specific conditions and the languages and cultural settings in which they have been validated. The second challenge concerns the content of PROMs and the need for conceptual clarity about what these instruments measure. This is particularly relevant in rare and ultra-rare GDs, where clinical heterogeneity affects multiple domains of functioning across conditions with different impact on HRQoL [5, 7]. The World Health Organization (WHO)’s International Classification of Functioning, Disability and Health (ICF) [8] offers a standardized framework for this purpose, enabling the categorization of PROM items into body functions, body structures, activities and participation, and environmental factors. Applying ICF linking rules [9, 10] can enhance comparability across studies and ensure that selected instruments truly capture the aspects of HRQoL most relevant to individuals with rare and ultra-rare GDs. The third challenge relates to the insufficient knowledge of the methodological quality of the available instruments [11]. Evidence on key Measurement Properties (MPs) remains fragmented and inconsistently documented, making it difficult to determine which PROMs evaluating HRQoL can be considered robust and fit-for-purpose in rare and ultra-rare GDs [5, 7].

To overcome these challenges, international initiatives such as the Rare Disease Clinical Outcome Assessment Consortium and the International Rare Diseases Research Consortium (IRDiRC) have called for greater methodological rigor and collaborative development to ensure that new instruments are fit-for-purpose [2, 12]. In parallel, there is increasing consensus on the importance of establishing core outcome sets that incorporate PROMs across domains such as HRQoL [5].

Despite increasing interest, the current evidence base remains fragmented. Existing systematic reviews have largely focused on PROMs within single rare diseases or specific clinical contexts, rather than providing a comprehensive evaluation across rare and ultra-rare conditions [13, 14]. However, to date, no systematic review has comprehensively evaluated the measurement properties of PROMs assessing HRQoL developed or validated across rare and ultra-rare GDs, nor systematically examined their content coverage using a standardized framework such as the ICF.

Therefore, the aim of this systematic review is to identify and evaluate PROMs developed or validated for rare and ultra-rare GDs assessing HRQoL, specifically to (i) Map the population targeted for study validation, the type of PROM, and the languages in which they are available; (ii) Identify the content areas of HRQoL covered by each PROM using the ICF linking rules; and (iii) Evaluate their methodological quality in terms of content validity and other MPs.

## Methods

### Followed guidelines

This systematic review was conducted in accordance with Consensus-based Standards for selection of health Measurement INstruments (COSMIN) methodology for systematic reviews of PROMs [11] and reported following the PRISMA-COSMIN checklist [15]. The protocol was registered into the International Prospective Register of Systematic Reviews [PROSPERO: CRD420251000211] and is publicly available.

### Eligibility criteria

Original studies reporting the development and/or validation of PROMs in rare and rare and ultra-rare GDs, as well as studies assessing one or more MPs of these instruments were included. Studies were eligible for inclusion when the following criteria were met: (i) **Construct of interest**: HRQoL in terms of ICF component (Body functions, Activities and Participation, Environmental factors or Body structures). (ii) **Population**: The study population consisted of individuals with a confirmed diagnosis of a rare or ultra-rare GDs. Studies including heterogeneous samples composed of multiple rare genetic conditions were excluded if results were not reported separately for each specific disease. Studies administering a PROM across broad or aggregated disease groups (e.g., neuromuscular disorders) without providing disease-specific psychometric analyses (e.g., for Duchenne muscular dystrophy) were excluded, as this approach does not allow for the evaluation of MPs at the level of a single condition. (iii) **Instrument type**: The instrument was a self-reported PROM. (iv) **MPs**: At least one aim of the study was the evaluation of one or more MPs or the interpretability of a PROM, such as: content validity, structural validity, internal consistency, reliability, measurement error, cross-cultural validity, criterion validity, construct validity and responsiveness. Studies that only used a PROM without evaluating its MPs were excluded.

### Information sources

A comprehensive literature search was conducted in the following electronic databases: PubMed, Embase, Psychinfo and Web of Science (Supplementary material 1), from inception to March 26^th^, 2026. No language restrictions were applied. To ensure completeness, additional sources were consulted, including study registries (PROSPERO, ClinicalTrials.gov) and repositories and databases of outcome measurement instruments (COSMIN database). Reference lists of included studies were screened manually, and citation tracking was performed using PubMed. When full texts were unavailable, corresponding authors were contacted. Where available, PROM user manuals, scoring protocols, and other supporting materials of the identified PROMs were retrieved and evaluated to supplement the information from primary studies.

### Search strategy

The search strategy was designed to capture studies reporting the development or validation of PROMs in rare and ultra-rare GDs. The conceptual structure comprised three main components: (i) population (rare and ultra-rare diseases, genetic disorders), (ii) instrument (PROMs and related terms), and (iii) MPs (validity, reliability, responsiveness). No limits were applied regarding language or publication date, in line with the eligibility criteria. Line-by-line search strategies as executed in each database are provided in Supplementary Material 1. Search strategies were adapted to the specific syntax of each database interface (PubMed, PsychINFO, Web of Science and Embase). Validated methodological filters for MPs (e.g. COSMIN search filter) were used as a reference and adapted for this review. The final search strategy underwent peer review by author A.B., an information specialist, following the PRESS (Peer Review of Electronic Search Strategies) checklist, to ensure completeness and accuracy. The strategy was further validated by checking whether it retrieved a set of key eligible studies known a priori (C.C.).

### Selection process

All retrieved records were imported into Rayyan QCRI [16] and duplicates were removed. Two reviewers (M.U. and R.S.) independently screened titles and abstracts for eligibility. Full texts of potentially eligible studies were then independently assessed by three reviewers (M.U., R.S. and D.F.). Discrepancies at any stage were resolved through discussion and, when necessary, consultation with a senior expert (C.C.

### Data collection process and data items

Data extraction was undertaken independently by two reviewers (R.S. and M.C.) using a pre-prepared data extraction sheet. Discrepancies at any stage were resolved through discussion and, when necessary, consultation with a senior expert (C.C.). The data extraction sheet was first piloted (on two development paper articles and two measurement property articles), before being revised for further use. The following data items were extracted from each included study: Author, year; Disease; Scale (acronym); Language (Country); Sample Size; Mean age (SD); Male (%); Construct; Item (n°); Likert-scale. The data extraction table is reported in Table 1. No modifications were made compared to the protocol in terms of data extraction, and the same predefined data extraction framework was applied.

**Table 1:** Characteristics of the included studies and PROMs

Author, year	Disease	Scale (acronym)	Language (Country)	Sample Size	Mean age (SD)	Male (%)	Item	Likert
**Connective Tissue and Skeletal Disorders**								
Anttila, 2021	Skeletal Dysplasia	Questionnaire for People with Skeletal Dysplasias	Finnish (FI)	14	n.a. (>9)	n.a.	8	4
Godeau, 2023	Low Flow Malformations	Children Low Flow Malformations Quality of Life (cLFM-QoL)	French, English (FR; n.a.)	75	13 (1.33)	35 (46.7)	38	5
**Dermatological Disorders**								
Crawford, 2022	Neurofibromatosis Type 1 (NF1)	NF1 Adult Health-Related QoL (NF1-AdQOL)	English (AU)	8 concept elicitation; 10 pre-test; 114 validation	27.5 (18–40)	48 (42)	31	5
De Vries, 2018	Tuberous sclerosis complex	QoL in Epilepsy Inventory for Adolescents-48 (QOLIE-AD-48)	n.a.	37	n.a. (4–21)	n.a.	10	dichotomous
		QoL in Epilepsy Inventory-31-Problems (QOLIE-31-P)	n.a.	33	n.a. (4–21)	n.a.	85	4
Gomide, 2013	Hereditary Angioedema (HAE)	36item ShortForm Health Survey (SF36)	Portuguese (BR)	35	10 (28.6)	n.a.	36	Dichotomous; 0–4; −10–10
Jahanshahi, 2023	Neurofibromatosis Type 1	NF1 Adult Health-Related QoL (NF1-AdQOL)	Persian (IR)	Concept elicitation: 40; Validation: 414; test-retest reliability: 30	34.4 (8.3)	124 (30)	31	5
Jindal, 2017	Hereditary Angioedema (HAE)	36item ShortForm Health Survey (SF36)	English (US)	21	1 (4.8)	42.3 (13.7)	36	Dichotomous; 0–4; −10–10
Kügler, 2026	Hypohidrotic ectodermal dysplasia (HED)	HED HRQoL	German (DE), Austrian (AU)	24	10 (4)	18 (75)	83	n.a.
McLean, 2013	Neurofibromatosis type 2	Neurofibromatosis Quality of Life (NFTI-QOL)	English (UK), German (DE)	77	38 (16–68)	28 (36.36)	14	7 item: 4; 1 item: 3; 6 item: dichotomous
Nutakki, 2013	Neurofibromatosis Type 1	Pediatric Quality of Life Inventory (PedsQL™)	English (US)	124	40.2 (20–71)	n.a.	34	5
Oliveira Machado, 2026	Neurofibromatosis type 1	Pediatric Quality of Life Inventory (PedsQL™)	Portuguese (BR)	42	10.5 (5–25)	21 (42.9)	48	5
Palao-Ocharan. 2022	Hereditary Angioedema (HAE)	36item ShortForm Health Survey (SF36)	n.a (ES, DE,UH, BR, DK, PL,CA, RO, AT, AR, IL)	290	41.5 (14.6)	90 (31)	36	Dichotomous; 0–4; −10–10
Prior, 2012	Hereditary Angioedema (HAE)	HAE-QoL	Spanish (ES)	45	16 (35.6)	39 (18–74)	44	4
Prior, 2016	Hereditary Angioedema (HAE)	HAE-QoL	Danish, English, French, German, Hebrew, Hungarian, Italian, Macedonian, Mandarin, Chinese, Polish, Portuguese, Romanian, Spanish (AR, AT, BR, CN, DE, DK, ES, FR, HU, IL, IT, MK, PA, PL, RO, UK, US)	290	41.5 (14.6)	90 (31)	n.a.	4
Salamon, 2024	Epidermolysis Bullosa	QoLEB	German (DE)	46	45.89 (19.29)	13 (28.3)	52	5
Vanya, 2023	Hereditary Angioedema (HAE)	Angioedema Quality of Live Questionnaire (AE-QoL)	English (CA)	40	5 (12)	39 (18–66)	17	5
Villar Hernández, 2022	Epidermolysis Bullosa	QoLEB	Spanish (ES)	33	38(18–83)	11 (33)	17	3
Yazdanshenas, 2020	Epidermolysis Bullosa	QoLEB	Farsi (IR)	83	15 (3–43)	43 (48.2)	17	4
Yuen, 2014	Epidermolysis Bullosa	QoLEB	Dutch (NL)	83	47.6 (17.1; 19–85)	28 (51)	17	4
**Hematologic And Immunological Disorder**								
Asnani, 2009	Sickle Cell Disease	36item ShortForm Health Survey (SF36)	English (JM)	491	31.3 (9.6; 18–70)	210 (42.7)	36	Dichotomous; 0–4; −10–10
		Flanagan’s quality of life scale (QOLS)					16	5
		World Health Organization Quality of Life - BREF (WHOQOL-BREF)					74	5
Biolcatti, 2021	Erythropoietic Protoporphyria	Erythropoietic Protoporphyria Quality of Life (EPP-QoL)	English (UK)	355	n.a.	n.a.	15	4
Groth, 2016	Paroxysmal Nocturnal Hemoglobinuria and aplastic anemia	aplastic anemia and/or paroxysmal nocturnal hemoglobinuria-specific QoL (QLQ-AA/PNH)	German (DE), Swiss (CH)	Phase 1: 19; Phase 2: 30	42.1 (n.a.)	Phase 1: 4 (21); Phase 2: 15 (50)	97	5
Mathias, 2024	Erythropoietic Protoporphyria	Erythropoietic Protoporphyria Impact Questionnaire (EPIQ)	English (US)	23	n.a. (12–61)	12 (52.2)	10	5
Niedeggen, 2019	Paroxysmal Nocturnal Hemoglobinuria and aplastic anemia	aplastic anemia and/or paroxysmal nocturnal hemoglobinuria-specific QoL (QLQ-AA/PNH)	German (DE)	48	49 (23–83)	19 (40)	54	5
Oladapo, 2023	Congenital thrombotic thrombocytopenic purpura (cTTP)	Congenital Thrombotic Thrombocytopenic Purpura–Patient Experience Questionnaire (cTTP-PEQ)	English (UK)	36	36.3 (13.2; 0–70)	12.0 (33.3)	37	4
Solovieva, 2004	Hereditary Blood Coagulation Disorder	36item ShortForm Health Survey (SF36)	Finnish (FI)	164	42 (15; 16–75)	131 (80)	69	5
Spolak-Bobryk, 2022	Mastocytosis	Quality of Life in Mastocytosis Scale (QLMS)	Polish (PL)	85	45.3 (10.8)	28 (32.9)	24	6
Turc, 2022	Hereditary hemorrhagic telangiectasia (HHT)	Quality of Life-Hereditary hemorrhagic telangiectasia (QoL-HHT)	French (FR)	228	53 (16.5)	78 (30.7)	20	7
Weisshaar, 2020	Paroxysmal Nocturnal Hemoglobinuria and aplastic anemia	PRO-AA/PNH	Swiss (CH)	31 (delphi)	n.a.	n.a.	30	4
Xu, 2021	Hemophilia (HE)	EQ-5D-5 L with two bolt-on item	Chinese (CN)	895	n.a. (>18)	895 (100)	36	5
Xu, 2021	Hemophilia (HE)	EuroQoL-5D (EQ-5D)	Chinese (CN)	875	32.9 (9.5)	875 (100)	12	11
		ShortForm 6D (SF-6D)	Chinese (CN)	875	32.9 (9.5)	875 (100)	12	11
**Metabolic and Lysosomal Storage Disorder**								
Elstein, 2022	Gaucher disease	Gaucher Disease Patient reported outcome measures (GD- PROMs)	English (UK)	46	n.a. (>25)	23 (50.0)	65	5
Iversen, 2018	Lymphedema cholestasis syndrome 1 (LCS1)	36item ShortForm Health Survey (SF36)	Norwegian (NO)	18	n.a. (18–65)	11 (61)	36	Dichotomous; 0–4; −10–10
Jurecki, 2017	Phenylketonuria (PKU)	Phenylketonuria – Quality Of Life (PKU-QOL) - Children version	English (US)	5	9.8 (9–11)	2 (40)	26	5
		Phenylketonuria – Quality Of Life (PKU-QOL) - Adolescent Version	English (US)	5	14.8 (12–17)	4 (80)	15	6
		Phenylketonuria – Quality Of Life (PKU-QOL) - Adult Version	English (US)	5	28.4 (19–36)	2 (40)	24	5
Kanters, 2015	Pompe Disease	EuroQoL-5D (EQ-5D)	Dutch (NL)	80	49.4 (n.a.)	37 (46)	20	5
		ShortForm 6D (SF-6D)	Dutch (NL)	80	49.4 (n.a.)	37 (46)	39	5
Koto, 2022	Fabry disease	Adult Fabry Disease Quality Of Life (AFQOL)	Japanese (JP)	83	52.2 (14.90)	26 (31.3)	17	4
Narita, 2024	Gaucher disease	Gaucher Disease Patient reported outcome measures (GD- PROMs)	Japanese (JP)	33	32.3 (24.6)	16 (48.5)	20	5
Regnault, 2015	Phenylketonuria (PKU)	Phenylketonuria – Quality Of Life (PKU-QOL) - Children version	English (UK), French (FR), German (DE), Spanish (ES), Dutch (NL), Turkish (TR)	92	9.8 (0.8; 9–11)	43 (46.7)	28	n.a.
		Phenylketonuria – Quality Of Life (PKU-QOL) - Adolescent Version		110	14.5 (1.6; 12–17)	56 (50.9)	11	5
		Phenylketonuria – Quality Of Life (PKU-QOL) - Adult Version		104	25.8 (6.6; 18–45)	38 (36.5)	30	6
Truninger, 2024	Pompe Disease	Pompe Qualty of Life 1.0 questionnaire (Pompe QoL)	German (CH, AT, DE)	16	14.3 (4; 8–20)	11 (68.75)	11	4
van der Beek, 2013	Pompe Disease	Rasch-Built Pompe-Specific Activity (R-Pact)	Dutch (NL, BE); English (US, UK, CA)	186	n.a.	91 (49)	18	3
van Kooten, 2024	Pompe Disease	Modified Rasch-Built Pompe-Specific Activity (mR-Pact)	Dutch (NL, BE); English (UK, US, NZ, AU, CA); French (FR); German (DE, CH); Italian (IT); Spanish (ES); Portuguese (PT)	186	51.6 (14.2)	248 (47)	18	3
Vinik, 2014	Transthyretin Familial Amyloid Polineuropathy (ATTR-FAP)	Norfolk Quality of Life-Diabetic Neuropathy Questionnaire(QoL-DN)		61	n.a.	31 (50.8)	15	4
**Neuromuscolar and Neurodegenerative Disorder**								
Carlozzi, 2014	Hungtington disease (HD)	Huntington’s Disease Patien Reported Outcome-TRIAD (HD-PRO-TRIAD)	English (US)	132	40.8 (11.4)	69 (52)	47	5
Carlozzi, 2016	Hungtington disease (HD)	Huntington disease Quality of Life (HDQLIFE)	English (US)	536	48.7 (13.31)	220 (41)	7	5
Clay, 2012	Hungtington disease (HD)	Huntington Quality of Life Instrument (H-QoL-I)	French,Italian (FR, IT)	252	54.5 (11.6; 16–80)	124 (49)	15	4
Crossnohere, 2021	Duchenne Muscular Dystrophy (DMD)	EuroQoL-5D (EQ-5D)	English (US, CA, AU, DE, UK, BE)	61	28.2 (18–48)	61 (100)	5	5
Landfeldt, 2018	Duchenne Muscular Dystrophy (DMD)	Pediatric Quality of Life Inventory 3.0 Neuromuscular Module (PedsQL NMM)	English (US, UK)	278	16 (7)	278 (100)	1	11
Malina, 2024	Hereditary Spastic Paraplegia	Health-related Quality Of Life in Hereditary Spastic Paraplegia (TreatHSP-QoL)	English (UK), german (DE)	Concept elicitation: 36; Pre-test: 91; Validation 242	53 (18–74)	21 (58)	56	4
Moroni, 2022	Charcot Marie Tooth (CMT)	pediatric Charcot-Marie-Tooth Quality of Life (pCMT-QoL)	Italian (IT)	22	14.27 (2.51)	11 (50	n.a.	n.a.
Pisciotta, 2020	Charcot Marie Tooth (CMT)	Charcot-Marie-Tooth Health Index (CMHI)	Italian (IT)	30	48.0 1(6.4)	16 (52)	n.a.	n.a,
Ramchandren, 2021	Charcot Marie Tooth (CMT)	pediatric Charcot-Marie-Tooth Quality of Life (pCMT-QoL)	English (US)	398	12 (8–18)	196 (54.8)	n.a.	n.a.
Tremblay, 2024	Autosomal recessive cerebellar ataxias (ARCAs)	Person-Reported Ataxia Impact Scale (PRAIS)	French, English (CA, US, UK)	105	Concept elicitation: 39.3 (18–66)	Concept elicitation: 6 (50)	18	5
		Person-Reported Ataxia Impact Scale (PRAIS)	French, English (CA, US, UK)	105	Validation: <18	Validation: 36 (34)	18	5
**Ophthalmologic Disorders**								
Audo, 2023	Leber Congenital Amaurosis (LCA)	Visual Symptom and Impact Outcomes patient-reported outcome (ViSIO-PRO)	n.a. (US, CA, FR, DK, DE)	development	n.a.	n.a.	44	11
Fischer, 2023	Retinitis Pigmentosa (RP)			83	37.8 (12–79)	49 (59.0)	49	
Kay, 2023				66	n.a.	n.a.	44	
**Respiratory disorders**								
Behan, 2017	Primary Ciliary Dyskinesia	Quality of Life Primary Ciliary Dyskinesia (QOL-PCD)	English (UK, US, CA)	72	33 (18–79)	23 (31.95)	58	5
Behan, 2019		Quality of Life Primary Ciliary Dyskinesia (QOL-PCD) - adolescents version	English (US, UK, CA, IE)	71; 17 test-retest reliability	9.5 (6–12)	36 (50.7)	12	11
		Quality of Life Primary Ciliary Dyskinesia (QOL-PCD) - children version	English (US, UK, CA, IE)	85 validation; 13 test-retest reliability	15.4 (13–17)	44 (51.8)	n.a.	3
Dell, 2016		Quality of Life Primary Ciliary Dyskinesia (QOL-PCD) - adolescents version	English (US, UK, IE, CA)	69	n.a. (6–17)	34 (49.28)	14	11
		Quality of Life Primary Ciliary Dyskinesia (QOL-PCD) - children version	English (US, UK, IE, CA)	69	n.a. (6–17)	34 (49.28)	82	4
Lucas, 2015		Quality of Life Primary Ciliary Dyskinesia (QOL-PCD)	English (UK-US)	21	n.a. (>18)	3 (14)	40	5

### Synthesis methods

The rare and ultra-rare GDs identified in the included studies were classified according to a phenotypic, anatomical-clinical criterion, grouping conditions based on the primarily affected organ system or functional domain (e.g., neuromuscular, metabolic, hematologic). Each item of the PROMs was linked to the corresponding ICF code according to established linking rules [9, 10]; based on these codes, we identified the chapters and, subsequently, the ICF components (body functions (b), activity and participation (d), environmental factors (e), body structures (s)) to which each PROM was categorized under. The detailed coding is provided in Supplementary Material 5.

A bubble plot was developed to visually synthesize the evidence on PROMs across rare and ultra-rare GDs. The horizontal axis (X-axis) represents the ICF components. While the reference framework consists of the four main components (b, s, d, e), additional combined categories were generated whenever a PROM included items classifying into more than one ICF component. Consequently, the X-axis may include both single and combined categories, depending on the content of the PROMs. The vertical axis (Y-axis) lists each PROM alphabetically. Bubble size reflects the sample size (logarithmic scale applied), bubble color indicates the number of ICF chapters covered by each PROM, thus capturing the content coverage of the instruments. To provide evidence-based recommendations, PROMs classified as “A” (recommended) were highlighted in the bubble plot with a blue outline surrounding the corresponding circle, whereas PROMs classified as “C” (not recommended) were indicated using the same blue outline around circles labeled “C” ^11^. This visual coding highlights the instruments that met the highest methodological and clinical standards according to the systematic evaluation.

### Risk of bias assessment

The methodological quality of the included studies was evaluated using the COSMIN Risk of Bias (RoB) checklist [17]. The checklist covers ten aspects: PROM development, content validity, structural validity, internal consistency, cross-cultural validity/measurement invariance, reliability, measurement error, criterion validity, hypotheses testing for construct validity, and responsiveness. PROMs with insufficient content validity were not considered suitable for further evaluation of additional measurement properties, in accordance with COSMIN recommendations [11]. These instruments were retained for descriptive purposes only, and their subsequent measurement properties were interpreted with caution. Regarding construct validity, PROMs were assumed to follow a reflective measurement model, whereby the underlying construct gives rise to the observed item responses. Accordingly, construct validity was evaluated in line with COSMIN guidance using hypothesis testing and structural validity analyses.

Each domain was rated on a four-point scale (“very good,” “adequate,” “doubtful,” “inadequate”), applying the “worst score counts” principle. Two reviewers (RS; MU) independently assessed each study, and any discrepancies were resolved through discussion and, when necessary, consultation with a senior expert (CC).

### Measurement properties

Results from individual studies on MPs were evaluated according to COSMIN’s updated criteria for good MPs. Each result was rated as sufficient (+), insufficient (−), or indeterminate (?). Content validity was assessed for relevance, comprehensiveness, and comprehensibility. For construct validity and responsiveness, a priori hypotheses were formulated following COSMIN guidance: correlations ≥ 0.50 were expected with instruments measuring similar constructs; correlations < 0.50 but ≥0.30 with related constructs; and correlations < 0.30 with unrelated constructs. Criterion validity was considered only when a shortened PROM was compared with its validated full version [18]. Evidence from multiple studies on the same PROM or subscale was summarized per measurement property. If ≥ 75% of the studies showed consistent results, the measurement property was rated accordingly (sufficient or insufficient). If < 75% agreement was observed, the overall rating was classified as inconsistent (±). This rule was applied across all MPs. No adaptations were made to the COSMIN criteria. Two reviewers (RS; MU) independently rated each measurement property for each study, and discrepancies were resolved through discussion and consensus.

### Certainty of evidence

The certainty of evidence was assessed using a modified GRADE approach (Supplementary material 6), accounting for RoB (Supplementary material 2), consistency of results, directness, and imprecision related to sample size. Certainty was categorized as high, moderate, low, or very low. Two reviewers (RS; CC) independently assessed the certainty, and discrepancies were resolved through discussion and consensus. This evaluation reflects confidence that the synthesized results for each measurement property are trustworthy. Instruments were classified as follows: (i) Category A, with sufficient content validity (any level) and at least low-quality evidence for internal consistency; (ii) Category B, not meeting criteria for A or C; and (iii) Category C, with high-quality evidence for an insufficient MP. PROMs in Category A can be recommended for use, while those in Category B may be provisionally considered pending further evidence. Category C instruments are not recommended [11].

### Results

### Study selection

**Fig. 1:**
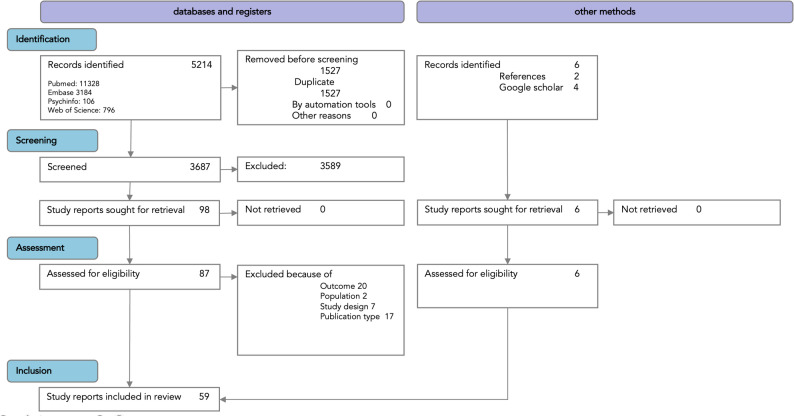
Flowchart of the study selection process. *From:* Elsman EBM, et al. Guideline for reporting systematic reviews of outcome measurement instruments (OMIs): PRISMA-COSMIN for OMIs 2024. *quality of Life Research* (2024)

The electronic search retrieved 5214 records. After removal of duplicates (*n* = 1527), 3687 records remained for title and abstract screening. Of these, 3589 were excluded for not meeting the eligibility criteria. The full text of 87 potentially eligible articles was retrieved and assessed in detail. Following full-text review, 46 studies were excluded, most commonly due to ineligible population, inappropriate instrument type, or absence of evaluation of MPs. The selection process is presented in the PRISMA-COSMIN flow diagram (Fig. 1). A complete list of excluded full-text studies, with citations and reasons for exclusion, is available in Supplementary Material 2. We contacted two corresponding authors to retrieve missing materials (one to obtain the full text of an article and one to request access to the PROM instrument); all authors responded.

### Studies and PROMs characteristics and RoB assessment

In total, 59 studies were included, covering 45 unique PROMs across 29 rare or ultra-rare GDs. Validation studies were predominantly conducted in English (50.9%). The instruments were grouped into four categories by specificity: generic instruments (*n*=6); disease-specific tools originally developed for other conditions but applied to rare-disease populations (*n*=5); generic measures augmented with rare-disease–relevant items (*n*=1); and rare disease–specific instruments explicitly designed for a particular rare condition (*n*=33). The most frequently evaluated MPs assessed were internal consistency (*n*=156) and construct validity (*n*=120), followed by reliability (*n*=78), content validity (*n*=51), measurement error (*n*=28) and structural validity (*n*=27). In contrast, responsiveness (*n*=17) and cross-cultural validity (*n*=12) were infrequently assessed. Detailed characteristics of included studies are presented in Table 1.

Based on the diseases identified, we categorized them into seven groups according to a phenotypic anatomical-clinical criterion: (1) Connective Tissue and Skeletal Disorders, (2) Dermatological Disorders, (3) Hematologic and Immunological Disorders, (4) Metabolic and Lysosomal Storage Disorders, (5) Neuromuscular and Neurodegenerative Disorders, (6) Ophthalmologic Disorders, and (7) Respiratory Disorders.

### Connective Tissue and Skeletal Disorders

In the category of Connective Tissue and Skeletal Disorders, two conditions were represented: Low Flow Malformations (LFM) [19] and Skeletal Dysplasia [20].

Two validated PROMs were identified for these conditions [21]: Children Low Flow Malformations Quality of Life (cLFM-QoL) [19] and Questionnaire for People with Skeletal Dysplasias [20]. Validations were conducted in French & English [19] and Finnish [20]. Sample sizes were 75 (adolescents) [19] and 14 [20] (children), respectively. Content validity was assessed in both instruments [19, 20], with doubtful RoB. Other MPs were assessed only for Children Low Flow Malformations Quality of Life (cLFM-QoL) [20]: Structural validity was assessed using confirmatory factor analysis (CFA); however, the sample size was limited (five patients per item), resulting in a doubtful rating despite good model fit. Internal consistency and construct validity were rated as sufficient (+), with very good methodological quality. Reliability and measurement error were rated as sufficient and indeterminate, respectively, with doubtful methodological quality due to unclear reporting on whether measurement conditions were comparable.

### Dermatological Disorders

Among Dermatological Disorders, six conditions were represented, including Hereditary Angioedema (HAE), Hypohidrotic ectodermal dysplasia (HED), Neurofibromatosis Type 1 and 2 and Tuberous Sclerosis Complex (TSC) and Inherited Epidermolysis Bullosa (EB). For these conditions, ten validated PROMs were identified [21–37]. Validations were performed in English [21, 25–28, 30, 32, 34], Portuguese [23, 29], Persian [24], German [26, 30, 33], Spanish [31, 32, 35], Farsi [36] and Dutch [37]. Sample sizes ranged from 8 to 290, with eight studies conducted in adults [21, 24, 25, 30, 32, 33, 35, 37] and ten [22, 23, 25–27, 29, 31, 34, 36] in children and adolescent.

Content validity was assessed in thirteen studies [21, 24, 26–29, 31–36] results ranged from sufficient (+) with an adequate RoB [26, 32] to insufficient (−) [32, 34] or indeterminate (?) [27, 28, 33, 35] with doubtful RoB. Structural validity was evaluated in six studies [21, 24, 28, 32, 33, 35]. Ratings were mostly indeterminate (?), with Doubtful RoB [21, 24, 28, 33, 35]. One PROM was rated insufficient with Doubtful RoB [28], due to insufficient sample size. Internal consistency was assessed in all included studies, except for three [26, 29, 31]. Many achieved sufficient (+) results with very good RoB. One study was rated sufficient with a doubtful RoB [32]. Only three PROMs were rated insufficient (-) with a very good rating [22, 23]. Reliability was evaluated in ten studies [21, 29, 30, 32, 35, 37], including twenty-two scales and subscales. In two studies [32, 34], was rated as sufficient (+) with an adequate RoB. In seven studies [21, 30, 35, 37], it was rated as sufficient (+) but with a doubtful RoB due to the unclarity of patients’ stability, whereas in one study [29] reliability was rated as insufficient (−) with an inadequate RoB. Measurement error was assessed in only two studies [30, 34], both yielding indeterminate (?) ratings despite an adequate RoB. Construct validity was tested in eleven studies [21, 22, 27, 30, 32–38] ratings ranged from sufficient (+) with very good/adequate RoB [27, 30, 32–34, 37] to doubtful RoB [33, 35, 36], when hypotheses were not prespecified or comparators had no specified MPs, and rated “insufficient” when hypotheses were not met with inadequate RoB [22]. Responsiveness was seldom investigated, with only two studies [34, 39], one of them was rated an adequate RoB with sufficient rating [34].

### Hematologic and Immunological Disorders

Among of Hematologic and Immunological Disorders, eight conditions were included: Congenital thrombotic thrombocytopenic purpura (cTTP) [40], Erythropoietic Protoporphyria (EP) [41, 42], Hemophilia (HE) [43, 43], Hereditary hemorrhagic telangiectasia (HHT) [44], Mastocytosis (M)^46^, Paroxysmal Nocturnal Hemoglobinuria (PNH) [45], and Sickle Cell Disease (SCD) [46–49]. Across these conditions, twelve validated PROMs were identified [40–43, 43, 44, 49–53]. Validations were conducted in English [40–43, 43, 44, 49, 50], French [44], Chinese [43, 43], German [51, 52], Polish [50]and Swiss [52]. Sample sizes ranged from 23^42^ to 895^43^, with all studies conducted in adults except one conducted also in adolescents [42]. Content validity was assessed in six studies [42–44, 51–53], three assessed as sufficient (+) and adequate RoB [42–44, 51–53] and three as indeterminate (?) and doubtful [42–44, 51–53] due to a lack of clarity regarding the performance of focus groups and inconsistencies in the transparency of concept elicitation and cognitive debriefing processes. Structural validity was assessed in three instruments [41, 44, 50]: Two were assessed as sufficient rating (+), one with adequate RoB [44] and one with inadequate due to the sample size, while the third one [41] was rated insufficient (-) with doubtful RoB due to the sample size (five patients per item). Internal consistency was examined in five studies [40, 41, 44, 49, 50] including fifteen scales and subscales, all of them assessed with a very good RoB and rated as sufficient (+) except for WhoQoL-Breef Social Relations Subcale [49] that was rated as insufficient (-). Cross-cultural validity was evaluated in one study and rated sufficient (+) with a very good RoB [41].

Reliability was assessed in three studies, two rated sufficient (+) with and inadequate [44] or very good [40]RoB and insufficient (-) and inadequate RoB [41]. Construct validity was assessed in six studies [41, 43, 43, 44, 49, 50], most of them rated insufficient (-) [41, 49, 50], with very good [49] or doubtful RoB [41, 50]. Four PROMs were rated as sufficient (-) two with an adequate RoB [43, 44], one with very good RoB [43] and one with doubtful RoB [41].

### Metabolic and Lysosomal Storage Disorders

Among Metabolic and Lysosomal Storage Disorders, six conditions were included: Fabry disease (FD) [54], Gaucher disease (GD) [55, 56], Lymphedema cholestasis syndrome 1 (LCS1) [57], Phenylketonuria [58, 59], Pompe Disease (PD) [60–62], Transthyretin Familial Amyloid Polineuropathy [63]. Twelve validated PROMs were identified [54–64]. Validations were conducted in English [56, 58, 59], French [58], Japanese [54, 55], German [61], Dutch [60, 62, 64] and Norvegian [57]. Sample sizes ranged from 5^55^ to 186^61,63^, most of the studies were conducted in adults [54–64], only three were conducted in children and adolescence [58, 59, 61]. For each PROM, the MPs assessed were as follows: content validity was assessed in six instruments [55–59, 61, 62, 64], most with a doubtful RoB [55, 56, 58–61, 63, 65–68] due to incomplete reporting of development procedures and unclear composition of focus groups for concept elicitation and cognitive debriefing, limiting appraisal of relevance, comprehensiveness, and comprehensibility [54–64]. Only one study was evaluated as adequate RoB [64]. Structural validity was evaluated in three studies, one rated insufficient (-) with an inadequate RoB [63], the others rated as indeterminate (?) with an adequate [60] or inadequate RoB [54]. Internal consistency was reported in twelve scales and subscales, all of them rated very good except for one study evaluated inadequate [56]. Four PROMs were rated as insufficient (-) [56, 58], six as sufficient (+) [54, 55, 60] and two with indeterminate (?) [54, 57]. Cross-cultural validity was assessed in one study [55, 60] rated as sufficient with a doubtful RoB [60]. Reliability was evaluated in seven between scales and subscales, only one rated sufficient (+) with doubtful RoB [55], and one rated indeterminate (?) with doubtful RoB [61] and the others rated insufficient (-) with RoB ranged from adequate to inadequate. Measurement error was assessed in one study [54, 60] rated with sufficient (+) and doubtful RoB. Criterion validity was inadequate in two PROMs where it was assessed, owing to the absence of comparisons with a gold standard [54].

Construct validity was investigated in ten PROMs, five of them were rated insufficient (-) with a RoB ranging from very good [57] to inadequate [56, 64]. Only two studies [54, 63] were rated sufficient (+) with very good or adequate RoB. Three PROMs were rated indeterminate (?) with a doubtful RoB [58].

### Neuromuscular and Neurodegenerative Disorders

Among Neuromuscular and Neurodegenerative Disorders, five conditions were included: Autosomal recessive cerebellar ataxias (ARCAs) [69], Charcot Marie Tooth (CMT) [70, 71], Duchenne muscular dystrophy (DMD) [72, 73], Hereditary Spastic Paraplegia (HSP) [74], Huntington disease (HD) [75–77]. For these conditions, nine validated PROMs were identified. Validations were performed in English [69, 71–76], French [69, 77] and Italian [77]. Sample sizes ranged from 22 to 536; all of them were assessed in adults’ populations except for three evaluated in adolescents [71, 73]. Content validity was evaluated in six studies [69–72, 74, 76, 77], showing heterogeneous results and frequent methodological concerns. Only one study [74] was rated as sufficient (+) and the RoB was assessed doubtful. The other were rated indeterminate (?) with doubtful or inadequate [71, 72] RoB. Internal consistency wasn’t evaluated in only three studies [70, 72]. All studies were evaluated very good with three studies rated as indeterminate (?) [70, 71, 76], one insufficient (-) [74]and the other sufficient (+). Reliability was evaluated across four PROMs [70, 71, 74]; all were rated as doubtful, except one [70], which was rated as inadequate due to non-comparable measurement conditions or insufficient reporting of the ICC model. Despite these limitations, all studies were rated as sufficient (+).

Construct validity was assessed in twenty-two scales and subscales: one study was rated sufficient (+) with very good RoB [72]; two studies were rated sufficient (+) with adequate RoB [71, 76]; and two studies were rated sufficient (+), one with doubtful RoB [69, 77] and one with inadequate RoB [17].

### Ophthalmologic Disorders

Among Ophthalmologic Disorders, two conditions were represented: Leber Hereditary Optic Neuropathy and Retinitis Pigmentosa [78–80]. For these conditions, one validated PROM was identified, Impact Outcomes patient-reported outcome (ViSIO-PRO). Language of validation was not reported. Sample sizes were 66 [79] and 83 [81] participants, with both studies conducted in adult populations.

Content validity was assessed indeterminate (?) with doubtful RoB [78, 80]. Structural validity was rated indeterminate (?) [79], while internal consistency was rated sufficient (+) with a very good RoB. Reliability, measurement error, and construct validity were assessed only in Fischer et al. [79], with ratings of sufficient (+), sufficient (+), and inadequate (–), respectively. The methodological quality was judged very good for reliability and measurement error, and inadequate for construct validity.

### Respiratory Disorders

In the category of Respiratory Disorders, one condition was represented: Primary Ciliary Dyskinesia [82–85]. Three version of the same PROM were identified: the QoL–Primary Ciliary Dyskinesia (QOL-PCD), validated in adults as well as in adolescents and children. The QOL-PCD was validated in English [82–85] with sample sizes ranged from 21 to 85 participants [82, 84]. Content validity was rated as indeterminate (?) and assessed doubtful in two of them [82, 83]. Internal consistency was evaluated in all studies as very good: in one study the children’s version was rated insufficient (-) while all the others were rated as sufficient (+). Reliability was assessed in two studies, all rated sufficient (+) and assessed as adequate or very good RoB [83]. Measurement error was evaluated in one study, rated as indeterminate (?), with methodological quality judged as adequate in Behan et al. [84]. Construct validity was evaluated in twenty-six scales and subscales, with very good or adequate RoB. Hear and hearing symptoms subscales (adult version) was rated insufficient (-) such as all subscales from Behan et al. [84] study, except for “Role subscale” adolescent version.

Detailed study-level results, including extracted findings, ratings against COSMIN criteria, and corresponding RoB assessments, are presented in Supplementary Material 3.

### Results of syntheses

**Fig. 2:**
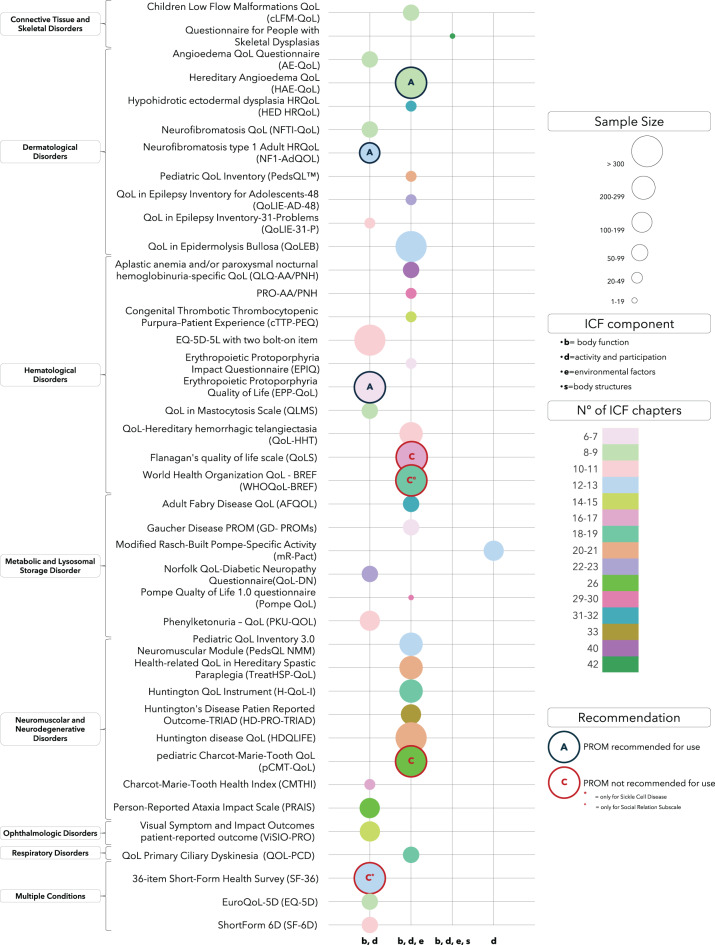
Bubble plot summarizing the characteristics of patient-reported outcome measures assessing quality of life in individuals with rare genetic diseases

The bubble plot (Fig. 2) illustrates the distribution of PROMs across rare and ultra-rare GDs in relation to ICF components. On the X-axis, PROMs are grouped into four categories reflecting either single or combined ICF components [(i) b, d; (ii) b, d, e; (iv) b, d, e, s and (v) d]. PROMs also differed widely in content coverage, ranging from instruments covering six ICF chapter to more detailed PROMs spanning up to 40–42 chapters. Sample sizes varied considerably, with bubble sizes representing populations from 8 to 895 participants. Importantly, the figure highlights the PROMs recommended according to COSMIN guidelines, with the recommendation categories (A, B, C) explicitly displayed. In this review, three PROMs were assigned to category A, all non-highlighted instruments were categorized as B, and four were classified as C.

Results of all syntheses and GRADE are reported in Supplementary material 6. No meta-analyses were feasible because of methodological heterogeneity; therefore, qualitative synthesis was undertaken.

### Certainty of evidence

Only three PROMs were classified in category A, all of which are disease-specific PROMs: HAE-QoL, NF1-AdQoL and EPP-QoL. All remaining PROMs identified in this review fall into category B, indicating potential for recommendation but requiring further research to strengthen reliability and validity. Five PROMs were classified as “C”. Flanagan’s QoL scale (QoLS), WHOQoL-BREF and SF-36 for Sickle Cell Disease and pCMT-QoL.

Detailed syntheses of reasons for downgrading and recommendations are provided in Supplementary Material 5.

### Discussion

To the best of our knowledge, this is the first systematic review specifically aimed at evaluating the MPs of PROMs assessing HRQoL in the context of rare and ultra-rare GDs.

As highlighted by *The Lancet Global Health*^*3*^, individuals and families affected by rare diseases face multifaceted challenges that extend beyond medical issues. These include significant psychosocial and economic burdens such as stigma, limited access to information, and inadequate care. The development and use of validated PROMs represent a key step toward patient-centered policy frameworks. They enable the assessment of outcomes that reflect patients’ lived experiences and support more equitable, person-centered care for individuals with rare and ultra-rare GDs.

Our comprehensive search identified 59 studies, covering a total of 45 distinct PROMs across 29 different conditions. Importantly, we did not restrict the scope to specific diagnostic categories, thereby maximizing the inclusivity of our search strategy. Therefore, the set of instruments retrieved likely represents the full range of PROMs currently available in the field of rare and ultra-rare GDs. However, when compared to the more than 7,000 rare known diseases, it becomes evident that psychometric research has been conducted in only a very small fraction of conditions, approximately 0.2% of all known rare GDs. Consistent with the observations of Slade et al. [2], this striking discrepancy highlights how most rare diseases remain underserved from the perspective of patient-reported measurement, underscoring the urgency of developing and validating PROMs that can capture the lived experience of these highly heterogeneous clinical manifestations of different patient populations.

In line with our predefined objectives, we first mapped all PROMs, evaluating HRQoL, developed and validated in the context of rare and ultra-rare GDs. Second, we examined their content through ICF linking rules, to clarify which domains and constructs they captured. Third, we critically evaluated their methodological quality to provide the basis for evidence-informed recommendations.

The findings indicate a marked imbalance in the linguistic and cultural validation of HRQoL-related PROMs in rare and ultra- rare GDs [86]. Validation studies were conducted in English and Western European languages, whereas several geographic and linguistic regions were sparsely represented. Some conditions, such as Pompe disease, hereditary angioedema, phenylketonuria, and primary ciliary dyskinesia, showed relatively broad multilingual validation, suggesting the influence of established research networks, clinical trial activity, and international collaboration [87–89]. Conversely, several instruments were available only in one or few languages, limiting their applicability in multinational studies and routine care [90]. Overall, the distribution of validated HRQoL-PROMs appears to reflect research capacity and instrument-development initiatives at least as much as disease prevalence [91].

Another relevant imbalance concerns age coverage. Although many rare and ultra-rare GDs manifest during childhood, the validation evidence identified in this review remains largely concentrated in adult or mixed-age cohorts. Pediatric and adolescent validations were available for selected conditions, including phenylketonuria, primary ciliary dyskinesia, Duchenne muscular dystrophy, Charcot–Marie–Tooth disease, low-flow malformations, hypohidrotic ectodermal dysplasia, tuberous sclerosis complex, and neurofibromatosis type 1; however, age-specific validation was not consistently available across diseases.

Importantly, the ICF-based analysis revealed substantial limitations in the conceptual coverage of HRQoL constructs. Many PROMs labelled as HRQoL measures primarily focused on body functions and functional limitations (e.g. mRPACT, EQ-5D or QLMS), with insufficient representation of broader dimensions. Three domains were consistently underrepresented: social participation, environmental factors, and psychosocial aspects such as stigma and access to care, despite their established impact on HRQoL [5, 92]. This indicates a mismatch between the multidimensional nature of HRQoL and the constructs measured by many instruments. These gaps likely reflect limited patient involvement during instrument development. Patient-centered outcome research emphasizes that valid PROMs must be grounded in patients’ lived experiences through qualitative methods and stakeholder engagement [93]. In this context, the observed deficiencies suggest that many existing PROMs were developed with insufficient adherence to patient-centered principles, resulting in an overemphasis on clinical symptoms at the expense of contextual and social dimensions [94].

Accordingly, the observed gaps can be viewed as consequences of limited adherence to methodological standards such as COSMIN [11]. More rigorous, patient-centered development processes would likely ensure inclusion of key domains such as social participation and environmental context, thereby improving the validity and decision-relevance of PROMs [95].

Another fundamental limitation emerging from this review concerns the methodological quality of validation studies. Although several disease-specific PROMs showed promising MP, the overall evidence base remains constrained by studies of limited methodological rigor, inadequate reporting, and small sample sizes, in line with previous evidence highlighting similar methodological limitations in this field [2]. A marked imbalance emerged in the evaluation of measurement properties, with internal consistency assessed in 156 scales or subscales, whereas structural validity was examined in only 27 PROMs, despite COSMIN guidance indicating that structural validity is a prerequisite for meaningful interpretation of internal consistency. This discrepancy is methodologically relevant because internal consistency assumes unidimensionality; thus, in the absence of confirmed factor structure, reliability estimates such as Cronbach’s alpha may be misleading, a limitation likely compounded by the larger sample size requirements needed to adequately assess structural validity in rare and ultra-rare GDs populations [11, 15].

Among the 45 PROMs identified, six were adapted from instruments originally designed for non–rare disease populations, whereas all the others were disease-specific. Importantly, three PROMs meeting COSMIN criteria for recommendation were disease-specific, reinforcing the importance of context-tailored measures in capturing outcomes that are meaningful to patients with rare and ultra-rare GDs. Yet, trend analyses from other studies indicate that generic PROMs are still more frequently used as primary or secondary endpoints when combined with Clinician-Reported Outcomes (ClinROs), while non–rare disease–specific instruments tend to dominate when ClinROs are used alone [96]. Consequently, when clinicians employ any of the remaining instruments classified as “not currently recommended” due to insufficient psychometric evidence, it remains uncertain whether these tools truly capture the constructs they are intended to measure. The lack of robust data on key MPs, such as content validity and internal consistency, raises legitimate concerns about the interpretability and clinical utility of the outcomes derived from these PROMs [97].

Although this systematic review makes a valuable contribution to existing literature, several limitations should be acknowledged. Key MPs such as responsiveness, measurement error, and cross-cultural measurement invariance were rarely examined in the primary studies, substantially constraining the clinical applicability and interpretability of the findings. As a result, essential indicators, including minimal important change (MIC), minimal important difference (MID), limits of agreement, and cross-language or age invariance, remain unavailable for most PROMs. Furthermore, while the ICF linking process adhered to standardized methodology, it inherently involves a degree of subjective interpretation, and minor discrepancies among reviewers may have influenced the resulting ICF mapping of specific instruments. Finally, the methodological heterogeneity and incomplete reporting of psychometric data across studies precluded quantitative synthesis. This limited the possibility of deriving pooled estimates of measurement properties and reflects the fragmented and uneven quality of the current evidence base. Future research and clinical practice should address these gaps through coordinated methodological improvements. First, the development of new PROMs should be prioritized for underrepresented rare conditions, adopting scalable and cross-disease approaches [98]. Second, cross-cultural and multilingual validation must be systematically integrated to ensure broader applicability and measurement invariance. Third, age-specific validation, particularly in pediatric populations, should be consistently implemented. Fourth, PROM development should adhere to patient-centered methodologies, incorporating qualitative research and stakeholder engagement to ensure adequate coverage of social, environmental, and psychosocial domains. Fifth, stronger adherence to methodological standards (e.g., COSMIN) is required, with priority given to content and structural validity before reliability assessment. Sixth, validation studies should expand to underreported properties, including responsiveness, measurement error, and interpretability (e.g., MIC, MID). Finally, clinicians should preferentially use PROMs supported by robust psychometric evidence and contribute to evidence generation when using insufficiently validated instruments, thereby strengthening the cumulative evidence base.

### Conclusions

This systematic review provides the first comprehensive synthesis of PROMs specifically developed or validated to assess HRQoL in rare and ultra-rare genetic diseases, integrating psychometric evidence with an ICF-based conceptual framework. Despite growing attention to the patient’s perspective, the available evidence remains fragmented and methodologically heterogeneous. Only three PROMs met the COSMIN criteria for recommendation, [11] all of which were disease-specific, underscoring the importance of context-tailored measurement to capture outcomes that are truly meaningful to patients. However, most identified instruments still lack sufficient evidence for core measurement properties such as content validity, structural validity and, consequently, internal consistency, raising uncertainty as to whether they adequately capture the multidimensional construct of QoL. In this context, ensuring a rigorous content validity process, grounded in the involvement of both patients and relevant stakeholders, is essential to identify which HRQoL domains are most relevant within specific disease contexts. Multicenter and interdisciplinary collaborations that actively involve patient organizations, clinicians, and researchers will be essential to overcoming sample size limitations and improving conceptual consistency. Although some gaps remain in the coverage of specific ICF domains, these appear secondary to the broader need for well-constructed, disease-specific instruments capable of capturing the complexity of QoL. Ultimately, advancing robust, disease-relevant PROMs will not only enhance the methodological quality of rare disease research but also strengthen patient-centered care and equity in outcome assessment.

## Electronic supplementary material

Below is the link to the electronic supplementary material.

Supplementary Material 1

Supplementary Material 2

Supplementary Material 3

Supplementary Material 4

Supplementary Material 5

Supplementary Material 6

## Data Availability

All data generated or analysed during this study are included in this published article and its supplementary information files.

## Appendix

Supporting information and data associated with this article have been uploaded as supplementary material

## Abbreviations

−: Insufficient
?: Indeterminate
+: Sufficient
±: Inconsistent
AE-QoL: Angioedema Quality of Life Questionnaire
AFQOL: Adult Fabry Disease Quality of Life
CMTHI: Charcot–Marie–Tooth Health Index
COSMIN: Consensus-based Standards for Selection of Health Measurement Instruments
cLFM-QoL: Children Low Flow Malformations Quality of Life
cTTP-PEQ: Congenital Thrombotic Thrombocytopenic Purpura Patient Experience Questionnaire
EPP-QoL: Erythropoietic Protoporphyria Quality of Life
EQ-5D: EuroQol Five-Dimension Questionnaire
GD: Genetic disease
H-QoL-I: Huntington Quality of Life Instrument
HD-PRO-TRIAD: Huntington Disease Patient-Reported Outcome TRIAD
HDQLIFE: Huntington Disease Quality of Life
ICF: International Classification of Functioning, Disability and Health
IRDiRC: International Rare Diseases Research Consortium
MPs: Measurement Properties
NF1-AdQoL: Neurofibromatosis Type 1 Adult Health-Related Quality of Life
Norfolk QoL-DN: Norfolk Quality of Life–Diabetic Neuropathy
pCMT-QoL: Pediatric Charcot–Marie–Tooth Quality of Life
PFDD: Patient-Focused Drug Development
PKU-QOL: Phenylketonuria Quality of Life
Pompe QoL: Pompe Disease Quality of Life
PRAIS: Person-Reported Ataxia Impact Scale
QLQ-AA/PNH: Quality of Life Questionnaire for Aplastic Anemia/Paroxysmal Nocturnal Hemoglobinuria
HRQoL: Health Related Quality of life
QoL-HHT: Quality of Life in Hereditary Hemorrhagic Telangiectasia
QoL-PROMs: Quality-of-life patient-reported outcome measures
QoLS: Flanagan’s Quality of Life Scale
QOL-PCD: Quality of Life–Primary Ciliary Dyskinesia
QOLIE-31-P: Quality of Life in Epilepsy Inventory-31 Problem
QOLIE-AD-48: Quality of Life in Epilepsy Inventory for Adolescents
R-PAct: Rasch-Built Pompe-Specific Activity Scale
RoB: Risk of Bias
SF-36: 36-item Short Form Health Survey
VISIO-PRO: Visual Symptom and Impact Outcomes Patient-Reported Outcome
WHO: World Health Organization
WHOQOL-BREF: World Health Organization Quality of Life – BREF
