# Supplementary material for: Self-reported perspective in rare genetic diseases: a systematic review of patient-reported outcome measures classified using the international classification of functioning, disability and health framework
Source: J Patient Rep Outcomes. 2026 Jul 25;10:141. doi: 10.1186/s41687-026-01155-5 (PMC13462012; doi:10.1186/s41687-026-01155-5)
Supplement: Supplementary file 1 [file 41687_2026_1155_MOESM1_ESM.docx]

**26/03/2026**

("Quality of Life"[Mesh] OR "quality of life"[tiab] OR life qualit*[tiab] OR living qualit*[tiab] OR "quality of living"[tiab] OR "Activities of Daily Living"[Mesh] OR "activities of daily living"[tiab] OR "activity of daily living"[tiab] OR "activities of daily life"[tiab] OR "activity of daily life"[tiab] OR daily living activit*[tiab] OR daily life activit*[tiab] OR "adl"[tiab] OR "chronic limitation of activity"[tiab] OR self care*[tiab] OR "Health Status"[Mesh] OR "health status"[tiab] OR "level of health"[tiab] OR health level*[tiab] OR "qol"[tiab] OR "hrql"[tiab] OR "hrqol"[tiab]) AND ((("rare disease*" OR "rare disorder*" OR "genetic disorder*" OR "ultra-rare disease*" OR "ultra-rare disorder*" OR "genetic disease*") AND ("Patient Reported Outcome Measures"[Mesh] OR "Quality of Life"[Mesh] OR prom[tiab] OR proms[tiab] OR pro[tiab] OR pros[tiab] OR HRQL[tiab] OR HRQoL[tiab] OR QL[tiab] OR QoL[tiab] OR quality of life[tiab] OR life quality[tiab] OR health index*[tiab] OR health indices[tiab] OR health profile*[tiab] OR health status[tw] OR ((patient[tiab] OR self[tiab] OR child[tiab] OR parent[tiab] OR carer[tiab] OR proxy[tiab]) AND ((report[tiab] OR reported[tiab] OR reporting[tiab]) OR (rated[tiab] OR rating[tiab] OR ratings[tiab]) OR based[tiab] OR (assessed[tiab] OR assessment[tiab] OR assessments[tiab]))) OR ((disability[tiab] OR function[tiab] OR functional[tiab] OR functions[tiab] OR subjective[tiab] OR utility[tiab] OR utilities[tiab] OR wellbeing[tiab] OR well being[tiab]) AND (outcome[tiab] OR outcomes[tiab] OR index[tiab] OR indices[tiab] OR instrument[tiab] OR instruments[tiab] OR measure[tiab] OR measures[tiab] OR questionnaire[tiab] OR questionnaires[tiab] OR profile[tiab] OR profiles[tiab] OR scale[tiab] OR scales[tiab] OR score[tiab] OR scores[tiab] OR status[tiab] OR survey[tiab] OR surveys[tiab])))) AND (((instrumentation[sh] OR "Validation Studies"[pt] OR "reproducibility of results"[MeSH Terms] OR reproducib*[tiab] OR "psychometrics"[MeSH] OR psychometr*[tiab] OR clinimetr*[tiab] OR clinometr*[tiab] OR "observer variation"[MeSH] OR "observer variation"[tiab] OR "discriminant analysis"[MeSH] OR reliab*[tiab] OR valid*[tiab] OR coefficient[tiab] OR "internal consistency"[tiab] OR (cronbach*[tiab] AND (alpha[tiab] OR alphas[tiab])) OR "item correlation"[tiab] OR "item correlations"[tiab] OR "item selection"[tiab] OR "item selections"[tiab] OR "item reduction"[tiab] OR "item reductions"[tiab] OR agreement[tiab] OR precision[tiab] OR imprecision[tiab] OR "precise values"[tiab] OR test-retest[tiab] OR (test[tiab] AND retest[tiab]) OR (reliab*[tiab] AND (test[tiab] OR retest[tiab])) OR stability[tiab] OR interrater[tiab] OR interrater[tiab] OR intrarater[tiab] OR intra-rater[tiab] OR intertester[tiab] OR inter-tester[tiab] OR intratester[tiab] OR intra-tester[tiab] OR interobserver[tiab] OR inter-observer[tiab] OR intraobserver[tiab] OR intra-observer[tiab] OR intertechnician[tiab] OR inter-technician[tiab] OR intratechnician[tiab] OR intra-technician[tiab] OR interexaminer[tiab] OR inter-examiner[tiab] OR intraexaminer[tiab] OR intra-examiner[tiab] OR interassay[tiab] OR inter-assay[tiab] OR intraassay[tiab] OR intraassay[tiab] OR interindividual[tiab] OR inter-individual[tiab] OR intraindividual[tiab] OR intra-individual[tiab] OR interparticipant[tiab] OR inter-participant[tiab] OR intraparticipant[tiab] OR intra-participant[tiab] OR kappa[tiab] OR kappa's[tiab] OR kappas[tiab] OR repeatab*[tiab] OR ((replicab*[tiab] OR repeated[tiab]) AND (measure[tiab] OR measures[tiab] OR findings[tiab] OR result[tiab] OR results[tiab] OR test[tiab] OR tests[tiab])) OR generaliza*[tiab] OR generalisa*[tiab] OR concordance[tiab] OR (intraclass[tiab] AND correlation*[tiab]) OR discriminative[tiab] OR "known group"[tiab] OR "factor analysis"[tiab] OR "factor analyses"[tiab] OR dimensionality[tiab] OR subscale*[tiab] OR "multitrait scaling analysis"[tiab] OR "multitrait scaling analyses"[tiab] OR "item discriminant"[tiab]OR "interscale correlation"[tiab] OR "interscale correlations"[tiab] OR ((error[tiab] OR errors[tiab]) AND (measure*[tiab] OR correlat*[tiab] OR evaluat*[tiab] OR accuracy[tiab] OR accurate[tiab] OR precision[tiab] OR mean[tiab])) OR "individual variability"[tiab] OR "variability analysis"[tiab] OR (uncertainty[tiab] AND (measurement[tiab] OR measuring[tiab])) OR "standard error of measurement"[tiab] OR sensitiv*[tiab] OR responsive*[tiab] OR (small*[tiab] AND (real[tiab] OR detectable[tiab]) AND (change[tiab] OR difference[tiab])) OR "meaningful change"[tiab] OR "minimal important change"[tiab] OR "minimal important difference"[tiab] OR "minimally important change"[tiab] OR "minimally important difference"[tiab] OR "minimal detectable change"[tiab] OR "minimal detectable difference"[tiab] OR "minimally detectable change"[tiab] OR "minimally detectable difference"[tiab] OR "minimal real change"[tiab] OR "minimal real difference"[tiab] OR "minimally real change"[tiab] OR "minimally real difference"[tiab] OR "ceiling effect"[tiab] OR "floor effect"[tiab] OR "item response model"[tiab] OR irt[tiab] OR rasch[tiab] OR "differential item functioning"[tiab] OR dif[tiab] OR "computer adaptive testing"[tiab] OR "item bank"[tiab] OR "cross-cultural equivalence"[tiab])))

**Pubmed** = 1128

'quality of life'/exp OR ((life NEXT/1 qualit*):ab,ti) OR 'quality of life':ab,ti OR 'daily life activity'/exp OR 'activities of daily living':ab,ti OR (('daily living' NEXT/1 activit*):ab,ti) OR (('daily live' NEXT/1 activit*):ab,ti) OR 'adl':ab,ti OR 'chronic limitation of activity':ab,ti OR ((self NEXT/1 care*):ab,ti) OR 'health status'/exp OR 'health status':ab,ti OR 'level of health':ab,ti OR ((health NEXT/1 level*):ab,ti) OR 'qol':ab,ti OR 'hrql':ab,ti OR 'hrqol':ab,ti AND 'instrumentation'/exp OR 'instrumentation' OR 'validation studies':it OR 'reproducibility of results'/exp OR 'reproducibility of results' OR reproducib*:ab,ti OR 'psychometrics'/exp OR psychometrics OR psychometr*:ab,ti OR clinimetr*:ab,ti OR clinometr*:ab,ti OR 'observer variation'/exp OR 'observer variation' OR 'observer variation':ab,ti OR 'discriminant analysis'/exp OR 'discriminant analysis' OR reliab*:ab,ti OR valid*:ab,ti OR coefficient:ab,ti OR 'internal cosistency':ab,ti OR (cronbach*:ab,ti AND (alpha:ab,ti OR alphas:ab,ti)) OR 'item correlation':ab,ti OR 'item correlations':ab,ti OR 'item selection':ab,ti OR 'item selections':ab,ti OR 'item reduction':ab,ti OR 'item reductions':ab,ti OR agreement:ab,ti OR precision:ab,ti OR imprecision:ab,ti OR 'precise values':ab,ti OR 'test retest':ab,ti OR (test:ab,ti AND retest:ab,ti) OR (reliab*:ab,ti AND (test:ab,ti OR retest:ab,ti)) OR stability:ab,ti OR interrater:ab,ti OR intrarater:ab,ti OR 'intra rater':ab,ti OR intertester:ab,ti OR 'inter tester':ab,ti OR intratester:ab,ti OR 'intra tester':ab,ti OR interobserver:ab,ti OR 'inter observer':ab,ti OR intraobserver:ab,ti OR 'intra observer':ab,ti OR intertechnician:ab,ti OR 'inter technician':ab,ti OR intratechnician:ab,ti OR 'intra technician':ab,ti OR interexaminer:ab,ti OR 'inter examiner':ab,ti OR intraexaminer:ab,ti OR 'intra examiner':ab,ti OR interassay:ab,ti OR 'inter assay':ab,ti OR intraassay:ab,ti OR interindividual:ab,ti OR 'inter individual':ab,ti OR intraindividual:ab,ti OR 'intra individual':ab,ti OR interparticipant:ab,ti OR 'inter participant':ab,ti OR intraparticipant:ab,ti OR 'intra participant':ab,ti OR kappa:ab,ti OR kappas:ab,ti OR repeatab*:ab,ti OR ((replicab*:ab,ti OR repeated:ab,ti) AND (measure:ab,ti OR measures:ab,ti OR findings:ab,ti OR result:ab,ti OR results:ab,ti OR test:ab,ti OR tests:ab,ti)) OR generaliza*:ab,ti OR generalisa*:ab,ti OR concordance:ab,ti OR (intraclass:ab,ti AND correlation*:ab,ti) OR discriminative:ab,ti OR 'known group':ab,ti OR 'factor analysis':ab,ti OR 'factor analyses':ab,ti OR dimensionality:ab,ti OR subscale*:ab,ti OR 'multitrait scaling analysis':ab,ti OR 'multitrait scaling analyses':ab,ti OR 'item discriminant' OR 'interscale correlation':ab,ti OR 'interscale correlations':ab,ti OR ((error:ab,ti OR errors:ab,ti) AND (measure*:ab,ti OR correlat*:ab,ti OR evaluat*:ab,ti OR accuracy:ab,ti OR accurate:ab,ti OR precision:ab,ti OR mean:ab,ti)) OR 'individual variability':ab,ti OR 'variability analysis':ab,ti OR (uncertainty:ab,ti AND (measurement:ab,ti OR measuring:ab,ti)) OR 'standard error of measurement':ab,ti OR sensitiv*:ab,ti OR responsive*:ab,ti OR (small*:ab,ti AND (real:ab,ti OR detectable:ab,ti) AND (change:ab,ti OR difference:ab,ti)) OR 'meaningful change':ab,ti OR 'minimal important change':ab,ti OR 'minimal important difference':ab,ti OR 'minimally important change':ab,ti OR 'minimally important difference':ab,ti OR 'minimal detectable change':ab,ti OR 'minimal detectable difference':ab,ti OR 'minimally detectable change':ab,ti OR 'minimally detectable difference':ab,ti OR 'minimal real change':ab,ti OR 'minimal real difference':ab,ti OR 'minimally real change':ab,ti OR 'minimally real difference':ab,ti OR 'ceiling effect':ab,ti OR 'floor effect':ab,ti OR 'item response model':ab,ti OR irt:ab,ti OR rasch:ab,ti OR 'differential item functioning':ab,ti OR dif:ab,ti OR 'computer adaptive testing':ab,ti OR 'item bank':ab,ti OR 'cross-cultural equivalence':ab,ti AND 'rare disease*' OR 'rare disorder*' OR 'genetic disorder*' OR 'ultra-rare disease*' OR 'ultra-rare disorder*' OR 'genetic disease*'AND (('patient reported outcome measures'/exp OR 'quality of life'/exp OR prom:ti,ab OR proms:ti,ab OR pro:ti,ab OR pros:ti,ab OR hrql:ti,ab OR hrqol:ti,ab OR ql:ti,ab OR qol:ti,ab OR 'quality of life':ti,ab OR 'life quality':ti,ab OR 'health index*':ti,ab OR 'health indices':ti,ab OR 'health profile*':ti,ab OR 'health status' OR patient:ti,ab OR self:ti,ab OR child:ti,ab OR parent:ti,ab OR carer:ti,ab OR proxy:ti,ab) AND report:ti,ab OR reported:ti,ab OR reporting:ti,ab OR rated:ti,ab OR rating:ti,ab OR ratings:ti,ab OR based:ti,ab OR assessed:ti,ab OR assessment:ti,ab OR assessments:ti,ab OR disability:ti,ab OR function:ti,ab OR functional:ti,ab OR functions:ti,ab OR subjective:ti,ab OR utility:ti,ab OR utilities:ti,ab OR wellbeing:ti,ab OR 'well being':ti,ab) AND outcome:ti,ab OR outcomes:ti,ab OR index:ti,ab OR indices:ti,ab OR instrument:ti,ab OR instruments:ti,ab OR measure:ti,ab OR measures:ti,ab OR questionnaire:ti,ab OR questionnaires:ti,ab OR profile:ti,ab OR profiles:ti,ab OR scale:ti,ab OR scales:ti,ab OR score:ti,ab OR scores:ti,ab OR status:ti,ab OR survey:ti,ab OR surveys:ti,ab

**EMBASE = 3184**

(MH "Quality of Life+") OR (TI "quality of life" OR AB "quality of life") OR (TI "life qualit*" OR AB "life qualit*") OR (TI "living qualit*" OR AB "living qualit*") OR (TI "quality of living" OR AB "quality of living") OR (MH "Activities of Daily Living+") OR (TI "activities of daily living" OR AB "activities of daily living") OR (TI "activity of daily living" OR AB "activity of daily living") OR (TI "activities of daily life" OR AB "activities of daily life") OR (TI "activity of daily life" OR AB "activity of daily life") OR (TI "daily living activit*" OR AB "daily living activit*") OR (TI "daily life activit*" OR AB "daily life activit*") OR (TI adl OR AB adl) OR (TI "chronic limitation of activity" OR AB "chronic limitation of activity") OR (TI "self care*" OR AB "self care*") OR (MH "Health Status+") OR (TI "health status" OR AB "health status") OR (TI "level of health" OR AB "level of health") OR (TI "health level*" OR AB "health level*") OR (TI qol OR AB qol) OR (TI hrql OR AB hrql) OR (TI hrqol OR AB hrqol)) AND (MH "Patient Reported Outcome Measures+") OR (MH "Quality of Life+") OR (TI prom OR AB prom) OR (TI proms OR AB proms) OR (TI pro OR AB pro) OR (TI pros OR AB pros) OR (TI HRQL OR AB HRQL) OR (TI HRQoL OR AB HRQoL) OR (TI QL OR AB QL) OR (TI QoL OR AB QoL) OR (TI "quality of life" OR AB "quality of life") OR (TI "life quality" OR AB "life quality") OR (TI "health index*" OR AB "health index*") OR (TI "health indices" OR AB "health indices") OR (TI "health profile*" OR AB "health profile*") OR "health status" OR (((TI patient OR AB patient) OR (TI self OR AB self) OR (TI child OR AB child) OR (TI parent OR AB parent) OR (TI carer OR AB carer) OR (TI proxy OR AB proxy)) AND (((TI report OR AB report) OR (TI reported OR AB reported) OR (TI reporting OR AB reporting)) OR ((TI rated OR AB rated) OR (TI rating OR AB rating) OR (TI ratings OR AB ratings)) OR (TI based OR AB based) OR ((TI assessed OR AB assessed) OR (TI assessment OR AB assessment) OR (TI assessments OR AB assessments)))) OR (((TI disability OR AB disability) OR (TI function OR AB function) OR (TI functional OR AB functional) OR (TI functions OR AB functions) OR (TI subjective OR AB subjective) OR (TI utility OR AB utility) OR (TI utilities OR AB utilities) OR (TI wellbeing OR AB wellbeing) OR (TI "well being" OR AB "well being")) AND ((TI outcome OR AB outcome) OR (TI outcomes OR AB outcomes) OR (TI index OR AB index) OR (TI indices OR AB indices) OR (TI instrument OR AB instrument) OR (TI instruments OR AB instruments) OR (TI measure OR AB measure) OR (TI measures OR AB measures) OR (TI questionnaire OR AB questionnaire) OR (TI questionnaires OR AB questionnaires) OR (TI profile OR AB profile) OR (TI profiles OR AB profiles) OR (TI scale OR AB scale) OR (TI scales OR AB scales) OR (TI score OR AB score) OR (TI scores OR AB scores) OR (TI status OR AB status) OR (TI survey OR AB survey) OR (TI surveys OR AB surveys))) AND (("Instrumentation" OR (PT "Validation Studies") OR (MH "reproducibility of results+") OR (TI reproducib* OR AB reproducib*) OR (MH psychometrics+) OR (TI psychometr* OR AB psychometr*) OR (TI clinimetr* OR AB clinimetr*) OR (TI clinometr* OR AB clinometr*) OR (MH "observer variation+") OR (TI "observer variation" OR AB "observer variation") OR (MH "discriminant analysis+") OR (TI reliab* OR AB reliab*) OR (TI valid* OR AB valid*) OR (TI coefficient OR AB coefficient) OR (TI "internal consistency" OR AB "internal consistency") OR ((TI cronbach* OR AB cronbach*) AND ((TI alpha OR AB alpha) OR (TI alphas OR AB alphas))) OR (TI "item correlation" OR AB "item correlation") OR (TI "item correlations" OR AB "item correlations") OR (TI "item selection" OR AB "item selection") OR (TI "item selections" OR AB "item selections") OR (TI "item reduction" OR AB "item reduction") OR (TI "item reductions" OR AB "item reductions") OR (TI agreement OR AB agreement) OR (TI precision OR AB precision) OR (TI imprecision OR AB imprecision) OR (TI "precise values" OR AB "precise values") OR (TI test-retest OR AB test-retest) OR ((TI test OR AB test) AND (TI retest OR AB retest)) OR ((TI reliab* OR AB reliab*) AND ((TI test OR AB test) OR (TI retest OR AB retest))) OR (TI stability OR AB stability) OR (TI interrater OR AB interrater) OR (TI interrater OR AB interrater) OR (TI intrarater OR AB intrarater) OR (TI intra-rater OR AB intra-rater) OR (TI intertester OR AB intertester) OR (TI inter-tester OR AB inter-tester) OR (TI intratester OR AB intratester) OR (TI intra-tester OR AB intra-tester) OR (TI interobserver OR AB interobserver) OR (TI inter-observer OR AB inter-observer) OR (TI intraobserver OR AB intraobserver) OR (TI intra-observer OR AB intra-observer) OR (TI intertechnician OR AB intertechnician) OR (TI inter-technician OR AB inter-technician) OR (TI intratechnician OR AB intratechnician) OR (TI intra-technician OR AB intra-technician) OR (TI interexaminer OR AB interexaminer) OR (TI inter-examiner OR AB inter-examiner) OR (TI intraexaminer OR AB intraexaminer) OR (TI intra-examiner OR AB intra-examiner) OR (TI interassay OR AB interassay) OR (TI inter-assay OR AB inter-assay) OR (TI intraassay OR AB intraassay) OR (TI intraassay OR AB intraassay) OR (TI interindividual OR AB interindividual) OR (TI inter-individual OR AB inter-individual) OR (TI intraindividual OR AB intraindividual) OR (TI intra-individual OR AB intra-individual) OR (TI interparticipant OR AB interparticipant) OR (TI inter-participant OR AB inter-participant) OR (TI intraparticipant OR AB intraparticipant) OR (TI intra-participant OR AB intra-participant) OR (TI kappa OR AB kappa) OR (TI kappa's OR AB kappa's) OR (TI kappas OR AB kappas) OR (TI repeatab* OR AB repeatab*) OR (((TI replicab* OR AB replicab*) OR (TI repeated OR AB repeated)) AND ((TI measure OR AB measure) OR (TI measures OR AB measures) OR (TI findings OR AB findings) OR (TI result OR AB result) OR (TI results OR AB results) OR (TI test OR AB test) OR (TI tests OR AB tests))) OR (TI generaliza* OR AB generaliza*) OR (TI generalisa* OR AB generalisa*) OR (TI concordance OR AB concordance) OR ((TI intraclass OR AB intraclass) AND (TI correlation* OR AB correlation*)) OR (TI discriminative OR AB discriminative) OR (TI "known group" OR AB "known group") OR (TI "factor analysis" OR AB "factor analysis") OR (TI "factor analyses" OR AB "factor analyses") OR (TI dimensionality OR AB dimensionality) OR (TI subscale* OR AB subscale*) OR (TI "multitrait scaling analysis" OR AB "multitrait scaling analysis") OR (TI "multitrait scaling analyses" OR AB "multitrait scaling analyses") OR (TI "item discriminantOR "interscale correlation"" OR AB "item discriminantOR "interscale correlation"") OR (TI "interscale correlations" OR AB "interscale correlations") OR (((TI error OR AB error) OR (TI errors OR AB errors)) AND ((TI measure* OR AB measure*) OR (TI correlat* OR AB correlat*) OR (TI evaluat* OR AB evaluat*) OR (TI accuracy OR AB accuracy) OR (TI accurate OR AB accurate) OR (TI precision OR AB precision) OR (TI mean OR AB mean))) OR (TI "individual variability" OR AB "individual variability") OR (TI "variability analysis" OR AB "variability analysis") OR ((TI uncertainty OR AB uncertainty) AND ((TI measurement OR AB measurement) OR (TI measuring OR AB measuring))) OR (TI "standard error of measurement" OR AB "standard error of measurement") OR (TI sensitiv* OR AB sensitiv*) OR (TI responsive* OR AB responsive*) OR ((TI small* OR AB small*) AND ((TI real OR AB real) OR (TI detectable OR AB detectable)) AND ((TI change OR AB change) OR (TI difference OR AB difference))) OR (TI "meaningful change" OR AB "meaningful change") OR (TI "minimal important change" OR AB "minimal important change") OR (TI "minimal important difference" OR AB "minimal important difference") OR (TI "minimally important change" OR AB "minimally important change") OR (TI "minimally important difference" OR AB "minimally important difference") OR (TI "minimal detectable change" OR AB "minimal detectable change") OR (TI "minimal detectable difference" OR AB "minimal detectable difference") OR (TI "minimally detectable change" OR AB "minimally detectable change") OR (TI "minimally detectable difference" OR AB "minimally detectable difference") OR (TI "minimal real change" OR AB "minimal real change") OR (TI "minimal real difference" OR AB "minimal real difference") OR (TI "minimally real change" OR AB "minimally real change") OR (TI "minimally real difference" OR AB "minimally real difference") OR (TI "ceiling effect" OR AB "ceiling effect") OR (TI "floor effect" OR AB "floor effect") OR (TI "item response model" OR AB "item response model") OR (TI irt OR AB irt) OR (TI rasch OR AB rasch) OR (TI "differential item functioning" OR AB "differential item functioning") OR (TI dif OR AB dif) OR (TI "computer adaptive testing" OR AB "computer adaptive testing") OR (TI "item bank" OR AB "item bank") OR (TI "cross-cultural equivalence" OR AB "cross-cultural equivalence")) AND "rare disease*" OR "rare disorder*" OR "genetic disorder*" OR "ultra-rare disease*" OR "ultra-rare disorder*" OR "genetic disease*"

**Psychinfo = 106**

( TS="rare disease*" OR TS="rare disorder*" OR TS="genetic disorder*" OR TS="ultra-rare disease*" OR TS="ultra-rare disorder*" OR TS="genetic disease*") AND ( TS="Quality of Life" OR TS="Activities of Daily Living" OR TS="Health Status" OR (TI="quality of life" OR AB="quality of life") OR (TI="life qualit*" OR AB="life qualit*") OR (TI="living qualit*" OR AB="living qualit*") OR (TI="quality of living" OR AB="quality of living") OR (TI="activities of daily living" OR AB="activities of daily living") OR (TI="activity of daily living" OR AB="activity of daily living") OR (TI="activities of daily life" OR AB="activities of daily life") OR (TI="activity of daily life" OR AB="activity of daily life") OR (TI="daily living activit*" OR AB="daily living activit*") OR (TI="daily life activit*" OR AB="daily life activit*") OR (TI=adl OR AB=adl) OR (TI="chronic limitation of activity" OR AB="chronic limitation of activity") OR (TI="self care*" OR AB="self care*") OR (TI="health status" OR AB="health status") OR (TI="level of health" OR AB="level of health") OR (TI="health level*" OR AB="health level*") OR (TI=qol OR AB=qol) OR (TI=hrql OR AB=hrql) OR (TI=hrqol OR AB=hrqol)) AND (TS="Patient Reported Outcome Measures" OR TS="Quality of Life" OR TS="health status"OR (TI=prom OR AB=prom) OR (TI=proms OR AB=proms) OR (TI=pro OR AB=pro) OR (TI=pros OR AB=pros) OR (TI=HRQL OR AB=HRQL) OR (TI=HRQoL OR AB=HRQoL) OR (TI=QL OR AB=QL) OR (TI=QoL OR AB=QoL) OR (TI="quality of life" OR AB="quality of life") OR (TI="life quality" OR AB="life quality") OR (TI="health index*" OR AB="health index*") OR (TI="health indices" OR AB="health indices") OR (TI="health profile*" OR AB="health profile*") OR ( (TI=patient OR AB=patient OR TI=self OR AB=self OR TI=child OR AB=child OR TI=parent OR AB=parent OR TI=carer OR AB=carer OR TI=proxy OR AB=proxy) AND ((TI=report OR AB=report OR TI=reported OR AB=reported OR TI=reporting OR AB=reporting) OR (TI=rated OR AB=rated OR TI=rating OR AB=rating OR TI=ratings OR AB=ratings) OR (TI=based OR AB=based) OR (TI=assessed OR AB=assessed OR TI=assessment OR AB=assessment OR TI=assessments OR AB=assessments))) OR ( (TI=disability OR AB=disability OR TI=function OR AB=function OR TI=functional OR AB=functional OR TI=functions OR AB=functions OR TI=subjective OR AB=subjective OR TI=utility OR AB=utility OR TI=utilities OR AB=utilities OR TI=wellbeing OR AB=wellbeing OR TI="well being" OR AB="well being") AND (TI=outcome OR AB=outcome OR TI=outcomes OR AB=outcomes OR TI=index OR AB=index OR TI=indices OR AB=indices OR TI=instrument OR AB=instrument OR TI=instruments OR AB=instruments OR TI=measure OR AB=measure OR TI=measures OR AB=measures OR TI=questionnaire OR AB=questionnaire OR TI=questionnaires OR AB=questionnaires OR TI=profile OR AB=profile OR TI=profiles OR AB=profiles OR TI=scale OR AB=scale OR TI=scales OR AB=scales OR TI=score OR AB=score OR TI=scores OR AB=scores OR TI=status OR AB=status OR TI=survey OR AB=survey OR TI=surveys OR AB=surveys))) AND (TS=("Instrumentation" OR "Validation Studies" OR "reproducibility of results" OR "observer variation" OR "discriminant analysis" OR psychometrics) OR (TI=reproducib* OR AB=reproducib*) OR (TI=psychometr* OR AB=psychometr*) OR (TI=clinimetr* OR AB=clinimetr*) OR (TI=clinometr* OR AB=clinometr*) OR (TI=reliab* OR AB=reliab*) OR (TI=valid* OR AB=valid*) OR (TI=coefficient OR AB=coefficient) OR (TI="internal consistency" OR AB="internal consistency") OR ((TI=cronbach* OR AB=cronbach*) AND (TI=alpha OR AB=alpha OR TI=alphas OR AB=alphas)) OR (TI="item correlation" OR AB="item correlation") OR (TI="item correlations" OR AB="item correlations") OR (TI="item selection" OR AB="item selection") OR (TI="item selections" OR AB="item selections") OR (TI="item reduction" OR AB="item reduction") OR (TI="item reductions" OR AB="item reductions") OR (TI=agreement OR AB=agreement) OR (TI=precision OR AB=precision) OR (TI=imprecision OR AB=imprecision) OR (TI="precise values" OR AB="precise values") OR (TI=test-retest OR AB=test-retest) OR ((TI=test OR AB=test) AND (TI=retest OR AB=retest)) OR ((TI=reliab* OR AB=reliab*) AND (TI=test OR AB=test OR TI=retest OR AB=retest)) OR (TI=stability OR AB=stability) OR (TI=interrater OR AB=interrater) OR (TI=intrarater OR AB=intrarater OR TI=intra-rater OR AB=intra-rater) OR (TI=intertester OR AB=intertester OR TI=inter-tester OR AB=inter-tester) OR (TI=intratester OR AB=intratester OR TI=intra-tester OR AB=intra-tester) OR (TI=interobserver OR AB=interobserver OR TI=inter-observer OR AB=inter-observer) OR (TI=intraobserver OR AB=intraobserver OR TI=intra-observer OR AB=intra-observer) OR (TI=kappa OR AB=kappa OR TI=kappas OR AB=kappas OR TI="kappa's" OR AB="kappa's") OR (TI=repeatab* OR AB=repeatab*) OR ((TI=replicab* OR AB=replicab* OR TI=repeated OR AB=repeated) AND (TI=measure OR AB=measure OR TI=measures OR AB=measures OR TI=result* OR AB=result* OR TI=test* OR AB=test*)) OR (TI=generaliza* OR AB=generaliza* OR TI=generalisa* OR AB=generalisa*) OR (TI=concordance OR AB=concordance) OR ((TI=intraclass OR AB=intraclass) AND (TI=correlation* OR AB=correlation*)) OR (TI=discriminative OR AB=discriminative) OR (TI="known group" OR AB="known group") OR (TI="factor analysis" OR AB="factor analysis") OR (TI="factor analyses" OR AB="factor analyses") OR (TI=dimensionality OR AB=dimensionality) OR (TI=subscale* OR AB=subscale*) OR (TI="multitrait scaling analysis" OR AB="multitrait scaling analysis") OR (TI="multitrait scaling analyses" OR AB="multitrait scaling analyses") OR (TI="item discriminant" OR AB="item discriminant" OR TI="interscale correlation" OR AB="interscale correlation") OR (TI="interscale correlations" OR AB="interscale correlations") OR ((TI=error OR AB=error OR TI=errors OR AB=errors) AND (TI=measure* OR AB=measure* OR TI=correlat* OR AB=correlat* OR TI=evaluat* OR AB=evaluat* OR TI=accuracy OR AB=accuracy OR TI=precision OR AB=precision)) OR (TI="individual variability" OR AB="individual variability") OR (TI="variability analysis" OR AB="variability analysis") OR ((TI=uncertainty OR AB=uncertainty) AND (TI=measurement OR AB=measurement OR TI=measuring OR AB=measuring)) OR (TI="standard error of measurement" OR AB="standard error of measurement") OR (TI=sensitiv* OR AB=sensitiv*) OR (TI=responsive* OR AB=responsive*) OR ((TI=small* OR AB=small*) AND (TI=real OR AB=real OR TI=detectable OR AB=detectable) AND (TI=change OR AB=change OR TI=difference OR AB=difference)) OR (TI="meaningful change" OR AB="meaningful change") OR (TI="minimal important change" OR AB="minimal important change") OR (TI="minimal important difference" OR AB="minimal important difference") OR (TI="minimally important change" OR AB="minimally important change") OR (TI="minimally important difference" OR AB="minimally important difference") OR (TI="minimal detectable change" OR AB="minimal detectable change") OR (TI="minimal detectable difference" OR AB="minimal detectable difference") OR (TI="ceiling effect" OR AB="ceiling effect") OR (TI="floor effect" OR AB="floor effect") OR (TI="item response model" OR AB="item response model") OR (TI=irt OR AB=irt) OR (TI=rasch OR AB=rasch) OR (TI="differential item functioning" OR AB="differential item functioning") OR (TI=dif OR AB=dif) OR (TI="computer adaptive testing" OR AB="computer adaptive testing") OR (TI="item bank" OR AB="item bank") OR (TI="cross-cultural equivalence" OR AB="cross-cultural equivalence"))

**WoS = 796**
