# Supplementary material for: Self-reported perspective in rare genetic diseases: a systematic review of patient-reported outcome measures classified using the international classification of functioning, disability and health framework
Source: J Patient Rep Outcomes. 2026 Jul 25;10:141. doi: 10.1186/s41687-026-01155-5 (PMC13462012; doi:10.1186/s41687-026-01155-5)
Supplement: Supplementary file 2 [file 41687_2026_1155_MOESM2_ESM.docx]

| **ID** | **title** | **year** | **journal** | **authors** | **abstract** | **Exclusion reasons** |
| --- | --- | --- | --- | --- | --- | --- |
| 1 | Bridging the clinical-research gap: Harnessing an electronic data capture, integration, and visualization platform to systematically assess prospective patient-reported outcomes in mitochondrial medicine | 2024 | MOLECULAR GENETICS AND METABOLISM | MacMullen, LE and George-Sankoh, I and Stanley, K and McCormick, EM and Muraresku, CC and Goldstein, A and Zolkipli-Cunningham, Z and Falk, MJ | Purpose: Optimizing individualized clinical care in heterogeneous rare disorders, such as primary mitochondrial disease (PMD), will require gaining more comprehensive and objective understanding of the patient experience by longitudinally tracking quantifiable patient-specific outcomes and integrating subjective data with clinical data to monitor disease progression and targeted therapeutic effects. Methods: Electronic surveys of patient (and caregiver) reported outcome (PRO) measures were administered in REDCap within clinical domains commonly impaired in patients with PMD in the context of their ongoing routine care, including quality of life, fatigue, and functional performance. Descriptive statistics, group comparisons, and inter-measure correlations were used to evaluate system feasibility, utility of PRO results, and consistency across outcome measure domains. Real-time tracking and visualization of longitudinal individuallevel and cohort-level data were facilitated by a customized data integration and visualization system, MMFPTableau. Results: An efficient PRO electronic capture and analysis system was successfully implemented within a clinically and genetically heterogeneous rare disease clinical population spanning all ages. Preliminary data analyses demonstrated the flexibility of this approach for a range of PROs, as well as the value of selected PRO scales to objectively capture qualitative functional impairment in four key clinical domains. High inter-measure reliability and correlation were observed. Between-group analyses revealed that adults with PMD reported significantly worse quality of life and greater fatigue than did affected children, while PMD patients with nuclear gene disorders reported lower functioning relative to those with an mtDNA gene disorder in several clinical domains. Conclusion: Incorporation of routine electronic data collection, integration, visualization, and analysis of relevant PROs for rare disease patients seen in the clinical setting was demonstrated to be feasible, providing prospective and quantitative data on key clinical domains relevant to the patient experience. Further work is needed to validate specific PROs in diverse PMD patients and cohorts, and to formally evaluate the clinical impact and utility of harnessing integrated data systems to objectively track and integrate quantifiable PROs in the context of rare disease patient clinical care. | wrong study design |
| 2 | A rare disease patient-reported outcome measure: revision and validation of the German version of the Systemic Sclerosis Quality of Life Questionnaire (SScQoL) using the Rasch model | 2021 | ORPHANET JOURNAL OF RARE DISEASES | Kocher, A and Ndosi, M and Denhaerynck, K and Simon, M and Dwyer, AA and Distler, O and Hoeper, K and KÃ¼nzler-Heule, P and Redmond, AC and Villiger, PM and Walker, UA and Nicca, D | Background: Rare disease patient-reported outcome measures (PROMs) require linguistic adaptation to overcome the challenge of geographically dispersed patient populations. Importantly, PROMs such as health-related quality of life (HRQoL) should accurately capture responses to patient-identified concerns. The Systemic Sclerosis Quality of Life Questionnaire (SScQoL) is a 29-item tool validated in six languages. Previous evaluation of the German version revealed problems with dichotomous responses. This study aimed to revise the German SScQoL, extend the response structure, and evaluate content and construct validity, reliability and unidimensionality. Methods: The instrument validation study involved revising the German SScQoL response structure, cognitive debriefing with patients and validation using Rasch analysis. The revised SScQoL was completed by Swiss-German-speaking patients with SSc within the Swiss MANagement Of Systemic Sclerosis (MANOSS) study. Rasch analysis was employed to test the validity, reliability and unidimensionality of the revised instrument. Results: Based on cognitive debriefing with patients (n = 6) dichotomous items were extended to a polytomous 4-point response structure. A total of 78 patients completed the revised SScQoL. Initial analysis of the 29 items suggested the scale lacked fit to the model (chi(2) = 51.224, df = 29, p = 0.007). Grouping items into five domains resulted in an adequate fit to the Rasch model (chi(2) = 5.343, df = 5, p = 0.376) and unidimensionality (proportion of significant independent t tests: 0.045, 95% CI 0.016-0.114). Overall, the scale was well targeted, had high internal consistency (Person Separation Index, PSI = 0.931) and worked consistently in patients with different demographic and clinical characteristics. Conclusions: The revised German SScQoL has a 4-point response structure and is a valid, reliable measure. Rasch analysis is useful for validating continuous response structure of quality of life measures. Further evaluation of measurement equivalence with other German-speaking cultures is required for multinational comparisons and data pooling. | wrong population |
| 3 | QUALITY-OF-LIFE IN CYSTIC-FIBROSIS | 1995 | PHARMACOECONOMICS | TULLIS, DE and GUYATT, GH | Cystic fibrosis is a chronic, multisystem genetic disease with a wide variability in clinical severity, In recent years, advances in therapy have led to improved patient survival into adulthood. New treatments are rapidly being developed and require evaluation to determine their efficacy, The measurement of health-related quality of life in cystic fibrosis provides additional information about the impact of this disease that cannot be obtained by physiological tests such as pulmonary function. An instrument to measure health-related quality of life is especially useful as an outcome measure for clinical trials. To date, only a few general quality-of-life (QOL) measures have been used in people with cystic fibrosis. There has been some demonstration of validity in 2 measures (the Quality of Well-Being Scale and the Functional Status Index) but the responsiveness of these instruments in this population has not been established. A cystic fibrosis-specific QOL instrument would be valuable as an outcome measure because of its potential for increased responsiveness, but no published measures exist as yet. | wrong study design |
| 4 | The Impact of Activated Phosphoinositide 3-Kinase Î´ Syndrome (APDS) on Health-Related Quality of Life (HRQoL): Elicitation of Health State Utility Values Through Time Trade-Off (TTO) and EQ-5D | 2025 | ADVANCES IN THERAPY | Nolthenius, JBT and Griffiths, A and Dodd, O and Wyn, R and Enstone, A and Whalen, J | IntroductionActivated phosphoinositide 3-kinase delta (PI3K delta) syndrome (APDS) is an ultra-rare inborn error of immunity. APDS is a chronic, heterogeneous, and progressive disease, with individuals experiencing different combinations of multisystemic clinical manifestations which advance over time. To date, there are no published utility values for APDS and there are limited data detailing the long-term effects and impact of APDS on health-related quality of life (HRQoL). This study sought to quantify the impact of individual and combined manifestations on the HRQoL of people with APDS.MethodsTo mitigate the challenges of measuring HRQoL in a rare disease, health state vignettes were developed through interviews with people with APDS and the published literature, and validated by experts, to represent individual or combinations of manifestations frequently experienced by people with APDS. Two valuation methods were then employed to elicit utility values for the health states: EQ-5D with clinical experts from France, Spain, and the UK (N = 4), and time trade-off (TTO) with members of the UK general public (N = 100).ResultsThe results of both EQ-5D and TTO exercises highlight the substantial HRQoL impact of APDS, particularly in those experiencing multiple concurrent manifestations or those diagnosed with lymphoma. Mean utility values ranged from 0.897 to - 0.109 in the EQ-5D exercise and 0.870 to 0.330 in the TTO exercise. Overall, the ranking of the health states by utility values was generally consistent between EQ-5D and TTO exercises. However, points of variance were observed for utility values for severe health states, e.g. those including lymphoma (one of the primary causes of death in APDS) ranged from 0.430 to - 0.109 in the TTO and EQ-5D exercises, respectively.ConclusionThis is the first study to indirectly elicit utility values in APDS through valuation by the general population and clinical experts. This expands on the current literature for APDS by providing an overview of how HRQoL declines with disease progression and increasing number of manifestations. | wrong study design |
| 5 | Measuring what matters: Little evidence supporting the content validity of EQ-5D in people with Duchenne muscular dystrophy and their caregivers. |  |  |  | Comments on an article by Norah L. Crossnohere et al.(see record [rid]2021-18083-011[/rid]). The authors welcome the research by Crossnohere et al., but we would like to make explicit the caveat to their conclusion that the researchers conducted a very limited assessment of the content validity of the EQ-5D for use in measuring health status (or health-related quality of life, as used elsewhere in the article) in DMD. While this is acknowledged in the Discussion section of the article, it is not clear in the Methods section or in the abstract, and there is the concern that this caveat may therefore be lost on a more casual reader. Crossnohere et al. conclude their article recommending that â€˜â€˜advocacy groups look holistically at addressing the barriers to access of therapies in rare diseases such as Duchenne, rather than honing in specifically on perceived shortcoming of the EQ-5D.â€™â€™ We would extend this to say that all stakeholders need to consider how value is determined in access decisions and that, for rare diseases, where there is a paucity of clinical evidence and knowledge, modeling of value must capture elements that are most important to patients, including effects on quality of life. (PsycInfo Database Record (c) 2025 APA, all rights reserved) | wrong publication type |
| 6 | Further evidence of reliability and validity of the Huntingtonâ€™s disease quality of life battery for carers: Italian and French translations. |  |  |  | Background: Existing research suggests that family caregivers of persons with Huntingtonâ€™s disease (HD) face a distinct series of problems, linked to the complex nature of the disease. Aubeeluck and Buchanan (Clin Genet, 71(5): 434â€“445, 2007) developed and validated a disease-specific measure used to explore caregivers quality of life and assess the efficacy of therapeutic interventions. This current study builds on this research through the validation of French and Italian translations of the Huntingtonâ€™s disease quality of life battery for carers (HDQoL-C). Method: A total of 301 family carers completed the HDQoL-C. Participants were recruited through the "Euro- HDB" study which is measuring the burden in HD across Europe and the USA. Results: Factor analysis demonstrated good internal consistency, reliability and congruent validity. Carers who cared for patients with less clinically severe symptoms reported significantly better QoL than carers of patients with more clinically severe symptoms. Discussion: Findings indicate the HDQoL-C is multi-lingual, multi-cultural and easily applicable in other languages. (PsycInfo Database Record (c) 2021 APA, all rights reserved) | wrong publication type |
| 7 | The Patient Perspective on the Impact of Tenosynovial Giant Cell Tumors on Daily Living: Crowdsourcing Study on Physical Function and Quality of Life | 2018 | INTERACTIVE JOURNAL OF MEDICAL RESEARCH | Mastboom, MJ and Planje, R and van de Sande, MA | Background: Tenosynovial giant cell tumor (TGCT) is a rare, benign lesion affecting the synovial lining of joints, bursae, and tendon sheaths. It is generally characterized as a locally aggressive and often recurring tumor. A distinction is made between localized-and diffuse-type. The impact of TGCT on daily living is currently ill-described. Objective: The aim of this crowdsourcing study was to evaluate the impact of TGCT on physical function, daily activities, societal participation (work, sports, and hobbies), and overall quality of life from a patient perspective. The secondary aim was to define risk factors for deteriorated outcome in TGCT. Methods: Members of the largest known TGCT Facebook community, PVNS is Pants!!, were invited to an e-survey, partially consisting of validated questionnaires, for 6 months. To confirm disease presence and TGCT-type, patients were requested to share histological or radiological proof of TGCT. Unpaired t tests and chi-square tests were used to compare groups with and without proof and to define risk factors for deteriorated outcome. Results: Three hundred thirty-seven questionnaires, originating from 30 countries, were completed. Median age at diagnosis was 33 (interquartile range [IQR]=25-42) years, majority was female (79.8% [269/337]), diffuse TGCT (70.3% [237/337]), and affected lower extremities (knee 70.9% [239/337] and hip 9.5% [32/337]). In 299 lower-extremity TGCT patients (32.4% [97/299]) with disease confirmation, recurrence rate was 36% and 69.5% in localized and diffuse type, respectively. For both types, pain and swelling decreased after treatment | Wrong outcome |
| 8 | Patient experience in retinitis pigmentosa and Choroideremiaâ€“ a concept elicitation study in 17 patients based on qualitative interviews | 2025 | Orphanet J. Rare Dis. | Rometsch, E. and Thuresson, P.-O. and Hurst, N. and Eule, E. and Bachmeier, I. and Blanchard, B. and Sahel, J.-A. and Audo, I. | Background: Retinitis pigmentosa (RP) and Choroideremia (CHM) are rare inherited retinal diseases (IRDs) that can lead to severe visual impairment or blindness. Despite different underlying genetic pathways, they have many progression patterns in common, first affecting the more peripheral retina and later advancing toward the central retina. Early symptoms of both diseases include night blindness, difficulty adjusting to changing levels of light, and difficulty seeing in poor contrast. This is followed by progressive loss of the peripheral visual field in daylight, and eventually blindness. Currently, hardly any studies based on interviews that describe symptom experience and impact exist. In this concept elicitation study, we conducted qualitative interviews to identify the symptoms experienced by RP (n = 12) and CHM (n = 5) patients, and to understand the impact of symptoms on patientsâ€™ lives. Results: Among the 14 symptoms reported, poor night vision/night blindness, difficulty seeing in bright light, and difficulty seeing in low/dim light were experienced by all participants. Over 50% of participants in either condition reported difficulty adapting from bright to dark and vice versa, poor peripheral vision, poor contrast sensitivity, poor distance vision, and poor visual acuity. Symptoms had a significant impact on activities of daily living. Most commonly impacted were the ability to navigate and the use of digital screens (n = 17/17, 100%) as well as physical functioning and work/school-related activities (n = 16/17, 94.1%). These impacts were often exacerbated by environmental factors. For instance, all participants reported this for navigation. Additionally, most participants (n = 13/17, 76.5%) reported impacts on emotional well-being. Concept saturation was shown for the RP sample (n = 12) and the combined sample (n = 17), i.e., all concepts reported were spontaneously mentioned in the first 3 sets of interviews. This suggests that further interviews would be unlikely to yield any new symptom concepts. Conclusion: The patient interviews provided insight into the patient experience of RP and CHM and formed the basis for developing a combined conceptual model of RP and CHM disease experience, suitable to serve as basis for future patient reported and performance outcome measures. Testing in larger samples, especially in CHM, is recommended to further evaluate content validity of these preliminary findings. | wrong study design |
| 9 | Acquired angioedema due to C1-inhibitor deficiency: Patient experience and assessment of patient-reported outcome measures | 2025 | Allergy Asthma Clin. Immunol. | Zanichelli, A. and Cohn, D.M. and Riedl, M.A. and Clifford, S. and Romero, B. and Brewer, K. and Pattanaik, S. and Chen, M. and Mendivil, J. | Rationale: Acquired angioedema due to C1-inhibitor deficiency (AAEC1- INH) is a rare disease mediated by bradykinin and characterized by painful swelling attacks. There are currently no approved prophylactic or on-demand treatments and no patient-reported outcome (PRO) measures validated for use in AAE-C1-INH. This study aimed to (1) create a conceptual model of AAE-C1-INH to describe patients' experiences of symptoms and impact of AAE-C1-INH and (2) explore and confirm the relevance of adapting PRO measures validated for hereditary angioedema, another bradykinin-mediated disease with similar manifestations, for AAE-C1-INH. Methods: This cross-sectional, remote, qualitative study included adults in the United States with AAE-C1-INH and no prior or concomitant diagnosis of other angioedema. A semi-structured interview guide with open-ended questions elicited patients'descriptions of AAE-C1-INH manifestations and impact on their daily lives. Cognitive interviews assessed perceptions of clarity, comprehension, and meaningful levels of change of specific PRO measures, including Patient Global Impression of Change (PGI-C) and Severity (PGI-S) and two health-related quality-of-life (HRQoL) measures: Patient Global Assessment of Change (PGA-C) and Status (PGA-S). Results: Of eight individuals interviewed (seven female | Wrong outcome |
| 10 | Using cognitive debriefing interviews to optimize measurement of health-related quality of life among adolescents and adults with hereditary angioedema-a rare disease | 2024 | Qual. Life Res. | Broderick, L. and Foster, A. and Waldman, L.T. and Jacobs, K.D. and Bordone, L. and Yarlas, A. | Aims: Hereditary angioedema (HAE) is a rare, genetic disorder characterized by disabling episodes of localized skin swelling, painful abdominal attacks, and life-Threatening laryngeal attacks. While the impact of angioedema attacks on health-related quality of life (HRQoL) has been documented, there is a lack of evidence pointing to a fit-for-purpose instrument for measuring those impacts for patients with HAE. Cognitive debriefing (CD) interviews were conducted to determine whether an existing instrument, the Angioedema QoL Questionnaire (AE-QoL), is content valid and appropriate for capturing the impact of angioedema attacks on HRQoL in adults and adolescents with HAE. Methods: Adults (age 18? years) and adolescents (ages 12'17 years) with HAE participated in 60-min, audiorecorded interviews to assess the relevance, comprehensiveness, and comprehensibility of the AE-QoL. Participants used a think-Aloud approach to complete the AE-QoL, allowing for spontaneous identification of potential problems, followed by verbal probing with specific questions about each element of the instrument. All participants were recruited through a partnership with a patient advocacy group | Wrong outcome |
| 11 | QALYs and rare diseases: exploring the responsiveness of SF-6D, EQ-5D-5L and AQoL-8D following genomic testing for childhood and adult-onset rare genetic conditions in Australia | 2023 | Health Qual. Life Outcomes | Pan, T. and Wu, Y. and Buchanan, J. and Goranitis, I. | Background: Genomic testing transforms the diagnosis and management of rare conditions. However, uncertainty exists on how to best measure genomic outcomes for informing healthcare priorities. Using the HTA-preferred method should be the starting point to improve the evidence-base. This study explores the responsiveness of SF-6D, EQ-5D-5L and AQoL-8D following genomic testing across childhood and adult-onset genetic conditions. Method: Self-reported patient-reported outcomes (PRO) were obtained from: primary caregivers of children with suspected neurodevelopmental disorders (NDs) or genetic kidney diseases (GKDs) (carersâ€™ own PRO), adults with suspected GKDs using SF-12v2 | Wrong outcome |
| 12 | How does the EQ-5D-5L perform in the field of rare diseases? An exploratory study among adult patients with Neurofibromatosis type 1 in China | 2023 | Qual. Life Res. | Zhang, L. and Liang, W. and Suo, Y. and Cao, S. and Yang, L. and Jin, X. | Aims: In rare disease areas, health-related quality-of-life (HRQL) measures with good measurement properties play a vital role in evaluating the efficacy and cost-effectiveness of innovative interventions. Neurofibromatosis type 1 (NF1) is a rare neurogenetic disorder that affects multi-system and significantly impacts patients' HRQL. However, there is a lack of evidence of measurement properties of HRQL measures, especially generic measures, in this disease area. This study aimed to examine the performance of the EQ-5D-5L in measuring HRQL of patients with NF1. Methods: In an online cross-sectional survey, adult patients with NF1 were recruited through a national patient-network across China. Patients' HRQL was assessed by the EQ-5D-5L and the PedsQL-NF1 module (disease-specific). Response patterns of EQ-5D-5L were described using ceiling and floor effects. Convergent validity of the EQ dimensions against the similar constructs of PedsQL-NF1 was examined using Spearman's correlation (rho). Known-group validity was examined by average household income ([CNY30,000 vs. B CNY30,000), family-care demand (yes vs. no), and PedsQL-NF1 overall score ([median vs. B median). Informativity and discriminatory power were assessed using the Shannon (H0) and Shannon evenness (J0) indices. A higher H0(0 to 2.32 for EQ-5D-5L) and J0(0 to 1) indicates more information and better discriminatory power. Results: A total of 226 participants with NF1 completed the EQ-5D-5L and the PedsQL-NF1 module (age 31.5 Â± 7.6, female63.7%). The EQ-5D-5L utility index was 0.84 Â± 0.17, and the average PedsQL-NF1 overall score was 68.3 Â± 15.6. Only 14.2% patients reported no problems on all EQ-5D dimensions, and we did not observe floor effect in our study sample. The hypothesis of known-group analysis was confirmed in the predefined known groups. Regarding the convergent validity, EQ dimensions were moderately correlated with similar constructs of PedsQL-NF1 [rho(mobility with balance) = - 0.51 | Wrong outcome |
| 13 | Measurement properties of the EQ-5D-Y-3L among underage patients with neurofibromatosis type 1 in China | 2023 | Qual. Life Res. | Liang, W. and Cao, S. and Suo, Y. and Zhang, L. and Yang, L. and Wang, P. and Wang, H. and Jin, X. | Aims: Neurofibromatosis Type 1 (NF1) is a rare genetic disorder. PedsQLTM Generic Core Scales (PedsQL GCS) is one of the most popular instruments to measure health-related quality of life (HRQoL) in underage NF1 patients. EQ-5D-Y-3L is a preferencebased generic (child-specific) HRQoL measure that can be used in economic evaluations with a published value set for Chinese population. There is no evidence of EQ-5D-Y-3L's performance among children with NF1 in China. We aimed to examine the performance of the EQ-5D-Y-3L against the PedsQL GCS among children with NF1 in China. Methods: We used data from a nationwide cross-sectional study conducted between Nov 2022 and Jan 2023. The EQ-5D-Y-3L proxy version, PedsQL GCS proxy version, and Zarit Burden Interview (ZBI) were used to estimate the humanistic burden of NF1 patients under 18 years old in China (n = 223). Participants who completed both the EQ-5D-Y-3L and PedsQL GCS were included in this study (n = 154). Measurement properties of the EQ-5D-Y-3L were estimated by (1) ceiling and floor effects and distribution across severity levels of each dimension to demonstrate response patterns | Wrong outcome |
| 14 | THE EXPERIENCE OF PAIN, QUALITY OF LIFE AND SYMPTOMS OF ANXIETY AND DEPRESSION IN ADOLESCENTS AND YOUNG ADULTS WITH AUTOSOMAL DOMINANT POLYCYSTIC KIDNEY DISEASE (ADPKD) | 2023 | Pediatr. Nephrol. | Gimpel, C. and Willem, L. and Van Laere, E. and Vanmeerbeek, L. and Vennekens, R. and Voets, T. and Vanhonsebrouck, K. and Schaefer, F. and Luyckx, K. and Mekahli, D. | Aims/Purpose: ADPKD is often still considered an 'adult' disease, with adults showing severe disease manifestations, pain, reduced quality of life (QoL) and increased psychosocial symptoms. Although ADPKD has mainly asymptomatic manifestations in childhood, the first aim of our study is to investigate QoL, pain, anxiety and depression in adolescents and young adults affected by ADPKD and compare scores to reference populations. Second, we aim to investigate the intra-familial relationship between the disease experience of the young person and disease experience and feelings about genetic disease transmission of their affected parent. Finally, we aim to develop a novel age- and disease-specific instrument (SAIL-ADPKD), that facilitates capturing these patient-centered aspects of ADPKD in future studies with adolescents. Methods: We asked 11- to 25-year-old patients with ADPKD followed at UZ Leuven and age-matched healthy controls to complete surveys of pain (PROMIS pain interference short form) and QoL (PedsQLâ„¢ 4.0 generic core scales) and a behavioral screening questionnaire (Strengths and Difficulties Questionnaire (SDQ)). Parental burden of disease is captured with the ADPKD-IS (impact scale), ADPKD-PDS (pain and discomfort scale), SF36 (Short-Form 36) and a modified Genetic Psychosocial Risk Instrument (GPRI-ADPKD).With regard to the final aim, we created a novel instrument based on symptom frequency recorded in the APedKD registry, core outcomes defined in the multidisciplinary SONG-kids and SONG-PKD initiative, patient testimonies of pain as well as personal experience from pediatric ADPKD clinics. The questionnaire was improved by the feedback of psychologists, a psychometrician, a patient representative, and external experts in pediatric nephrology. Results: Data collection will be finished in spring 2023. We firstly hypothesize that adolescents and young adults with ADPKD have a significant burden of pain due to ADPKD, reduced QOL and increased symptoms of anxiety and depression. Secondly, we hypothesize positive intra-familial associations. Finally, we will test whether the SAILADPKD is a valid and reliable instrument, by analyzing internal consistency, test-retest reliability, and construct validity by convergence with the above-mentioned scores and correlation to measures of biochemical and radiological disease severity. Conclusion: In summary, this study will add evidence on understanding and measuring disease burden in early ADPKD and deepen our understanding of the inter-generational aspects of disease experience in autosomal dominant disorders. | wrong publication type |
| 15 | Psychometric properties of QI-Disability in CDKL5 Deficiency Disorder: Establishing readiness for clinical trials | 2023 | Epilepsy Behav. | Saldaris, J.M. and Jacoby, P. and Leonard, H. and Benke, T.A. and Demarest, S. and Marsh, E.D. and Downs, J. | CDKL5 Deficiency Disorder (CDD) is a rare genetic disorder with symptoms of epilepsy, developmental impairments, and other comorbidities. Currently, there are no outcome measures for CDD with comprehensive evidence of validation. This study aimed to evaluate the psychometric properties of the Quality of Life Inventory-Disability (QI-Disability) in CDD. Quality of Life Inventory-Disability was administered to 152 parent caregivers registered with the International CDKL5 Disorder Database (ICDD). Confirmatory factor analysis was conducted and the goodness of fit of the factor structure was assessed. Fixed-effects linear regression models examined the responsiveness of QI-Disability to reported changes in child health. A subset of parent caregivers (n = 56) completed QI-Disability, as well as additional health-related questions, on two occasions separated by four weeks to evaluate testâ€“retest reliability. Test-retest reliability was assessed using intra-class correlations (ICCs) calculated from QI-Disability scores. Based upon adjustments for changes in child health, ICCs were recalculated to estimate responsiveness to change. Confirmatory factor analysis, internal consistency, and divergent validity were mostly satisfactory, except divergent validity was not satisfactory for the Social Interactions and Independence domains. The Physical Health, Social Interactions, Leisure, and Total scores responded to changes in the child's Physical health, and the Negative Emotions and Leisure domains responded to changes in the child's behavior. Unadjusted and adjusted ICC values were above 0.8 for the Positive Emotions, Negative Emotions, Social Interactions, Leisure, Independence domains and Total score, and above 0.6 for the Physical Health domain. Findings suggest that QI-Disability is suitable to assess the quality of life of children and adults with CDD and could be of value for upcoming clinical trials. | wrong outcome |
| 16 | Quality of life in patients with Niemann-Pick disease type C and their carers | 2023 | Mol. Genet. Metab. | Pountney, J. and Donnelly, C.B. | Background: Patient reported quality of life (QoL) is increasingly recognised is important in assessing care quality and the efficacy of therapy. There are many challenges in the collection of this data in rare disease populations. Niemann-Pick type C (NPC) quality of life instruments (NPCQLQ) for adults and children have been developed for Niemann-Pick disease in Aston University in 2019. However, to ensure the questionnaires reflect patient quality of life, research will be undertaken to further test the questionnaires. Methods: A PhD studentship has been funded by the INPDR, NPUK, NNPDF and INPDA to undertake a study to measure QoL of patients and carers with NPC. The INPDR will collect NPC data from an international population through the patient-reported database. Ethical approval has been granted to undertake analysis of the registry data in a secure data safe haven. Statistical analysis will assess reliability (consistency) and validity (accuracy) of the NPCQLQ. This will include comparisons between NPCQLQ and general quality of life data and reported symptoms and severity, analysis of the internal consistency of the NPCQLQ and validity of the questionnaire in measuring changes in QoL through time. Further analysis will support development of shorter forms of the questionnaire to reduce demands on participants and enable their rapid use in clinical consultations. Additional analysis undertaken will include exploring the impact of NPC on QoL of both patients and carers. Conclusions: A fully validated NPCQLQ will contribute toward providing patients and carers with a tool to report their experience in a robust and meaningful way and contribute evidence to support health technology assessment. A validated NPCQLQ may be included as an endpoint in clinical trials and will help determine if experimental therapies result in meaningful changes to QoL of patients. | wrong publication type |
| 17 | Modification of the Osteogenesis Imperfecta Quality of Life Scale - Paediatric version (OIQoL-P) and Development of a Parent-Report version | 2023 | JBMR Plus | Gibbons, E. and Doll, H. and Hartford, C. and Durnell, K. and Abetz-Webb, L. and Clancy, J. and Hill, C. | OBJECTIVES: Evaluate the content validity of the 33-item OIQoL-P, modify and shorten as necessary, and develop a parent-report instrument (OIQoL-O) for younger children unable to self-report. METHODS: Osteogenesis imperfecta (OI) is a rare genetic disorder of the connective tissue characterized by bone fragility and reduced bone mass. A literature review was conducted to explore the impact of OI on child health-related quality of life (HRQoL). Qualitative interviews were then undertaken in OI healthcare professionals (HCPs) in the United States (US n=2) and United Kingdom (UK n=2) to discuss the impact of the condition and assess the content validity of both the OIQoL-P and new OIQoL-O. Three rounds of interviews were conducted with a total of 15 parent and child dyads recruited from patient advocacy groups in the US and UK to inform sequential modifications of the measures. Each interview was conducted in two parts: Concept elicitation (CE) to understand participants' lived experience of OI and cognitive debriefing (CD) to examine the relevance, appropriateness, and understanding of OIQoL-P and OIQoL-O content (items, instructions, response options, recall period) in children and parents, respectively. RESULTS: The OIQoL-P conceptual model was supported by the literature review. Both concepts present and not present in the model were discussed in the HCP interviews to inform revisions to the OIQoLP. HCPs indicated potential redundancy in some items and suggested additional areas of clarification. The modified measures were found acceptable in the dyad interviews, with the items and instructions beingwell understood, although some items were considered to require a longer recall period, and the relevance of others questioned. Issues were identified with the 'never' response option in the context of a one-week recall period, and response distributions suggested some items of the OIQoL may not be perceived to be relevant by children with OI | Wrong Outcome |
| 18 | Quality of life in Osteogenesis Imperfecta: Evaluation of the content validity of the OIQoL questionnaire in an international cohort | 2023 | JBMR Plus | Hill, C. | OBJECTIVES: Osteogenesis Imperfecta (OI) is a rare hereditary condition characterised by bones that break more easily, hypermobility, short stature and deformity. Quality of life (QoL) is a multifaceted concept described in different ways by different people at differing times in their life. As a result it can be complex to measure and difficult to describe and explain. The OIQoL questionnaire was developed using bottom up methodology and followed Food and Drug Administration guidelines. Development took place within the UK, which has a particular set of environmental, social and economic circumstances that could well impact on the content of the questionnaire. This multi-centre international study aimed to explore quality of life in children with OI, comparing the emergent themes to those previously identified in the UK and examining the content validity of the OIQoL questionnaire. METHODS: Qualitative focus groups of young people, with OI were undertaken in eight countries | Wrong Outcome |
| 19 | Development of a quality-of-life (QoL) questionnaire for hereditary spastic paraplegia (HSP) patients and caregivers | 2022 | Mov. Disord. | Malina, J. and SchrÃ¶der, B. and HÃ¼ÃŸler, E. and KÃ¼hl, T. and Klebe, S. | Objective: Patient reported outcomes like Health-Related Quality of Life are one of the key outcome parameters of clinical studies. The aim of this project within the German â€œTreatHSPâ€ consortium is to develop patient and caregiver reported outcomes (PROs and ObsROs) specific for hereditary spastic paraplegia (HSP). Background: Hereditary spastic paraplegia (HSP) is a group of genetic neurodegenerative diseases. Currently >80 different genetic forms are known. Clinical presentation of HSP is highly heterogeneous as well. Nevertheless, the leading symptom includes a spastic gait disorder caused by degeneration of the pyramidal tract. The worldwide prevalence is 1-9:100 000, making it a rare disease. The â€œSpastic paraplegia rating scaleâ€ (SPRS) is being broadly used as a validated clinical outcome parameter. However, this scale does not provide any information about the healthrelated quality of life (HRQoL) of HSP patients. In our opinion, the HRQoL is an integral component for the assessment of trial readiness, future treatment studies and follow-up visits. Methods: In the current study PROs and ObsROs will be developed by a three-step approach with first qualitative patient interviews to generate specific items, followed by a pilot testing and then validation in a larger patient sample. Semi-structured guided interviews were conducted on 36 patients and 18 caregivers. In order to ensure that patients with different degrees of disease severity are represented, they were classified and selected according to SPRS as â€œmildlyâ€ (SPRS <15 points), â€œmoderatelyâ€ (SPRS 16 -30 points) and â€œseverelyâ€ affected (SPRS>31 points). The interviews were subsequently analyzed using a commercially available software for qualitative and mixed methods research. Results: The pilot HRQoL questionnaires for HSP patients and their caregivers with 34 items each, involving 8 domains of daily life, were developed based on the collected data. The first samples of 65 patients and 57 caregivers are currently undergoing statistical analyses (first-step validation). Depending on the results, items will be adjusted, if necessary. This will be followed by validation on a larger sample of at least 300 patients and their caregivers. Conclusions: A specific HRQoL questionnaire is an unmet need for HSP. It will be an indispensable component for the future qualitative high standard clinical trials and the natural course of HSP. | wrong publication type |
| 20 | Psychometric validation of patient-reported outcome (PRO) and observer-reported outcome (ObsRO) measures in Retinitis Pigmentosa and Leber Congenital Amaurosis | 2022 | Invest. Ophthalmol. Vis. Sci. | Audo, I.S. and Williamson, N. and Barclay, M. and Sims, J. and Boparai, K. and Patalano, F. and Naujoks, C. and Spera, C. and Banhazi, J. and O'Brien, P. and Kay, C. and Green, J. and Durham, T. and Bradley, H. and Fischer, M.D. | Purpose : Retinitis Pigmentosa (RP) and Leber Congenital Amaurosis (LCA) are rare inherited retinal degenerative disorders. There is no RP/LCA-specific measure of visual impairments and impacts on vision-dependent activities of daily living (ADL). The Visual Symptom and Impact Outcomes patient-reported outcome (ViSIO-PRO) and observerreported outcome (ViSIO-ObsRO) measures were developed through qualitative research with RP/LCA patients, caregivers of child patients and expert clinicians. This study evaluated the psychometric properties of the instruments. Methods : The 49-item ViSIO-PRO and 27-item ViSIO-ObsRO instruments were completed by 83 adult and adolescent patients and 22 caregivers of child patients aged 3-11 years with RP/LCA, respectively, at baseline and 12-16-day follow-up. Concurrent measures were also administered at BL. Psychometric analyses assessed item (question) properties, dimensionality, scoring, reliability, validity, and score interpretation. Results : Responses to the ViSIO-PRO and ViSIO-ObsRO items were mainly evenly distributed across response options. Inter-item correlations were mostly moderate-tostrong (>0.30) at baseline within hypothesized domains. Item properties, qualitative data and clinical input supported retention of 35 ViSIO-PRO items and 25 ViSIO-ObsRO items. Confirmatory factor analysis supported a four-factor model (visual function symptoms, mobility, vision-dependent ADL and broader health-related quality of life). A bi-factor model supported calculation of total scores and domain scores. Internal consistency was high for all domain and total scores (Cronbach's alpha>0.70). Test-retest reliability for the total scores was strong between BL and 12-16-day follow-up (Intraclass Correlation Coefficients=0.66-0.98). Convergent validity was supported by strong correlations in a logical pattern with concurrent measures at baseline. There were statistically significant differences in mean ViSIO-PRO and ViSIO-ObsRO scores between known-groups defined by global impression items at baseline. Distribution-based methods provide initial insights to guide interpretation of scores. Conclusions : Findings supported item reduction, established the scoring of the ViSIOPRO and ViSIO-ObsRO instruments, and provided evidence of reliability and validity as outcome measures in RP/LCA. | wrong publication type |
| 21 | Mapping the Haem-A-QoL to the EQ-5D-5L in patients with hemophilia | 2022 | Qual. Life Res. | Xu, R.H. and Dong, D. and Luo, N. and Wong, E.L.-Y. and Yang, R. and Liu, J. and Yuan, H. and Zhang, S. | Objective: This studyâ€™s objective was to develop an algorithm that mapping the Haem-A-QoL scores to EQ-5D-5L utility scores in patients with hemophilia in China. Methods: A national sample of 862 patients with hemophilia completed both the EQ-5D-5L and Haem-A-QoL instruments. Eight regression models were selected to develop the mapping algorithm, they were: the ordinary least squares, general linear regression, Tobit regression, censored least absolute deviation, mixture beta regression, adjusted limited dependent variable mixture, the two-part, and robust MM-estimator model. Root mean squared error (RMSE), mean absolute error (MAE), and R-square (R2) calculated using the tenfold cross-validation and random sample validation methods were used to assess the predictive ability of the models. Results: Based on RMSE, MAE, and R2, the mixture beta regression model with selected Haem-A-QoL subscale scores as the predicted variables showed the best performance. Conclusions: Our mapping algorithm bolsters the calculation of QALYs while conducting an economic evaluation of hemophilia-related interventions when only Haem-A-QoL data are available. The external validity of the algorithm should be further assessed in the other populations. | wrong study design |
| 22 | Validity and Reliability of the Fibrodysplasia Ossificans Progressiva Physical Function Questionnaire (FOP-PFQ), a Patient-Reported, Disease-Specific Measure | 2020 | J. Bone Miner. Res. | Pignolo, R.J. and Kimel, M. and Whalen, J. and Kawata, A. and Revicki, D. and Marino, R. and Kaplan, F.S. | Background: Fibrodysplasia ossificans progressiva (FOP) is an ultra-rare genetic disorder characterized by heterotopic ossification (HO), leading to progressive disability and functional limitations in joint mobility. To measure the ability of individuals with FOP to conduct activities of daily living, age-specific versions of the FOP physical function questionnaire (FOP-PFQ) were designed based on FDA guidance on patient-reported outcome (PRO) development.Objective: To evaluate the reliability and validity of the FOP-PFQ. Methods: Test-retest reliability was evaluated by comparing scores between Baseline and Week 1-3 visits in an FOP natural history study (NHS | Wrong outcome |
| 23 | PRO51 RESPONSIVENESS AND ACCEPTABILITY OF THE EQ-5D TO AN INTERNATIONAL RARE DISEASE COMMUNITY | 2019 | Value Health | Crossnohere, N. and Fischer, R. and Peay, H. and Bridges, J.F.P. | Objectives: Regulatory agencies may share data internationally to inform decision-making regarding orphan drug treatments, giving local research potentially global impact. This study explores the responsiveness and acceptability of the widely-used EQ-5D in measuring health-status across an international sample of Duchenne muscular dystrophy patients and caregivers. Methods: A community-engaged cohort study is documenting the experience of adults with Duchenne, caregivers of affected adults, and caregivers of minors from Australia, Belgium, Canada, Holland, United Kingdom, & United States (expected N=360). Participants completed the EQ-5D (3L, 3LProxy1, YProxy1). ANOVA tests explored QALY and VAS scores across respondent groups and country, and regression explored scores adjusted for patient-age and significant independent predictors. Z-tests of Likert-type items evaluated the EQ-5D for ease of understanding and answering, relevance, and reflection of â€œreal healthâ€ in comparison with a 75% agreement-rate. Results: A preliminary sample of 22 adults, 24 caregivers of adults, 69 caregivers of minors from Australia, UK, and US participated. QALY and VAS scores were predicted by participant role (P<0.001), but not by country (P>0.05). The mean QALY was 0.37 (range: 0.16, 0.79) for adults, 0.18 (-0.11, 0.67) for caregivers of adults, and 0.47 (-0.11, 1.0) for caregivers of minors. Adults reported QALY scores 20% higher than caregivers of adults (P=0.01) after age-adjustment. The mean VAS score was 79 (range: 30, 99) among adults, 55 (15, 90) among caregivers of adults, and 68 (30, 100) among caregivers of minors. Adults reported average VAS scores 24 points above caregiver groups after age adjustment (P<0.01). All groups met or exceeded the 75% instrument acceptability agreement-rate (P<0.05). Conclusions: EQ-5D was responsive and acceptable for this multi-national rare disease sample. While adults consistently rated their health higher than caregivers in preliminary data, the difference between groups was attenuated using the population-derived QALY estimates as compared to self-evaluation using the VAS. | wrong publication type |
| 24 | Translation of the quality-of-life measure for adults with primary ciliary dyskinesia and its application in patients in Brazil | 2019 | J Bras Pneumol | Queiroz, A.P.L. and Athanazio, R.A. and Olm, M.A.K. and Rubbo, B. and Casal, Y.R. and Lucas, J. and Behan, L. | Primary ciliary dyskinesia (PCD) is a genetic disorder that is typically inherited in an autosomal recessive manner. It is clinically characterized by recurrent respiratory infections. However, its repercussions for patient quality of life should not be overlooked. Studies have shown that PCD has a significant impact on the lives of patients, although there are as yet no PCD-specific markers of quality of life. To address that problem, researchers in the United Kingdom developed a quality-of-life questionnaire for patients with PCD. The present communication focuses on the process of translating that questionnaire into Brazilian Portuguese, through a partnership between researchers in Brazil and those in the United Kingdom, as well as its subsequent application in patients in Brazil. | wrong publication type |
| 25 | Assessing disease experience across the life span for individuals with osteogenesis imperfecta: Challenges and opportunities for patient-reported outcomes (PROs) measurement: A pilot study | 2019 | Orphanet J. Rare Dis. | Tosi, L.L. and Floor, M.K. and Dollar, C.M. and Gillies, A.P. and Lee, B. and Nagamani, S.C.S. and Rauch, F. and Glorieux, F. and Retrouvey, J.-M. and Esposito, P. and Rush, E. and Bober, M. and Eyre, D. and Gomez, D. and Harris, G. and Jain, M. and Krakow, D. and Orwoll, E. and Raggio, C. and Smith, P. and Hart, T.S. and Cuthbertson, D.D. and Sutton, V.R. and Krischer, J.P. | Background: Patient reported outcome (PRO) information is crucial for establishing better patient-provider communication, improving shared decision-making between clinicians and patients, assessing patient responses to therapeutic interventions, and increasing satisfaction with care. We used the Brittle Bones Disease Consortium (BBDC) Contact Registry for People with OI, managed by the Rare Disease Clinical Research Network (RDCRN) to (1) to evaluate the construct validity of the Patient-Reported Outcome Measurement Information SystemÂ® (PROMISÂ®) to record important components of the disease experience among individuals with OI | Wrong outcome |
| 26 | Development of a Quality of Life Measurement Scale in Hereditary Haemorrhagic Telangiectasia (HHT) Disease. | 2018 | clinicaltrials.gov |  | Brief Summary Hereditary Haemorrhagic Telangiectasia (HHT) is a rare inherited genetic disease of autosomal dominant inheritance with a prevalence of 1/6000. It is manifested by haemorrhages, mucocutaneous telangiectasias and visceral arteriovenous malformations. These symptoms significantly affect the daily lives of patients, their social relationships and their working lives. HAS (Haute AutoritÃ© de SantÃ©) national recommendations focus on assessing and improving the quality of life (QOL) of patients. Many scales for measuring QOL exist but they are most often general and therefore have the disadvantage of not taking into account the particularities of pathologies and their symptoms and do not allow to have a precise vision of their impact on QOL. It is important to be able to evaluate this impact, to determine its nature and to quantify it so that health professional can adapt their proposal for the management of HHT patients. And only the development of a specific and validated QOL measurement scale will allow them to access this information. The aim of this study is to develop a scale of measurement of quality of life in HHT disease and to validate it, a scale specific to HHT, simple and fast to fill by the patients themselves. | wrong publication type |
| 27 | Psychometric Properties of a Modified KINDL-R Questionnaire for Adolescents and Adults, and Construction of a Brief Version, the KINDL-A(dult)B(rief) Questionnaire, KINDL-AB | 2018 | Acta Haematol. | Neuner, B. and Krampe, H. and McCarthy, W.J. and Reinke, S. and Kowalski, D. and Clausnizer, H. and Shneyder, M. and Rocke, A. and Nowak-GÃ¶ttl, U. | Background/Aims: The generic quality of life KINDL-R-questionnaire is validated for use in children/adolescents â‰¤16 years. The aim of this cross-sectional investigation was to modify the KINDL-R questionnaire for use in adults and to validate its psychometric properties. Methods: Five items of the KINDL-R questionnaire were adapted and the newly developed KINDL-A(dult) questionnaire administered to 255 patients with hereditary and acquired bleeding disorders (mean age 53 years). Its internal consistency and convergent and divergent construct validity were investigated and confirmatory factor analysis was used to evaluate the latent factor structure. Results: The KINDL-A questionnaire showed satisfactory reliability, varying construct validity, but inconclusive factor structure. The KINDL-AB(rief) was developed by removing half of the items and combining 2 sub-axes. This led to factor loadings between 0.62 and 0.91 and increased overall fit (Goodness of fit > 0.8 and Root Mean Square Error of Approximation, RMSEA, < 0.08). Results were validated in 966 healthy blood donors (mean age 38 years). In this group, the KINDL-AB questionnaire showed factor loadings between 0.43 and 0.77, Goodness of fit > 0.95 and RMSEA < 0.05. Conclusions: The new KINDL-AB suggests sufficient to good psychometric properties in adult patients with hereditary and acquired bleeding disorders. | wrong population |
| 28 | Qol-gNMD and patient reported functioning in genetic neuromuscular diseases: A new rasch tool | 2018 | Orphanet J. Rare Dis. | Boyer, F.C. and Rapin, A. and Dany, A. and Hardouin, J.B. | Background The 'Quality of Life in genetic Neuromuscular Disease' questionnaire (QoL-gNMD) is a new patient reported outcome (PRO) measure tool specifically designed for patients with a slowly progressive neuromuscular disease with genetic predominant muscular damage. The QoL-gNMD has been developed in 3 domains: 'Impact of Physical Symptoms', 'Self-perception' and 'Activities and Social Participation'. The objective was to construct and develop a PRO tool, easy to use in clinical settings, and validated by rasch model theories. Methods Qol-gNMD was developed using focus groups consisting of expert patients meeting (5 focus group, n = 41, item bank) [1] and then a quantitative construction phase (n = 150, principal component and RMT analysis) [2]. The French version of the QOL-gNMD (26 items) was administered to patients recruited in 9 tertiary hospitals dedicated to genetic neuromuscular diseases (Fig. 1). Each QoL-gNMD domain is measured on a T score metric i.e. a normal distribution with a mean of 50 and a standard deviation of 10. High values represent good quality of life. Standard errors of measurement were estimated and adjusted using rasch model theories (RMT). For each QoL-gNMD domain we estimated, face validity, test retest reproducibility, concordant validity and the conditional minimum detectable changes [3]. Results 315 patients were recruited for psychometric assessment. Each domain showed good psychometric properties (person separation index > 0.7, test-retest ICC>0.7) and fitted the partial credit model (RMT) (Table 1). Concurrent validity was assessed using the WHOQOL-BREF. Estimated conditional MDC were calculated for each possible measure change. Conclusion QoL-gNND is an operational rasch validated measure. QoL-gNMD English version is available. We need psychometric validations of the English version and studies of psychometric properties for each pathology. | wrong publication type |
| 29 | Adaptation of the Questionnaire "Regarding Patient's Quality of Life With Mastocytosis" in the French Language | 2018 | clinicaltrials.gov |  | Brief Summary Mastocytosis is a heterogeneous rare disease. A 27-item questionnaire to assess the quality of life of patients with cutaneous/ indolent systemic mastocytosis was developed in the German language and validated in the English language in 2015. The team of the University CharitÃ© Berlin and the company Moxie - intellectual proprietor of the questionnaire - proposed the use of the recommendations of Baiardini et al. 2010, for linguistic adaptation of this questionnaire in other languages. Detailed Description The first two steps of the program of linguistic translation have already been carried out under the direction and supervision of teams from CharitÃ© University, Berlin and Moxie Company. The result of these two steps is a secondary version of the Mc-QoL questionnaire in French. The last step of this adaptation process in the target language (French, France) needs to be carried out and it is the subject of this study. The objective of this study is the French language adaptation of the Mc-QoL questionnaire in adult patients with indolent cutaneous and / or indolent systemic mastocytosis. During this study, the second version of the Mc-QoL questionnaire in French will be evaluated on 10 adult patients, with cutaneous and / or indolent systemic mastocytosis and native in the French language. The comprehension tests will be carried out by means of the structured interviews during which the investigator will ask if the patient has had difficulties to understand the recommendations, the questionnaires, will verify the interpretation of all the items and the understanding of each word by the patient. In case of problems, the investigator will propose and / or test translation alternatives, or will ask the patient to propose alternatives. Revisions to the second version of the Mc-QoL questionnaire in French will be done to rectify the problems identified in the conceptual equivalence. The result will be a third French version of the Mc-QoL questionnaire, which will be the final version. A report on the interviews will be written in English: it will describe the number of respondents, their age, the time required to complete the questionnaire, the difficulties encountered, the solutions suggested and retained and how the third version of the questionnaire was produced. | wrong publication type |
| 30 | Observational study to develop a treatment-related patient-reported outcome measure in gaucher disease (QOL-ONE PRO1G) | 2018 | HemaSphere | Di Rocco, M. and Cappellini, M.D. and Carubbi, F. and Alpa, M. and Andria, G. and Barbato, A. and Burlina, A. and Giona, F. and Giuffrida, G. and Roccatello, D. and Borin, L.M. and Spada, M. and Papadia, F. and Linari, S. and Salek, S. and Ionova, T. and Oliva, E.N. | Background: Gaucher disease (GD) is a rare autosomal recessive lysosomal disorder that results in the accumulation of sphingolipids in the body's tissues due to glucocerebrosidase enzyme deficiency. GD manifests with vast clinical heterogeneity. Type 1 is the most common form (non neurophatic) with a wide spectrum of signs and symptoms at presentation: some are very mild, others may present variably enlarged liver and spleen which may cause abdominal discomfort. Trombocytopenia and anemia are very common at diagnosis and may be associated with signs and symptoms (bruising and bleeding, tiredness, vertigo, dyspnea and reduced physical functioning). Over 20% of patients experience bone pain or fractures. Treatment is indicated for patients with type 1 GD who exhibit clinical signs and symptoms of the disease, including anemia, thrombocytopenia, skeletal disease, or visceromegaly. Assessment of the impact of illness on physical, mental, and social functioning is an essential element of clinical diagnosis, a major determinant of therapeutic choices and efficacy, and a guide to longer-term care. Furthermore, it is known that patient reported outcomes (PROs) may influence various changes in intervention. There are many generic instruments available to measure the impact of disease on patient's health-related quality of life (HRQoL). However, there is no PRO measure that has been developed specifically for the use in GD. Such a measure would help clinicians to gain a more in-depth understanding of the impact of GD on patients and inform clinical decision making, leading to better patient care and compliance. Aims: The aim of this study is to develop and validate an instrument designed to measure the impact of GD and treatment on individual patients' PROs. Primary endpoints are the generation of items to construct a PRO instrument for patients with GD and its psychometric evaluation. Methods: This is an observational multicentre study. Participants will be GD patients aged â‰¥18 years attending referral centers. The study will be divided in 6 stages: Stage 1 - conceptualisation of PROs in GD patients before the data collection, in order to lay a conceptual foundation for the new instrument | Wrong outcome |
| 31 | Disease burden as assessed by the norfolk quality of life scale in hereditary transthyretin amyloidosis relative to diabetic neuropathy | 2018 | J. Manag. Care Spec. Pharm. | Yarlas, A. and Lovley, A. and Guthrie, S. and Pollock, M. and Sikora Kessler, A. and White, M. | BACKGROUND: Hereditary transthyretin amyloidosis (hATTR) is a rare disease resulting in progressive polyneuropathy and cardiomyopathy. Many symptoms and outcomes associated with hATTR resemble those of a more common health condition, Diabetic Neuropathy (DN). Due to this similarity, an instrument designed to capture multiple dimensions of quality of life (QOL) in patients with DN the Norfolk QOL-DN has been validated with and included as an endpoint in several treatment studies of hATTR patients. Since this instrument has been used to assess QOL in both hATTR and DN patients, it is possible to examine the relative QOL burden of hATTR by comparing it with scores observed for patients with DN. OBJECTIVE: To estimate the QOL burden of hATTR relative to the burden of DN. METHODS: The Norfolk QOL-DN is a 35-item self-reported measure that captures QOL in patients who suffer from neuropathy with scores ranging from-4 to 136 (higher scores represent lower QOL). In addition to an overall QOL score, five domains corresponding to disabilities linked to different nerve fibers can be assessed: physical functioning/large-fiber neuropathy (PF/LPN | Wrong outcome |
| 32 | Development of a new tool for assessing health-related Quality of Life (QoL) in patients with hereditary angioedema (HAE): The United States HAE association (HAEA)-QoL | 2018 | J. Allergy Clin. Immunol. | Christiansen, S.C. and Busse, P.J. and Birmingham, J. and Banerji, A. and Lumry, W.R. and Zuraw, B.L. | RATIONALE: Hereditary Angioedema (HAE) with C1-INH deficiency (HAE-C1-INH) presents with unpredictable episodes of angioedema, which can be potentially life-threatening, and is often disfiguring and interferes with patients' daily activities. A tool to capture the health related quality of life (HRQoL) specific to HAE enables providers and patients to objectively measure the control of disease and the impact of new therapies for this rare disorder. To date, there has not been a questionnaire to measure HRQoL in patients with HAE based upon the management guidelines developed by the United States (US) Medical Advisory Board (MAB) of the HAE-Association (HAEA) and validated with US patients. METHODS: A preliminary 41-item questionnaire was generated by physician members of the HAEA MAB, review of the literature and patient feedback. Content validity and item reduction were performed by administering the questionnaire to patients with HAE-C1-INH attending a US national HAE patient summit. Questions were removed by assessing response rates and exploratory factor analysis (EFA). RESULTS: One hundred and sixty-eight patients with HAE-C1-INH completed the preliminary questionnaire (HAEA-QoL). Response rate and EFA assessment, stratified questions into 2 domains, â€œfeelingsâ€ and â€œconcerns,â€ and identified 12 questions for removal to create the next version of the HAEA-QoL. CONCLUSIONS: A preliminary version of a HRQoL questionnaire for patients with HAE-C1-INH has been developed based upon US management guidelines for HAE and patient feedback. Validation studies needed for FDA approval of a patient reported outcome (PRO) to address reliability, criterion validity, and the ability to detect clinical change over time, are currently in progress. | wrong publication type |
| 33 | Assessment of quality of life in patients with aplastic anemia and/or PNH | 2016 | Oncol. Res. Treat. | Panse, J. | Acquired Aplastic Anemia (AA) and Paroxysmal Nocturnal Hemoglobinuria (PNH) are so-called ultra-rare diseases. While much is known about patho-physiology and treatment of these interrelated diseases, less is known about pts. psycho-social issues. Quality of life (QoL) evaluation tools used in studies for AA and PNH are rather unspecific and were designed for cancer patients (pts) (e.g. the EORTC QLQ-C30). Given the complexity of AA and PNH, the variation in symptoms and treatment approaches, the young age of the pts, and the fact that marrow failure syndromes are not classified as malignant diseases, it is likely that cancer-spe cific questionnaires are inappropriate to adequately assess the QoL and illness intrusiveness in these pts. Hence, we initiated the development and validation of an AA/PNH-specific QoL-instrument (QLQ-AA/PNH). The generation of a QLQ-AA/PNH was performed according to EORTC QoL group guidelines: after identification of QoL issues by literature review, a focus group of pts and physicians were interviewed (phase I). After screening of documented interviews, QoL issues were generated and reworded in a preliminary questionnaire (phase II). In phase III the questionnaire with generated items was tested in a representative patient group and psychometrically validated. In addition, patients were asked to complete a questionnaire regarding their supportive care needs, (e. g. information, support by medical staff, psychosocial counseling, patient support groups etc.) and potential iatrogenic problems (e. g. delay in diagnosis, appreciation of QoL problems by health care professionals). 102 patients in more than 25 German and Swiss cities were interviewed. In phase I, interviews of 19 pts and 8 physicians specialized in AA/PNH-treatment resulted in 649 QoL issues | Wrong outcome |
| 34 | Initial development of patient-reported outcome measures for children, adolescents and adults with primary ciliary dyskinesia: The QOL-PCD | 2015 | Qual. Life Res. | Quittner, A.L. and Leigh, M. and Ferkol, T. and Dell, S. and Morris, A.M. and Behan, L. and Lucas, J. | AIMS: Primary Ciliary Dyskinesia (PCD) is a rare genetic disease (âˆ—1:15,000) characterized by pulmonary infections, bronchiectasis, rhinosinusitis, infertility, and hearing impairment, caused by impaired mucociliary clearance. Our aim was to create age-appropriate, quality of life questionnaires (QOL-PCD) for adults, children and adolescents with PCD and parents of younger children. Uniquely, this process was conducted cross-culturally and harmonized using a protocol-driven approach between the UK and North America. METHODS: Following a literature review and expert panel meeting, open-ended interviews evaluated the impact of PCD on HRQoL in the UK and North America. They were conducted separately with adults (n = 21), children (n = 20), adolescents (n = 20), and parents of younger children (n = 29). Transcripts were content-analyzed using Atlas.ti/Nvivo to derive saturation matrices for each group. Concordance of symptoms between parents and children were examined. Adult patients (n = 49) rated items for relevance. The questionnaire was refined following cognitive interviews with adults (n = 15), children (n = 14), adolescents (n = 16) and parents (n = 17). RESULTS: Saturation matrices confirmed comprehensive coverage of content. The QOL-PCD for each patient/parent group covered 7 domains: Physical Functioning, Emotional Functioning, Treatment Burden, Respiratory&Sinus Symptoms, Ears & Hearing, Social Functioning, Vitality and Health Perceptions. Body Image, Eating, and Digestive Issues were not highly endorsed by participants | Wrong outcome |
| 35 | Extending PROMIS and neuro-QOL to Huntington disease: New measures of chorea, speech and swallowing difficulties, and end of life concerns | 2015 | Qual. Life Res. | Carlozzi, N. and Lai, J.-S. and Schilling, S. and Hahn, E.A. and Kratz, A. and Downing, N. and Paulsen, J. and Nance, M. and Dayalu, P. and Perlmutter, J. and McCormack, M. and Quaid, K. and Ross, C. and Perlman, S. and Miner, J. and Barton, S. and Marin, H. and Geschwind, M. and Rao, S. and Gershon, R.C. and Cella, D. | AIMS: Existing patient-reported outcomes measures (PROs) used in Huntington's disease (HD), a debilitating genetic disease that impacts physical, behavioral, emotional, cognitive, and social health, are typically not comprehensive and do not fully account for clinically meaningful changes in function. While new PROs examining healthrelated quality of life (HRQOL) such as Neuro-QOL (www. neuroqol.org) and PROMIS (www.nihpromis.org) offer solutions to these shortcomings, they do not include HD-specific content. The HDQLIFE study addresses this shortcoming by extending PROMIS/ Neuro-QOL to include HD-specific content. Specifically, the purpose of this study was to develop and calibrate new HD-specific item banks and computer-adaptive tests (CATs) according to established PROMIS methodology. METHODS: Data were collected from 539 individuals with prodromal, early-stage or late-stage HD. Participants completed newly developed HD-specific item pools (159 items) examining chorea, speech and swallowing difficulties, and end of life concerns. Item bank and CAT development included: establishing unidimensionality (exploratory and confirmatory factor analysis [EFA and CFA]), establishing psychometric properties with graded response model (GRM), and excluding items exhibiting differential item functioning (DIF). RESULTS: Chorea. EFA identified 36 unidimensional items | Wrong outcome |
| 36 | Development and Validation of Patient Reported Outcome (PRO) Measures for Individuals With Neurofibromatosis 1 (NF1) and Plexiform Neurofibromas (pNFs) | 2015 | clinicaltrials.gov |  | Brief Summary Background: People with neurofibromatosis 1 (NF1) who have plexiform neurofibromas (pNFs) can have pain that affects their daily lives. This study aims to improve questionnaires that measure their pain, daily living, and physical functioning. Objectives: To examine and improve questionnaires about daily living for people with NF1 and pNFs. Eligibility: People ages 5 and older with NF1 and a pNF Design: Participants will be screened with medical history. This study will have 2 phases. Phase 1 participants will talk about existing pain assessment questionnaires and how pNFs affect their life. They will have group discussions of up to 8 people of a similar age with NF1 and pNFs, or the parents of children with it. These will last about 90 minutes. Children ages 5 to 7 and their parents will have one-on-one meetings instead. These will last about 45 minutes. Discussions will be audiotaped. After the questionnaires have been changed, individual interviews will discuss the new wording, instructions, questions, and electronic format of the new forms. Phase 2 is now complete. Phase 1 participants may be invited to Phase 2. Phase 2 participants will complete the new questionnaires. These may be pen-and-paper or electronic. The questionnaires will take about 30 minutes for adults and teens. Children will work one-on-one with a staff member and may need up to 45 minutes. A small group of participants will be complete the forms twice-in clinic and 1 month later at home. Also, a small group who start a new pain treatment or have a dose increase in their treatment will complete the forms twice-before the treatment change and 1 month later. Detailed Description Background: * Neurofibromatosis 1 (NF1) is a genetic disease with multiple clinical manifestations, including plexiform neurofibromas (pNFs) that can cause pain and may significantly impact daily functioning and quality of life (QOL). * Patient-reported outcomes (PROs) are useful in trials for conditions that are disabling and chronic like NF1, where symptom reduction and improved functioning and QOL currently are important treatment outcomes, which may occur with pNF shrinkage. * A critical step toward approval of drugs to treat pNFs is to evaluate clinical benefit in conjunction with a reduction in tumor volume as assessed by imaging endpoints. * The FDA requests the use of PROs in NF1 clinical trials, especially for assessing changes in symptoms, such as pain. * Currently, no valid PRO measures exist that are specific to the NF1 population to assess pNF pain or its functional impact on an individual s life. Objectives: * Phase 1: Qualitative Evaluation - COMPLETE --To evaluate current modifications and the need for any additional modifications to existing measures of pain intensity (Numeric Rating Scale | Wrong outcome |
| 37 | Psychometric validation of the newly developed phenylketonuria-quality of life (PKU-QOL) questionnaires assessing the impact of phenylketonuria and its treatment on patients' quality of life | 2014 | Value Health | Bosch, A.M. and Burlina, A. and Cunningham, A. and Bettiol, E.O. and Moreau-Stucker, F. and Benmedjahed, K. and Regnault, A. | Objectives: Phenylketonuria (PKU) is a rare genetic disorder impacting phenylalanine (Phe) metabolism. Treatment involves a lifelong Phe restricted diet that is strict and socially demanding. Even when treated early and well, mild cognitive abnormalities have been seen. PKU can affect quality of life in individuals and their families. The phenylketonuria-quality of life (PKU-QOL) questionnaires are the first PKU-specific QOL questionnaires ever developed. The study aimed to perform the psychometric validation of these questionnaires. Methods: An observational study was conducted in France, Germany, Italy, The Netherlands, Spain, Turkey and the UK to finalize and validate PKU-QoL questionnaires in individuals with treated PKU aged 9-11, 12-17 and â‰¥ 18 years, and in parents of individuals < 18 years. Questionnaires were assessed for reliability (internal consistency, test-retest), concurrent validity (using three generic questionnaires adapted to the respondent: PedsQOL, SF-36 for adults and CHQ-PF28) and clinical validity (using PKU severity and overall assessment of patient health status). Results: In total, 559 participants (306 individuals, ages 9-45 years | Wrong outcome |
| 38 | Assessment of the huntington quality of life instrument (H-QOL-I) cross-cultural validity | 2014 | Value Health | Clay, E. and Belhadj, A. and Squitieri, F. and Dorey, J. and Auquier, P. and Zielonka, D. and Trigo, P. and Toumi, M. | Objectives: The Huntington Quality of Life Instrument (H-QoL-I) is the first selfreported specific instrument developed to assess the health-related QoL (HRQoL) of patients with Huntington's disease (HD). It includes three subscales: motor (4 Likert-type items), psychology (4 Likert-type items) and socializing (3 Likerttype items). The aim of the study was to assess whether patients from different countries respond differently to the H-QoL-I instrument. Methods: Data were from the European study of HD burden (EURO-HDB) survey and included data across 6 countries: France, Germany, Italy, Spain, Poland and the USA. The Differential Item Functioning (DIF) method was adopted to examine whether patients from different countries with the same characteristics had different probability of giving a certain response on H-QoL-I. An item was considered as displaying a DIF if the p-value associated with the two-degree-of-freedom Chi-squared test comparing the two ordinal logistic regressions (with/without country effect and an interaction term between the total rest score and country) was lower than 0.01 and the Zumbo-Thomas effect size was higher than 0.130. Zumbo-Thomas effect size measure and associated p-value were calculated for each item and for all pairs of countries (i.e. 15 combinations). Results: The study included 633 patients (176 French, 124 Italian, 44 German, 60 Polish, 59 Spanish and 170 American). No DIF was detected across all combinations of countries for all items. Sixteen pairwise Zumbo-Thomas effect size measures referring to 7 items were found significant but were lower than 0.130. Conclusions: This study did not detect any variation across the studied countries in the assessment of HRQoL of HD patients using the H-QoL-I instrument. These results support the crosscultural validity of the H-QoL-I. | wrong publication type |
| 39 | IHAE-QoL: Specific health-related quality of life (HRQoL) questionnaire in hereditary angioedema due to C1 inhibitor deficiency (HAE-C1INH) | 2014 | J. Allergy Clin. Immunol. | Prior, N. and Remor, E. and PÃ©rez-FernÃ¡ndez, E. and GÃ³mez-Traseira, C. and Caminoa, M.J. and GayÃ¡, F. and Aberer, W. and Barrera, O.M. and Betschel, S.D. and Bouillet, L. and Bygum, A. and Farkas, H. and Grumach, A.S. and Grivcheva-Panovska, V. and Levi, M. and Longhurst, H. and Malbran, A. and Moldovan, D. and Porebski, G. and Reshef, A. and Staubach, P. and Zanichelli, A. and Zhi, Y.-X. and Caballero, T. | RATIONALE: No specific HRQoL questionnaire for HAE-C1INH is available. METHODS: Qualitative analysis of interviews to Spanish HAE-C1INH patients and experts followed by a rating phase to assess content validity led to a draft version of the questionnaire (HAE-QoL). Cross-cultural adaptation and an international rating phase with experts were performed. Resulting version (IHAE-QoL v.1.1), a clinical questionnaire and SF-36v2 were internationally pilot tested (with retest in half of the sample one month later). Data were analyzed with SPSS v.9.0. Criteria for item omission were no response >10%, corrected homogeneity index (CHI) <0.3 or factor loadings <0.4 in the exploratory factor analysis (EFA). Those items with higher loadings per factor were selected. Psychometric properties were assessed. RESULTS: 61 patients and 16 experts participated in the Spanish phase and 14 experts from 13 countries in the international rating phase. A version of 44 items and 9 dimensions was obtained. 332 patients from 11 countries participated in the pilot study (accurate data from 290 patients and 124 out of 128 retests). One item was deleted due to non-response > 10%, 3 items because of a CHI <0.3 and 3 items due to insufficient loading. The final version (IHAE-QoL v.2.0) includes 25 items addressing 7 dimensions. Good coefficients and correlations were obtained: Cronbach's alpha 0.63-0.88, test-retest reliability 0.87, convergent validity with SF-36 (PCS 0.63, MCS 0.53, p<0.01) and external criterion validity with presence of symptoms, maintenance treatment and psychiatric care (p<0.001) among others. CONCLUSIONS: The IHAE-QoL shows good reliability and validity evidences and is available in 13 languages. | wrong publication type |
| 40 | Validation of the international quality of life questionnaire for hereditary angioedema | 2013 | Allergy Eur. J. Allergy Clin. Immunol. | Prior, N. and Remor, E. and PÃ©rez-FernÃ¡ndez, E. and GÃ³mez-Traseira, C. and Caminoa, M. and GayÃ¡, F. and Aabom, A. and Aberer, W. and Betschel, S. and Bygum, A. and Csuka, D. and Farkas, H. and Groffik, A. and Gomide, M. and Grumach, A. and Leibovich, I. and Malbran, A. and Mihaly, E. and Moldovan, D. and Obtulowicz, K. and Porebski, G. and Rayonne, C. and Reshef, A. and Staubach, P. and Wiednig, M. and Caballero, T. | Background: To the best of our knowledge no specific health-related Quality of Life (HRQoL) questionnaire for hereditary angioedema due to C1 inhibitor deficiency (HAE-C1INH) is published. After the developmental study, a draft version of an international HRQoL questionnaire for HAE (IHAE-QoL) with 44 items has been achieved. Method: International pilot study was performed in 11 countries. Item and psychometric analysis was carried out. First, criteria for item omission were a percentage of no response >10% or corrected homogeneity index (CHI) < 0.3. Second, exploratory factor analysis (EFA) was performed to check if theoretical model fitted the empirical data. Items with factor loadings < 0.4 were omitted. In addition, internal consistency and test-retest reliability were calculated. Construct validity evidences were also assessed. Results: 332 patients from 11 countries participated and accurate data were obtained from 290: Argentine 16 patients, Austria 18, Brazil 34, Canada 21, Denmark 27, Germany 42, Hungary 38, Israel 9, Poland 22, Romania 19 and Spain 44. Retest phase data were obtained from 119 patients. Item selection process led us to omit one item by a non-response rate above 10% and 3 items by a CHI <0.3. After EFA, three items were omitted due to insufficient factor loadings (<0.40). The final version of the questionnaire included 25 items addressing seven dimensions: treatment difficulties, physical functioning and health, role-emotional and social functioning, disease- related stigma, concern about offspring, perceived control over illness and mental health. Cronbach's alpha per dimension varied from 0.63 to 0.88. Regarding test-retest reliability, 37 patients were considered stable and intraclass correlation coefficient was 0.87. Conclusion: The 25-items version of IHAE-QoL, in 11 language versions, shows good reliability and evidences of validity, and is ready to use in research. | wrong publication type |
| 41 | Reliability and validity of the Wolfram Unified Rating Scale (WURS) | 2012 | Orphanet J. Rare Dis. | Nguyen, C. and Foster, E.R. and Paciorkowski, A.R. and Viehoever, A. and Considine, C. and Bondurant, A. and Marshall, B.A. and Hershey, T. | Background: Wolfram syndrome (WFS) is a rare, neurodegenerative disease that typically presents with childhood onset insulin dependent diabetes mellitus, followed by optic atrophy, diabetes insipidus, deafness, and neurological and psychiatric dysfunction. There is no cure for the disease, but recent advances in research have improved understanding of the disease course. Measuring disease severity and progression with reliable and validated tools is a prerequisite for clinical trials of any new intervention for neurodegenerative conditions. To this end, we developed the Wolfram Unified Rating Scale (WURS) to measure the severity and individual variability of WFS symptoms. The aim of this study is to develop and test the reliability and validity of the Wolfram Unified Rating Scale (WURS). Methods. A rating scale of disease severity in WFS was developed by modifying a standardized assessment for another neurodegenerative condition (Batten disease). WFS experts scored the representativeness of WURS items for the disease. The WURS was administered to 13 individuals with WFS (6-25 years of age). Motor, balance, mood and quality of life were also evaluated with standard instruments. Inter-rater reliability, internal consistency reliability, concurrent, predictive and content validity of the WURS were calculated. Results: The WURS had high inter-rater reliability (ICCs>.93), moderate to high internal consistency reliability (Cronbach's Î± = 0.78-0.91) and demonstrated good concurrent and predictive validity. There were significant correlations between the WURS Physical Assessment and motor and balance tests (rs>.67, p<.03), between the WURS Behavioral Scale and reports of mood and behavior (rs>.76, p<.04) and between WURS Total scores and quality of life (rs=-.86, p=.001). The WURS demonstrated acceptable content validity (Scale-Content Validity Index=0.83). Conclusions: These preliminary findings demonstrate that the WURS has acceptable reliability and validity and captures individual differences in disease severity in children and young adults with WFS. Â© 2012 Nguyen et al. | Wrong outcome |
| 42 | Development of a measure of self-concept in youth diagnosed with Familial Adenomatous Polyposis (FAP), a heritable condition | 2011 | Psycho-Oncology | Esplen, M.J. and Berk, T. and Stuckless, N. and Lefebvre, A. and Wong, J. | BACKGROUND: The diagnosis of FAP at a young age may alter a person's self- concept, or sense of self, which may impact on an individual's wellbeing and screening behaviors. The purpose of this study is to develop and validate an instrument for measuring the impact of being diagnosed with Familial Adenomatous Polyposis (FAP) on an adolescent's selfconcept. METHOD: The study is being conducted in two phases: Phase I - Item Generation and Refinement and Phase II - Scale Selection and Initial Validation. RESULTS: We have completed Phase I involving focus groups and interviews with adolescents between 12 years and 18 years who have been diagnosed with FAP. Phase II is currently ongoing. To date 77 adolescents have completed the candidate self-concept scale and validating measures. The candidate scale items reflect the following themes: Self in relation to other, Body image, Vulnerability, Resilience, Social stigma, Sexuality and Intimacy and future sense of self. The presentation will focus on the scale construction, statistical results of subscales and present the final scale. CONCLUSIONS: There are unique issues related to self concept that are relevant in considering the impact of youth onset genetic disorder. RESEARCH IMPLICATIONS: This is the first scale designed for youth with FAP and will contribute to the empirical literature to assist further research on genetic conditions. The scale could be used to inform future studies on the psychosocial impact of FAP and assist with planning clinical interventions to improved adjustment and quality of life. | wrong publication type |
| 43 | Validation of the Huntington clinical self-reported instrument (H-CSRI), a clinimetric patient assessed scale for patients with Huntington's disease | 2011 | Clin. Genet. | Clay, E. and Dorey, J. and Toumi, M. and Tedroff, J. and Squitieri, F. and De Nicola, N. and Verny, C. | Introduction: Longitudinal collection of data on Huntington's disease (HD) is difficult because of the low prevalence and the small number of specialized centers. The creation of a specific self-administered clinical questionnaire would make patient clinical data collection easier and allow a remote patient follow- up and integrate the patient perspective. The objective of this study is to validate a new self-reported clinical instrument for HD patients. Methods: The H-CSRI, a self-reported instrument based on the Unified Huntington's Disease Rating Scale (UHDRS) and developed with the expertise of a neurologist and a psychiatrist, was administered in a cross-sectional study. The H-CSRI includes three subscales assessing motor, functional and behavioural symptoms. Classical test theory and item response theory were used to assess its clinimetric properties. Results: Among 311 patients, item response rates range from 86% to 99%. There was a floor effect on items related to psychotic disorder in the behavioral dimension. The H-CSRI showed an acceptable reliability (Cronbach's alpha > 0.74). Factor analyses demonstrated a satisfactory construct validity. Moreover, the item internal consistency and item discriminant validity criteria were met. The differential item functioning analyses showed no item bias between the two countries and between genders. Convergence with UHDRS will be reported. Conclusions: These data support the validity of the H-CSRI to assess the health status for patients with HD and integrate the patient perspective. | wrong publication type |
| 44 | Quality of life in Brazilian patients with Sickle cell disease: Preliminary reliability report of the SF-36 survey | 2009 | Haematologica | Loureiro, M.M. and Biasoli, I. and Viana, A. and Soares, A.R. and Baptista, R.L. and Brabo, E. and Pulcheri, W. and Werneck, G. and Spector, N. | Background. Sickle cell disease (SCD) is one of the most common genetic disorders in Brazil and deeply compromises the quality of life (QOL) of those who are afflicted. We have evaluated the reliability of the Short Form 36 survey (SF-36) in our patients with sickle cell disease. Design and Methods. A validated Brazilian Portuguese version of the SF-36 was administered to patients with sickle cell disease in the outpatient clinic of the University Hospital. The domains evaluated by the SF-36 questionnaire cover physical function, physical and emotional role function, bodily pain, vitality, social function, mental health, and general health. Reliability (internal consistency) of all scales and of each domain were evaluated with the Cronbach's & coefficient. We also assessed whether gender and genotype had any influence on the SF-36 subscale results. The Mann Whitney test was used to compare the scale and subscales between two groups. Results. Eighty-eight patients responded the SF-36. The median age was 23 years (13-60), 57% were female, and 80% were black. Genotypes were SS (78.4%), Sb thalassemia (11.4%) and SC (10.2%). The internal consistency of items in the SF-36 scale was 0.85, and the internal consistency of domains were 0.85 (physical function), 0.81 (physical role function), 0.82 (emotional role function), 0.85 (bodily pain), 0.79 (vitality), 0.60 (social function), 0.85 (mental health) and 0.78 (general health). SS patientes and women have significantly lower scales (481 vs. 572 | Wrong outcome |
| 45 | EQ-5D and SF-6D health utility scores in patients with spinal and bulbar muscular atrophy. | 2023 | The European journal of health economics : HEPAC : health economics in prevention and care | Xu RH and Lu M and Zhang S and Dong D | OBJECTIVE: This study assessed patient-reported health-related quality of life (HRQoL) using two generic preference-based measures in Chinese patients with spinal and bulbar muscular atrophy (SBMA) and identified demographic and clinical determinants of health utility scores in this population. METHODS: This study used cross-sectional data of 212 Chinese patients with SBMA who completed both the EQ-5D and SF-6D. Association between response to EQ-5D and SF-6D dimensions was examined using Spearman's correlation coefficient, and the association between the two utility scores was assessed using Pearson's correlation coefficient. The variations in utility scores across patients in different subgroups were compared using one-way ANOVA. Bland-Altman (B-A) plot was used to assess the agreement of utility scores between EQ-5D and SF-6D. A multivariate Tobit regression model was employed to estimate the association between utility scores and the presence of symptoms and chronic conditions. RESULTS: The mean utility scores for the EQ-5D and SF-6D were 0.54 and 0.56, respectively. The hypothesized correlation between the EQ-5D and SF-6D dimensions ranged from 0.31 to 0.58, and the correlation between their utility scores was 0.64. An acceptable agreement between EQ-5D and SF-6D utility scores was identified by B-A plot. Patients with chronic diseases, misdiagnosis, high financial burden, and several clinical symptoms were highly likely to report a low health utility score. CONCLUSIONS: This study is the first to investigate the HRQoL of patients with SBMA worldwide. The estimated health utility scores for EQ-5D and SF-6D can be utilized as baseline data for future cost-utility analyses of SBMA-related interventions. | wrong study design |
| 46 | Evaluating the Patient-Reported Outcomes Measurement Information System scales in acute intermittent porphyria. | 2020 | Genetics in medicine : official journal of the American College of Medical Genetics | Naik H and Overbey JR and Montgomery GH and Winkel G and Balwani M and Anderson KE and Bissell DM and Bonkovsky HL and Phillips JD and Wang B and McGuire B and Keel S and Levy C and Erwin A and Desnick RJ | PURPOSE: Acute intermittent porphyria (AIP) is a rare inborn error of heme biosynthesis characterized by life-threatening acute attacks. Few studies have assessed quality of life (QoL) in AIP and those that have had small sample sizes and used tools that may not have captured important domains. METHODS: Baseline data from the Porphyrias Consortium's Longitudinal Study were obtained for 259 patients, including detailed disease and medical history data, and the following Patient-Reported Outcomes Measurement Information System (PROMIS) scales: anxiety, depression, pain interference, fatigue, sleep disturbance, physical function, and satisfaction with social roles. Relationships between PROMIS scores and clinical and biochemical AIP features were explored. RESULTS: PROMIS scores were significantly worse than the general population across all domains, except depression. Each domain discriminated well between asymptomatic and symptomatic patients with symptomatic patients having worse scores. Many important clinical variables like symptom frequency were significantly associated with domain scores in univariate analyses, showing responsiveness of the scales, specifically pain interference and fatigue. However, most regression models only explained ~20% of the variability observed in domain scores. CONCLUSION: Pain interference and fatigue were the most responsive scales in measuring QoL in this AIP cohort. Future studies should assess whether these scales capture longitudinal disease progression and treatment response. | wrong study design |
