# Supplementary material for: Self-reported perspective in rare genetic diseases: a systematic review of patient-reported outcome measures classified using the international classification of functioning, disability and health framework
Source: J Patient Rep Outcomes. 2026 Jul 25;10:141. doi: 10.1186/s41687-026-01155-5 (PMC13462012; doi:10.1186/s41687-026-01155-5)
Supplement: Supplementary file 3 [file 41687_2026_1155_MOESM3_ESM.docx]

| **Author et al. (year)** | **Scale (acronym)** | **Sample Size** | **Population** | **Content validity** | **Structural validity** | **Internal consistency** | **Cross-cultural validity** | **Reliability** | **Measurement error** | **Criterion validity** | **Construct validity** | **Respons.** |
| --- | --- | --- | --- | --- | --- | --- | --- | --- | --- | --- | --- | --- |
| Anttila, 2021 | Questionnaire for People with Skeletal Dysplasias | 14 | Skeletal Dysplasia | D ? |  |  |  |  |  |  |  |  |
| Asnani, 2009 | World Health Organization Quality of Life - BREF (WHOQOL-BREF) - Physical subscale | 491 | Sickle Cell Disease |  |  | VG + |  |  |  |  | VG - |  |
| Asnani, 2009 | World Health Organization Quality of Life - BREF (WHOQOL-BREF) - Psychological subscale | 491 | Sickle Cell Disease |  |  | VG + |  |  |  |  | VG - |  |
| Asnani, 2009 | World Health Organization Quality of Life - BREF (WHOQOL-BREF) Social relations subscale | 491 | Sickle Cell Disease |  |  | VG - |  |  |  |  | VG - |  |
| Asnani, 2009 | World Health Organization Quality of Life - BREF (WHOQOL-BREF) - Environment subscale | 491 | Sickle Cell Disease |  |  | VG + |  |  |  |  | VG - |  |
| Asnani, 2009 | World Health Organization Quality of Life - BREF (WHOQOL-BREF) | 491 | Sickle Cell Disease |  |  | VG + |  |  |  |  | VG - |  |
| Asnani, 2009 | 36‑item Short‑Form Health Survey (SF‑36) - Physical health | 491 | Sickle Cell Disease |  |  | VG + |  |  |  |  | VG - |  |
| Asnani, 2009 | 36‑item Short‑Form Health Survey (SF‑36) - Mental health | 491 | Sickle Cell Disease |  |  | VG + |  |  |  |  | VG - |  |
| Asnani, 2009 | 36‑item Short‑Form Health Survey (SF‑36) - Role llimitations | 491 | Sickle Cell Disease |  |  | VG + |  |  |  |  | VG - |  |
| Asnani, 2009 | 36‑item Short‑Form Health Survey (SF‑36) - Total score | 491 | Sickle Cell Disease |  |  | VG + |  |  |  |  | VG - |  |
| Asnani, 2009 | Flanagan's quality of life scale (QOLS) | 491 | Sickle Cell Disease |  |  | VG + |  |  |  |  | VG - |  |
| Audo, 2023 | Visual Symptom and Impact Outcomes patient-reported outcome (ViSIO-PRO) | 44 | Retinitis Pigmentosa (RP) Leber Congenital Amaurosis (LCA) | D ? |  |  |  |  |  |  |  |  |
| Behan, 2017 | Quality of Life Primary Ciliary Dyskinesia (QOL-PCD) - Physical functioning | 72 | Primary Ciliary Dyskinesia |  |  | VG + |  | A + | A + |  | A + |  |
| Behan, 2017 | Quality of Life Primary Ciliary Dyskinesia (QOL-PCD) - Emotional functioning | 72 | Primary Ciliary Dyskinesia |  |  | VG + |  | A + | A + |  | A + |  |
| Behan, 2017 | Quality of Life Primary Ciliary Dyskinesia (QOL-PCD) - Treatent functioning/burden | 72 | Primary Ciliary Dyskinesia |  |  | VG + |  | A + | A + |  | A + |  |
| Behan, 2017 | Quality of Life Primary Ciliary Dyskinesia (QOL-PCD) - social functioning | 72 | Primary Ciliary Dyskinesia |  |  | VG + |  | A + | A + |  | A - |  |
| Behan, 2017 | Quality of Life Primary Ciliary Dyskinesia (QOL-PCD) - Role functioning | 72 | Primary Ciliary Dyskinesia |  |  | VG + |  | A + | A + |  | A + |  |
| Behan, 2017 | Quality of Life Primary Ciliary Dyskinesia (QOL-PCD) - Health perception | 72 | Primary Ciliary Dyskinesia |  |  | VG + |  | A + | A + |  | A + |  |
| Behan, 2017 | Quality of Life Primary Ciliary Dyskinesia (QOL-PCD) - Vitality | 72 | Primary Ciliary Dyskinesia |  |  | VG + |  | A + | A + |  | A + |  |
| Behan, 2017 | Quality of Life Primary Ciliary Dyskinesia (QOL-PCD) - Upper respiratory symptoms | 72 | Primary Ciliary Dyskinesia |  |  | VG + |  | A + | A + |  | A + |  |
| Behan, 2017 | Quality of Life Primary Ciliary Dyskinesia (QOL-PCD) - Lower respiratory symptoms | 72 | Primary Ciliary Dyskinesia |  |  | VG + |  | A + | A + |  | A + |  |
| Behan, 2017 | Quality of Life Primary Ciliary Dyskinesia (QOL-PCD) - Hear and hearing symptoms | 72 | Primary Ciliary Dyskinesia |  |  | VG + |  | A + | A + |  | A - |  |
| Behan, 2019 | Quality of Life Primary Ciliary Dyskinesia (QOL-PCD) - Physical functioning (Adolescent version) | 71; 17 test-retest reliability | Primary Ciliary Dyskinesia |  |  | VG + |  | VG + |  |  | VG + |  |
| Behan, 2019 | Quality of Life Primary Ciliary Dyskinesia (QOL-PCD) - Emotional functioning - (Adolescent version) | 71; 17 test-retest reliability | Primary Ciliary Dyskinesia |  |  | VG + |  | VG + |  |  | VG + |  |
| Behan, 2019 | Quality of Life Primary Ciliary Dyskinesia (QOL-PCD) - Treatent functioning/burden - (Adolescent version) | 71; 17 test-retest reliability | Primary Ciliary Dyskinesia |  |  | VG + |  | VG + |  |  | VG + |  |
| Behan, 2019 | Quality of Life Primary Ciliary Dyskinesia (QOL-PCD) - social functioning - (Adolescent version) | 71; 17 test-retest reliability | Primary Ciliary Dyskinesia |  |  | VG + |  | VG + |  |  | VG - |  |
| Behan, 2019 | Quality of Life Primary Ciliary Dyskinesia (QOL-PCD) - Role - (Adolescent version) | 71; 17 test-retest reliability | Primary Ciliary Dyskinesia |  |  | VG + |  | VG + |  |  | VG + |  |
| Behan, 2019 | Quality of Life Primary Ciliary Dyskinesia (QOL-PCD) - Vitality - (Adolescent version) | 71; 17 test-retest reliability | Primary Ciliary Dyskinesia |  |  | VG + |  | VG + |  |  | VG - |  |
| Behan, 2019 | Quality of Life Primary Ciliary Dyskinesia (QOL-PCD) - Upper respiratory symptoms - (Adolescent version) | 71; 17 test-retest reliability | Primary Ciliary Dyskinesia |  |  | VG + |  | VG + |  |  | VG - |  |
| Behan, 2019 | Quality of Life Primary Ciliary Dyskinesia (QOL-PCD) - Lower respiratory symptoms - (Adolescent version) | 71; 17 test-retest reliability | Primary Ciliary Dyskinesia |  |  | VG + |  | VG + |  |  | VG - |  |
| Behan, 2019 | Quality of Life Primary Ciliary Dyskinesia (QOL-PCD) - Ear and hearing symptoms - (Adolescent version) | 71; 17 test-retest reliability | Primary Ciliary Dyskinesia |  |  | VG + |  | VG + |  |  | VG - |  |
| Behan, 2019 | Quality of Life Primary Ciliary Dyskinesia (QOL-PCD) - Physical functioning (Children version) | 85 validation; 13 test-retest reliability | Primary Ciliary Dyskinesia |  |  | VG + |  | VG - |  |  | VG - |  |
| Behan, 2019 | Quality of Life Primary Ciliary Dyskinesia (QOL-PCD) - Emotional functioning -(Children version) | 85 validation; 13 test-retest reliability | Primary Ciliary Dyskinesia |  |  | VG - |  | VG - |  |  | VG - |  |
| Behan, 2019 | Quality of Life Primary Ciliary Dyskinesia (QOL-PCD) - Treatent functioning/burden - (Children version) | 85 validation; 13 test-retest reliability | Primary Ciliary Dyskinesia |  |  | VG - |  | VG + |  |  | VG - |  |
| Behan, 2019 | Quality of Life Primary Ciliary Dyskinesia (QOL-PCD) - social functioning - (Children version) | 85 validation; 13 test-retest reliability | Primary Ciliary Dyskinesia |  |  | VG - |  | VG + |  |  | VG - |  |
| Behan, 2019 | Quality of Life Primary Ciliary Dyskinesia (QOL-PCD) - Upper respiratory symptoms - (Children version) | 85 validation; 13 test-retest reliability | Primary Ciliary Dyskinesia |  |  | VG + |  | VG + |  |  | VG - |  |
| Behan, 2019 | Quality of Life Primary Ciliary Dyskinesia (QOL-PCD) - Lower respiratory symptoms - (Children version) | 85 validation; 13 test-retest reliability | Primary Ciliary Dyskinesia |  |  | VG + |  | VG + |  |  | VG - |  |
| Behan, 2019 | Quality of Life Primary Ciliary Dyskinesia (QOL-PCD) - Ear and hearing symptoms - (Children version) | 85 validation; 13 test-retest reliability | Primary Ciliary Dyskinesia |  |  | VG - |  | VG + |  |  | VG - |  |
| Biolcati, 2021 | Erythropoietic Protoporphyria Quality of Life (EPP-QoL) Well-being | 355 | Erythropoietic Protoporphyria |  | D - | VG + | VG + | I - |  |  | D - |  |
| Biolcati, 2021 | Erythropoietic Protoporphyria Quality of Life (EPP-QoL) Symptoms | 355 | Erythropoietic Protoporphyria |  |  | VG + | VG + | I - |  |  | D + |  |
| Carlozzi, 2014 | Huntington's Disease Patien Reported Outcome-TRIAD (HD-PRO-TRIAD) - Cognition | 132 | Hungtington disease (HD) |  |  | VG + |  |  |  |  | A + |  |
| Carlozzi, 2014 | Huntington's Disease Patien Reported Outcome-TRIAD (HD-PRO-TRIAD) - Emotional/Behavioral Dyscontrol | 132 | Hungtington disease (HD) |  |  | VG + |  |  |  |  | A - |  |
| Carlozzi, 2014 | Huntington's Disease Patien Reported Outcome-TRIAD (HD-PRO-TRIAD) - Motor function | 132 | Hungtington disease (HD) |  |  | VG + |  |  |  |  | A + |  |
| Carlozzi, 2014 | Huntington's Disease Patien Reported Outcome-TRIAD (HD-PRO-TRIAD) - Total | 132 | Hungtington disease (HD) |  |  | VG + |  |  |  |  | A + |  |
| Carlozzi, 2016 | Huntington disease Quality of Life (HDQLIFE) - Speech | 536 | Hungtington disease (HD) | D ? | D - | VG ? | A - |  |  |  | A - |  |
| Carlozzi, 2016 | Huntington disease Quality of Life (HDQLIFE) - Swallowing | 536 | Hungtington disease (HD) |  |  | VG ? | A - |  |  |  | A - |  |
| Carlozzi, 2016 | Huntington disease Quality of Life (HDQLIFE) - chorea | 536 | Hungtington disease (HD) |  |  | VG ? | A - |  |  |  | A - |  |
| Carlozzi, 2016 | Huntington disease Quality of Life (HDQLIFE) - Death and dying | 536 | Hungtington disease (HD) |  |  | VG ? | A - |  |  |  | A - |  |
| Carlozzi, 2016 | Huntington disease Quality of Life (HDQLIFE) - Meaning and purpose | 536 | Hungtington disease (HD) |  |  | VG ? | A - |  |  |  | A - |  |
| Cestari, 2016 | Quality of Llife in Epidermolysis Bullosa (QoLEB) | 57 | Epidermolysis Bullosa |  |  | VG + |  | D + |  |  | A + |  |
| Clay, 2012 | Huntington Quality of Life Instrument (H-QoL-I) - Functioning | 252 | Hungtington disease (HD) | D ? | A + | VG + | D + |  |  |  | D + |  |
| Clay, 2012 | Huntington Quality of Life Instrument (H-QoL-I) - Psychology | 252 | Hungtington disease (HD) |  |  | VG + | D + |  |  |  | D + |  |
| Clay, 2012 | Huntington Quality of Life Instrument (H-QoL-I) - Socializing | 252 | Hungtington disease (HD) |  |  | VG + | D + |  |  |  | D - |  |
| Crawford, 2022 | Neurofibromatosis type 1 Adult Health-Related Quality of Life (NF1-AdQOL) - Emotions | 8 concept elicitation; 10 pre-test; 114 validation | Neurofibromatosis Type 1 | D + | I + | VG + |  | D + |  |  | D + |  |
| Crawford, 2022 | Neurofibromatosis type 1 Adult Health-Related Quality of Life (NF1-AdQOL) - Functioning | 8 concept elicitation; 10 pre-test; 114 validation | Neurofibromatosis Type 1 |  |  | VG + |  | D + |  |  | D + |  |
| Crawford, 2022 | Neurofibromatosis type 1 Adult Health-Related Quality of Life (NF1-AdQOL) - Symptoms | 8 concept elicitation; 10 pre-test; 114 validation | Neurofibromatosis Type 1 |  |  | VG + |  | D + |  |  | D + |  |
| Crossnohere, 2021 | EQ-5D-5L | 263 | Duchenne muscular dystrophy | I ? |  |  |  |  |  |  | VG + |  |
| De Vries, 2018 | Quality of Life in Epilepsy Inventory for Adolescents-48 (QOLIE-AD-48) - Memory / concentration | 37 | Tuberous sclerosis complex (TSC) |  |  | + VG |  |  |  |  | I - | D ? |
| De Vries, 2018 | Quality of Life in Epilepsy Inventory for Adolescents-48 (QOLIE-AD-48) - Epilepsy impact | 37 | Tuberous sclerosis complex (TSC) |  |  | + VG |  |  |  |  | I - | D ? |
| De Vries, 2018 | Quality of Life in Epilepsy Inventory for Adolescents-48 (QOLIE-AD-48) - Attitudes toward epilepsy | 37 | Tuberous sclerosis complex (TSC) |  |  | + VG |  |  |  |  | I - | D ? |
| De Vries, 2018 | Quality of Life in Epilepsy Inventory for Adolescents-48 (QOLIE-AD-48) - Social support | 37 | Tuberous sclerosis complex (TSC) |  |  | + VG |  |  |  |  | I - | D ? |
| De Vries, 2018 | Quality of Life in Epilepsy Inventory for Adolescents-48 (QOLIE-AD-48) - Stigma | 37 | Tuberous sclerosis complex (TSC) |  |  | + VG |  |  |  |  | I - | D ? |
| De Vries, 2018 | Quality of Life in Epilepsy Inventory for Adolescents-48 (QOLIE-AD-48) - School Behavior- Physical functioning | 37 | Tuberous sclerosis complex (TSC) |  |  | + VG |  |  |  |  | I - | D ? |
| De Vries, 2018 | Quality of Life in Epilepsy Inventory for Adolescents-48 (QOLIE-AD-48) - Health Perception | 37 | Tuberous sclerosis complex (TSC) |  |  | - VG |  |  |  |  | I - | D ? |
| De Vries, 2018 | Quality of Life in Epilepsy Inventory for Adolescents-48 (QOLIE-AD-48) - Total score | 37 | Tuberous sclerosis complex (TSC) |  |  | + VG |  |  |  |  | I - | D ? |
| De Vries, 2018 | Quality of Life in Epilepsy Inventory-31-Problems (QOLIE-31-P) - Total score | 33 | Tuberous sclerosis complex (TSC) |  |  | + VG |  |  |  |  | I - | D ? |
| De Vries, 2018 | Quality of Life in Epilepsy Inventory-31-Problems (QOLIE-31-P) - Emotonal well-being | 33 | Tuberous sclerosis complex (TSC) |  |  | + VG |  |  |  |  | I - | D ? |
| De Vries, 2018 | Quality of Life in Epilepsy Inventory-31-Problems (QOLIE-31-P) - Seizure worry | 33 | Tuberous sclerosis complex (TSC) |  |  | + VG |  |  |  |  | I - | D ? |
| De Vries, 2018 | Quality of Life in Epilepsy Inventory-31-Problems (QOLIE-31-P) - Energy fatigue | 33 | Tuberous sclerosis complex (TSC) |  |  | + VG |  |  |  |  | I - | D ? |
| De Vries, 2018 | Quality of Life in Epilepsy Inventory-31-Problems (QOLIE-31-P) - Cognitive | 33 | Tuberous sclerosis complex (TSC) |  |  | + VG |  |  |  |  | I - | D ? |
| De Vries, 2018 | Quality of Life in Epilepsy Inventory-31-Problems (QOLIE-31-P) - Overall QoL | 33 | Tuberous sclerosis complex (TSC) |  |  | + VG |  |  |  |  | I - | D ? |
| De Vries, 2018 | Quality of Life in Epilepsy Inventory-31-Problems (QOLIE-31-P) - Social function | 33 | Tuberous sclerosis complex (TSC) |  |  | - VG |  |  |  |  | I - | D ? |
| De Vries, 2018 | Quality of Life in Epilepsy Inventory-31-Problems (QOLIE-31-P) - Medication effects | 33 | Tuberous sclerosis complex (TSC) |  |  | + VG |  |  |  |  | I - | D ? |
| Dell, 2016 | Quality of Life Primary Ciliary Dyskinesia (QOL-PCD) - adolescents version | 69 | Primary Ciliary Dyskinesia | ? D |  |  |  |  |  |  |  |  |
| Dell, 2016 | Quality of Life Primary Ciliary Dyskinesia (QOL-PCD) - children version | 69 | Primary Ciliary Dyskinesia | ? D |  |  |  |  |  |  |  |  |
| Elstein, 2022 | Gaucher Disease Patient reported outcome measures (GD- PROMs) | 46 | Gaucher disease | D ? |  | I - |  | I - |  |  | I - |  |
| Fischer, 2023 | Visual Symptom and Impact Outcomes patient-reported outcome (ViSIO-PRO) | 83 | Retinitis Pigmentosa (RP) Leber Congenital Amaurosis (LCA) |  | D ? | VG + |  | VG + | VG + |  | I + |  |
| Fischer, 2023 | Visual Symptom and Impact Outcomes patient-reported outcome (ViSIO-PRO) | 83 | Retinitis Pigmentosa (RP) Leber Congenital Amaurosis (LCA) |  |  | VG + |  | VG + | VG + |  |  |  |
| Fischer, 2023 | Visual Symptom and Impact Outcomes patient-reported outcome (ViSIO-PRO) | 83 | Retinitis Pigmentosa (RP) Leber Congenital Amaurosis (LCA) |  |  | VG + |  | VG + | VG + |  |  |  |
| Fischer, 2023 | Visual Symptom and Impact Outcomes patient-reported outcome (ViSIO-PRO) | 83 | Retinitis Pigmentosa (RP) Leber Congenital Amaurosis (LCA) |  |  | VG + |  | VG + | VG + |  |  |  |
| Fischer, 2023 | Visual Symptom and Impact Outcomes patient-reported outcome (ViSIO-PRO) | 83 | Retinitis Pigmentosa (RP) Leber Congenital Amaurosis (LCA) |  |  | VG + |  | VG + | VG + |  |  |  |
| Godeau, 2023 | Children Low Flow Malformations Quality of Life (cLFM-QoL) | 75 | Low Flow Malformations | D ? | VG + | I + |  | D + | D ? |  | VG + |  |
| Gomide, 2013 | 36‑item Short‑Form Health Survey (SF‑36) - Physical functioning | 35 | Hereditary Angioedema (HAE) |  |  | VG + |  |  |  |  |  |  |
| Gomide, 2013 | 36‑item Short‑Form Health Survey (SF‑36) - Limitation physical health | 35 | Hereditary Angioedema (HAE) |  |  | VG + |  |  |  |  |  |  |
| Gomide, 2013 | 36‑item Short‑Form Health Survey (SF‑36) - Body pain | 35 | Hereditary Angioedema (HAE) |  |  | VG + |  |  |  |  |  |  |
| Gomide, 2013 | 36‑item Short‑Form Health Survey (SF‑36) - General Health perception | 35 | Hereditary Angioedema (HAE) |  |  | VG + |  |  |  |  |  |  |
| Gomide, 2013 | 36‑item Short‑Form Health Survey (SF‑36) - Vitality | 35 | Hereditary Angioedema (HAE) |  |  | VG + |  |  |  |  |  |  |
| Gomide, 2013 | 36‑item Short‑Form Health Survey (SF‑36) - Social functioning | 35 | Hereditary Angioedema (HAE) |  |  | VG - |  |  |  |  |  |  |
| Gomide, 2013 | 36‑item Short‑Form Health Survey (SF‑36) - Limitation emotional problems | 35 | Hereditary Angioedema (HAE) |  |  | VG + |  |  |  |  |  |  |
| Gomide, 2013 | 36‑item Short‑Form Health Survey (SF‑36) - Mental health | 35 | Hereditary Angioedema (HAE) |  |  | VG + |  |  |  |  |  |  |
| Groth, 2016 | Aplastic Anemia and/or Paroxysmal Nocturnal Hemoglobinuria-specific QoL (QLQ-AA/PNH) | Phase 1: 19; Phase 2: 30 | Aplastic Anemia and Paroxysmal Nocturnal Hemoglobinuria | A ? |  |  |  |  |  |  |  |  |
| Iversen, 2018 | 36‑item Short‑Form Health Survey (SF‑36) | 18 | Lymphedema cholestasis syndrome 1 |  |  | VG ? |  |  |  |  | VG - |  |
| Jahanshahi, 2023 | Neurofibromatosis type 1 Adult Health-Related Quality of Life (NF1-AdQOL) - Factor 1 | Concept elicitation: 40; Validation: 414; test-retest reliability: 30 | Neurofibromatosis Type 1 | D ? | VG - | VG + |  | D + |  |  |  |  |
| Jahanshahi, 2023 | Neurofibromatosis type 1 Adult Health-Related Quality of Life (NF1-AdQOL) - Factor 2 | Concept elicitation: 40; Validation: 414; test-retest reliability: 30 | Neurofibromatosis Type 1 |  |  | VG + |  |  |  |  |  |  |
| Jahanshahi, 2023 | Neurofibromatosis type 1 Adult Health-Related Quality of Life (NF1-AdQOL) - Factor 3 | Concept elicitation: 40; Validation: 414; test-retest reliability: 30 | Neurofibromatosis Type 1 |  |  | VG + |  |  |  |  |  |  |
| Jahanshahi, 2023 | Neurofibromatosis type 1 Adult Health-Related Quality of Life (NF1-AdQOL) - Factor 4 | Concept elicitation: 40; Validation: 414; test-retest reliability: 30 | Neurofibromatosis Type 1 |  |  | VG + |  |  |  |  |  |  |
| Jindal, 2017 | 36‑item Short‑Form Health Survey (SF‑36) - Physical functioning | 21 | Hereditary Angioedema (HAE) |  |  | VG + |  |  |  |  |  |  |
| Jindal, 2017 | 36‑item Short‑Form Health Survey (SF‑36) - Limitation physical health | 21 | Hereditary Angioedema (HAE) |  |  | VG + |  |  |  |  |  |  |
| Jindal, 2017 | 36‑item Short‑Form Health Survey (SF‑36) - Body pain | 21 | Hereditary Angioedema (HAE) |  |  | VG + |  |  |  |  |  |  |
| Jindal, 2017 | 36‑item Short‑Form Health Survey (SF‑36) - General Health perception | 21 | Hereditary Angioedema (HAE) |  |  | VG + |  |  |  |  |  |  |
| Jindal, 2017 | 36‑item Short‑Form Health Survey (SF‑36) - Vitality | 21 | Hereditary Angioedema (HAE) |  |  | VG + |  |  |  |  |  |  |
| Jindal, 2017 | 36‑item Short‑Form Health Survey (SF‑36) - Social functioning | 21 | Hereditary Angioedema (HAE) |  |  | VG + |  |  |  |  |  |  |
| Jindal, 2017 | 36‑item Short‑Form Health Survey (SF‑36) - Limitation emotional problems | 21 | Hereditary Angioedema (HAE) |  |  | VG + |  |  |  |  |  |  |
| Jindal, 2017 | 36‑item Short‑Form Health Survey (SF‑36) - Mental health | 21 | Hereditary Angioedema (HAE) |  |  | VG + |  |  |  |  |  |  |
| Jurecki, 2017 | Phenylketonuria – Quality Of Life (PKU-QOL) - Children version | 5 | Phenylketonuria | + D |  |  |  |  |  |  |  |  |
| Jurecki, 2017 | Phenylketonuria – Quality Of Life (PKU-QOL) - Adolescent Version | 5 | Phenylketonuria | + D |  |  |  |  |  |  |  |  |
| Jurecki, 2017 | Phenylketonuria – Quality Of Life (PKU-QOL) - Adult Version | 5 | Phenylketonuria | + D |  |  |  |  |  |  |  |  |
| Kanters, 2015 | EuroQoL-5D (EQ-5D) | 80 | Pompe Disease |  |  |  |  |  |  |  | I - | I - |
| Kanters, 2015 | ShortForm 6D (SF-6D) | 80 | Pompe Disease | + A |  |  |  |  |  |  | I - | I - |
| Koto, 2022 | Adult Fabry Disease Quality Of Life (AFQOL) | 83 | Fabry disease |  | I ? | VG ? |  |  |  |  | VG + |  |
| Koto, 2022 | Fabry-specific Pediatric Health and Pain Questionnaire (FPHPQ) - Factor 1 | 8 | Fabry disease |  | I ? | VG + |  |  |  | I ? |  |  |
| Koto, 2022 | Fabry-specific Pediatric Health and Pain Questionnaire (FPHPQ) - Factor 2 | 8 | Fabry disease |  | I ? | VG + |  |  |  | I ? |  |  |
| Koto, 2022 | Fabry-specific Pediatric Health and Pain Questionnaire (FPHPQ) - Factor 3 | 8 | Fabry disease |  | I ? | VG + |  |  |  | I ? |  |  |
| Kugler. 2026 | HED HRQoL | 24 | Hypohidrotic ectodermal dysplasia (HED) | + A |  |  |  |  |  |  |  |  |
| Landfeldt, 2018 | Pediatric Quality of Life Inventory 3.0 Neuromuscular Module (PedsQL NMM) | 278 | Duchenne muscular dystrophy |  | VG + | VG + |  |  |  |  |  |  |
| Lucas, 2015 | Quality of Life Primary Ciliary Dyskinesia (QOL-PCD) | 21 | Primary Ciliary Dyskinesia | ? D |  |  |  |  |  |  |  |  |
| Malina, 2024 | Health-related Quality Of Life in Hereditary Spastic Paraplegia (TreatHSP-QoL) - Genral QoL and attitudes to the disease | Concept elicitation: 36; Pre-test: 91; Validation 242 | Hereditary Spastic Paraplegia | D + | VG ? | VG + |  | D + |  |  | A ? |  |
| Malina, 2024 | Health-related Quality Of Life in Hereditary Spastic Paraplegia (TreatHSP-QoL) - Mobility and leisure time | Concept elicitation: 36; Pre-test: 91; Validation 242 | Hereditary Spastic Paraplegia |  |  | VG + |  | D + |  |  | A ? |  |
| Malina, 2024 | Health-related Quality Of Life in Hereditary Spastic Paraplegia (TreatHSP-QoL) - Associated symptoms | Concept elicitation: 36; Pre-test: 91; Validation 242 | Hereditary Spastic Paraplegia |  |  | VG - |  | D + |  |  | A ? |  |
| Malina, 2024 | Health-related Quality Of Life in Hereditary Spastic Paraplegia (TreatHSP-QoL) - Social life and occupation | Concept elicitation: 36; Pre-test: 91; Validation 242 | Hereditary Spastic Paraplegia |  |  | VG - |  | D + |  |  | A ? |  |
| Malina, 2024 | Health-related Quality Of Life in Hereditary Spastic Paraplegia (TreatHSP-QoL) | Concept elicitation: 36; Pre-test: 91; Validation 242 | Hereditary Spastic Paraplegia |  |  | VG + |  | D + |  |  | A ? |  |
| Mathias, 2024 | Erythropoietic Protoporphyria Impact Questionnaire (EPIQ) | 23 | Erythropoietic Protoporphyria | + A |  |  |  |  |  |  |  |  |
| McLean, 2013 | Neurofibromatosis Quality of Life (NFTI-QOL) | 77 | Neurofibromatosis type 2 | D ? |  | VG + |  |  |  |  | A + |  |
| Moroni, 2022 | pediatric Charcot-Marie-Tooth Quality of Life (pCMT-QoL) | 22 | Charcot Marie Tooth (CMT) |  |  |  |  | D + |  |  |  |  |
| Narita, 2024 | Gaucher Disease Patient reported outcome measures (GD- PROMs) | 33 | Gaucher disease | D ? |  | VG + |  | D + |  |  |  |  |
| Niedgeggen, 2019 | Aplastic Anemia and/or Paroxysmal Nocturnal Hemoglobinuria-specific QoL (QLQ-AA/PNH) - Social support | 48 | Aplastic Anemia and Paroxysmal Nocturnal Hemoglobinuria | D ? |  | VG - |  |  |  |  |  |  |
| Niedgeggen, 2019 | Aplastic Anemia and/or Paroxysmal Nocturnal Hemoglobinuria-specific QoL (QLQ-AA/PNH) - fear of progression and illness intrusiveness | 48 | Aplastic Anemia and Paroxysmal Nocturnal Hemoglobinuria | D ? |  | VG + |  |  |  |  |  |  |
| Niedgeggen, 2019 | Aplastic Anemia and/or Paroxysmal Nocturnal Hemoglobinuria-specific QoL (QLQ-AA/PNH) - infections | 48 | Aplastic Anemia and Paroxysmal Nocturnal Hemoglobinuria | D ? |  | VG + |  |  |  |  |  |  |
| Niedgeggen, 2019 | Aplastic Anemia and/or Paroxysmal Nocturnal Hemoglobinuria-specific QoL (QLQ-AA/PNH) - other symptoms | 48 | Aplastic Anemia and Paroxysmal Nocturnal Hemoglobinuria | D ? |  | VG + |  |  |  |  |  |  |
| Niedgeggen, 2019 | Aplastic Anemia and/or Paroxysmal Nocturnal Hemoglobinuria-specific QoL (QLQ-AA/PNH) - physical functioning | 48 | Aplastic Anemia and Paroxysmal Nocturnal Hemoglobinuria | D ? |  | VG + |  |  |  |  |  |  |
| Niedgeggen, 2019 | Aplastic Anemia and/or Paroxysmal Nocturnal Hemoglobinuria-specific QoL (QLQ-AA/PNH) - role functioning | 48 | Aplastic Anemia and Paroxysmal Nocturnal Hemoglobinuria | D ? |  | VG + |  |  |  |  |  |  |
| Niedgeggen, 2019 | Aplastic Anemia and/or Paroxysmal Nocturnal Hemoglobinuria-specific QoL (QLQ-AA/PNH) - emotional functioning | 48 | Aplastic Anemia and Paroxysmal Nocturnal Hemoglobinuria | D ? |  | VG + |  |  |  |  |  |  |
| Niedgeggen, 2019 | Aplastic Anemia and/or Paroxysmal Nocturnal Hemoglobinuria-specific QoL (QLQ-AA/PNH) - stigmatisation | 48 | Aplastic Anemia and Paroxysmal Nocturnal Hemoglobinuria | D ? |  | VG + |  |  |  |  |  |  |
| Niedgeggen, 2019 | Aplastic Anemia and/or Paroxysmal Nocturnal Hemoglobinuria-specific QoL (QLQ-AA/PNH) - body image | 48 | Aplastic Anemia and Paroxysmal Nocturnal Hemoglobinuria | D ? |  | VG + |  |  |  |  |  |  |
| Niedgeggen, 2019 | Aplastic Anemia and/or Paroxysmal Nocturnal Hemoglobinuria-specific QoL (QLQ-AA/PNH) | 48 | Aplastic Anemia and Paroxysmal Nocturnal Hemoglobinuria | D ? |  | VG + |  |  |  |  |  |  |
| Niedgeggen, 2019 | Aplastic Anemia and/or Paroxysmal Nocturnal Hemoglobinuria-specific QoL (QLQ-AA/PNH) | 48 | Aplastic Anemia and Paroxysmal Nocturnal Hemoglobinuria | D ? |  | VG + |  |  |  |  |  |  |
| Nutakki, 2013 | Pediatric Quality of Life Inventory (PedsQL™) | 80 | Patient-Reported Outcomes Measurement Information System (PROMIS) | ? D | - VG | + VG |  | + D |  |  |  |  |
| Oladapo, 2023 | Congenital Thrombotic Thrombocytopenic Purpura–Patient Experience Questionnaire (cTTP-PEQ) | 36 | Congenital thrombotic thrombocytopenic purpura (cTTP) |  |  | VG + |  | VG + |  |  |  |  |
| Oliviera Machado, 2026 | Pediatric Quality of Life Inventory (PedsQL™) | 42 | Neurofibromatosis type 1 | D + |  |  |  | I - |  |  |  |  |
| Palao-Ocharan. 2022 | 36‑item Short‑Form Health Survey (SF‑36) - Physical functioning | 290 | Hereditary Angioedema (HAE) |  |  | VG + |  | D + | A ? |  | A + |  |
| Palao-Ocharan. 2022 | 36‑item Short‑Form Health Survey (SF‑36) - Limitation physical health | 290 | Hereditary Angioedema (HAE) |  |  | VG + |  | D + | A ? |  | A + |  |
| Palao-Ocharan. 2022 | 36‑item Short‑Form Health Survey (SF‑36) - Body pain | 290 | Hereditary Angioedema (HAE) |  |  | VG + |  | D + | A ? |  | A + |  |
| Palao-Ocharan. 2022 | 36‑item Short‑Form Health Survey (SF‑36) - General Health perception | 290 | Hereditary Angioedema (HAE) |  |  | VG + |  | D + | A ? |  | A + |  |
| Palao-Ocharan. 2022 | 36‑item Short‑Form Health Survey (SF‑36) - Vitality | 290 | Hereditary Angioedema (HAE) |  |  | VG + |  | D + | A ? |  | A + |  |
| Palao-Ocharan. 2022 | 36‑item Short‑Form Health Survey (SF‑36) - Social functioning | 290 | Hereditary Angioedema (HAE) |  |  | VG + |  | D + | A ? |  | A + |  |
| Palao-Ocharan. 2022 | 36‑item Short‑Form Health Survey (SF‑36) - Limitation emotional problems | 290 | Hereditary Angioedema (HAE) |  |  | VG + |  | D + | A ? |  | A + |  |
| Palao-Ocharan. 2022 | 36‑item Short‑Form Health Survey (SF‑36) - Mental health | 290 | Hereditary Angioedema (HAE) |  |  | VG + |  | D + | A ? |  | A + |  |
| Pisciotta, 2020 | Charcot-Marie-Tooth Health Index (CMHI) | 30 | Charcot Marie Tooth (CMT) |  |  |  |  | I + |  |  | D - |  |
| Prior, 2012 | Hereditary Angioedema Quality of Life Questionnaire (HAE-QoL) | 45 | Hereditary Angioedema (HAE) | A + |  |  |  |  |  |  |  |  |
| Prior, 2016 | Hereditary Angioedema Quality of Life Questionnaire (HAE-QoL) - Treatment difficulties | 290 | Hereditary Angioedema (HAE) | D - | A + | D + |  | A + |  |  | A + |  |
| Prior, 2016 | Hereditary Angioedema Quality of Life Questionnaire (HAE-QoL) - Physical functioning and health | 290 | Hereditary Angioedema (HAE) |  |  | D + |  | A + |  |  | A + |  |
| Prior, 2016 | Hereditary Angioedema Quality of Life Questionnaire (HAE-QoL) - Disease-related stigma | 290 | Hereditary Angioedema (HAE) |  |  | D + |  | A + |  |  | A + |  |
| Prior, 2016 | Hereditary Angioedema Quality of Life Questionnaire (HAE-QoL) - Emotional role and social functioning | 290 | Hereditary Angioedema (HAE) |  |  | D + |  | A + |  |  | A + |  |
| Prior, 2016 | Hereditary Angioedema Quality of Life Questionnaire (HAE-QoL) - Concern abut offspring | 290 | Hereditary Angioedema (HAE) |  |  | D - |  | A + |  |  | A + |  |
| Prior, 2016 | Hereditary Angioedema Quality of Life Questionnaire (HAE-QoL) - Percieved control over illness | 290 | Hereditary Angioedema (HAE) |  |  | D + |  | A + |  |  | A + |  |
| Prior, 2016 | Hereditary Angioedema Quality of Life Questionnaire (HAE-QoL) - Mental health | 290 | Hereditary Angioedema (HAE) |  |  | D + |  | A + |  |  | A + |  |
| Prior, 2016 | Hereditary Angioedema Quality of Life Questionnaire (HAE-QoL) - Overall | 290 | Hereditary Angioedema (HAE) |  |  | D + |  | A + |  |  | A + |  |
| Ramchandren, 2015 | Disability Questionnaire (DQ) | 167 | Charcot Marie Tooth (CMT) |  | D ? | A ? |  | D ? |  |  | A + |  |
| Ramchandren, 2021 | pediatric Charcot–Marie–Tooth-specific Quality Of Llife (pCMT-QOL) | 398 | Charcot Marie Tooth (CMT) | I +- | VG - | VG ? |  |  | I + |  | A + |  |
| Regnault, 2015 | Phenylketonuria – Quality Of Life (PKU-QOL) - Children version | 92 | Phenylketonuria | D ? |  | VG - |  | A - |  |  | D ? |  |
| Regnault, 2015 | Phenylketonuria – Quality Of Life (PKU-QOL) - Adolescent Version | 110 | Phenylketonuria | D ? |  | VG - |  | A - |  |  | D ? |  |
| Regnault, 2015 | Phenylketonuria – Quality Of Life (PKU-QOL) - Adult Version | 104 | Phenylketonuria | D ? |  | VG - |  | A - |  |  | D ? |  |
| Salamon, 2024 | Instrument for Scoring Clinical Outcomes of Research for Epidermiolysis Bullosa Patient Questionanire (iscorEB-p) | 70 | Epidermolysis Bullosa |  | I + | VG + |  |  |  |  | VG + |  |
| Salamon, 2024 | Quality of Llife in Epidermolysis Bullosa (QoLEB) | 46 | Epidermolysis Bullosa | D ? | A + | VG + |  |  |  |  | D + |  |
| Spolak-Bobryk, 2022 | Quality of Life of Mastocytosis Scale (QLMS) | 85 | Mastocytosis |  | I + | VG + |  |  |  |  | D - |  |
| Tremblay, 2024 | Person-Reported Ataxia Impact Scale (PRAIS) | 105 | Autosomal recessive cerebellar ataxias (ARCAs) | D ? | I - | VG ? |  |  |  |  | I + |  |
| Truninger, 2024 | Pompe Qualty of Life 1.0 questionnaire (Pompe QoL) | 16 | Pompe Disease | D ? |  |  |  | D ? |  |  | A - |  |
| Turc, 2022 | Quality of Life-Hereditary hemorrhagic telangiectasia (QoL-HHT) | 228 | Hereditary hemorrhagic telangiectasia (HHT) | D ? | A + | VG + |  | I + |  |  | A + |  |
| van Kooten, 2024 | modified Rasch-Built Pompe-Specific Activity (mR-Pact) - ADL | 170 | Pompe Disease |  | A ? | VG + | D + |  | D + |  |  |  |
| van Kooten, 2024 | modified Rasch-Built Pompe-Specific Activity (mR-Pact) - Mobility | 170 | Pompe Disease |  | A ? | VG + | D + |  | D + |  |  |  |
| Vanya, 2023 | Angioedema Quality of Live Questionnaire (AE-QoL) | 64 | Hereditary Angioedema (HAE) | D - |  | VG + |  | A + | A ? |  | A + | A + |
| Villar Hernández, 2022 | Quality of Llife in Epidermolysis Bullosa (QoLEB) - Functional | 33 | Epidermolysis Bullosa | D ? | I + | VG + |  | D + |  |  | D + |  |
| Villar Hernández, 2022 | Quality of Llife in Epidermolysis Bullosa (QoLEB) - Emotional | 33 | Epidermolysis Bullosa | D ? | I + | VG + |  | D + |  |  | D + |  |
| Vinik, 2014 | Norfolk Quality of Life-Diabetic Neuropathy Questionnaire(QoL-DN) | 61 | Transthyretin Familial Amyloid Polineuropathy |  | I - |  |  | D - |  |  | A + |  |
| Xu, 2021 | EQ-5D-5L with two bolt-on item | 895 | Hemophilia (HE) | D ? |  |  |  |  |  |  | VG + |  |
| Xu, 2021 | EuroQoL-5D (EQ-5D)  ShortForm 6D (SF-6D) | 875 | Hemophilia (HE) |  |  |  |  |  |  |  | A + |  |
| Yazdanshenas, 2020 | QoLEB | 83 | Epidermolysis Bullosa | ? D |  | VG + |  |  |  |  | + D |  |
| Yuen, 2014 | QoLEB | 83 | Epidermolysis Bullosa |  |  | VG + |  | + D |  |  | + A |  |
